# Spatial chromatin architecture alteration by structural variations in human genomes at the population scale

**DOI:** 10.1186/s13059-019-1728-x

**Published:** 2019-07-30

**Authors:** Michal Sadowski, Agnieszka Kraft, Przemyslaw Szalaj, Michal Wlasnowolski, Zhonghui Tang, Yijun Ruan, Dariusz Plewczynski

**Affiliations:** 1 Centre of New Technologies, University of Warsaw, Banacha 2c, 02-097 Warsaw, Poland; 2 Faculty of Physics, University of Warsaw, Pasteura 5, 02-093 Warsaw, Poland; 3 Faculty of Mathematics and Information Science, Warsaw University of Technology, Koszykowa 75, 00-662 Warsaw, Poland; 4 Centre for Innovative Research, Medical University of Bialystok, Kilinskiego 1, 15-089 Bialystok, Poland; 5 I-BioStat, Hasselt University, Agoralaan building D, BE3590 Diepenbeek, Belgium; 6 Zhongshan School of Medicine, Sun Yat-sen University, Guangzhou, 510080 China; 7 The Jackson Laboratory for Genomic Medicine, 10 Discovery Drive, Farmington, CT 06032 USA

**Keywords:** Genomics, Chromatin architecture, Topologically associating domains, Chromatin loops, Genome regulation, Gene transcription, CCCTC-binding factor, RNA polymerase II, Biophysical modeling, Human

## Abstract

**Background:**

The number of reported examples of chromatin architecture alterations involved in the regulation of gene transcription and in disease is increasing. However, no genome-wide testing has been performed to assess the abundance of these events and their importance relative to other factors affecting genome regulation. This is particularly interesting given that a vast majority of genetic variations identified in association studies are located outside coding sequences. This study attempts to address this lack by analyzing the impact on chromatin spatial organization of genetic variants identified in individuals from 26 human populations and in genome-wide association studies.

**Results:**

We assess the tendency of structural variants to accumulate in spatially interacting genomic segments and design an algorithm to model chromatin conformational changes caused by structural variations. We show that differential gene transcription is closely linked to the variation in chromatin interaction networks mediated by RNA polymerase II. We also demonstrate that CTCF-mediated interactions are well conserved across populations, but enriched with disease-associated SNPs. Moreover, we find boundaries of topological domains as relatively frequent targets of duplications, which suggest that these duplications can be an important evolutionary mechanism of genome spatial organization.

**Conclusions:**

This study assesses the critical impact of genetic variants on the higher-order organization of chromatin folding and provides insight into the mechanisms regulating gene transcription at the population scale, of which local arrangement of chromatin loops seems to be the most significant. It provides the first insight into the variability of the human 3D genome at the population scale.

**Electronic supplementary material:**

The online version of this article (10.1186/s13059-019-1728-x) contains supplementary material, which is available to authorized users.

## Background

Around 20 million base pairs of a normal human genome (0.6%) are under structural variations, including deletions, duplications, insertions, and inversions. This makes structural variants (SVs) the most prominent source of genetic variation among human individual genomes.

The potential malicious effect of SVs has been recognized but almost solely associated with altering gene copy number and gene structure—a number of studies relate copy number variants (CNVs) affecting gene regions to cancer [1], intellectual disabilities [2], and predispositions to various health problems [3, 4]. The vast majority of genetic variation occurs, however, in non-coding regions. Over 95% of single-nucleotide polymorphisms (SNPs) identified by genome-wide association studies (GWAS) are located outside coding sequences [5]. Similarly, larger variants are significantly depleted in gene regions [6].

A part of the SVs emerging in non-coding regions alters genomic loci recognized by proteins which organize the human genome in the cell nuclear space. Recent studies provided some insights into the impact SVs can have on a spatial organization of the human genome. Examples of SVs altering the borders of TADs in EPHA4 locus and causing pathogenic phenotypes by enabling spatial contacts between formerly isolated genomic functional elements were reported [7]. Positions of TAD boundaries were proven useful for inferring cancer-related gene overexpression resulting from variation in *cis*-regulatory elements [8]. Accumulation of SVs proximal to the TAD boundary occupied by CTCF was postulated to cause enhancer hijacking and PRDM6 overexpression in medulloblastoma samples [9]. Hi-C maps were successfully used for the detection of large-scale rearrangements, which were reported as frequent in cancer cells [10]. Disruptions of chromosome neighborhoods were demonstrated—using CRISPR/Cas9 experiments—to activate proto-oncogenes [11]. An attempt was also made to model 3D chromatin structure including information on SVs and predicting enrichment/depletion of higher-order chromatin contacts caused by these variations [12]. Efficacy of the modeling method in predicting SV-induced ectopic contacts at the level of TADs was shown for EPHA4 locus.

However, to our knowledge, there was no genome-wide systematic study on the impact of SVs on genome spatial organization analyzing the level of individual chromatin loops. One of the most recent reviews on the topic [13] highlights the impact of SVs on genome spatial structure and the pathogenic potential of SVs altering the higher-order chromatin organization. Nonetheless, no attempt was made by the authors to assess what part of SVs emerging in normal human genomes causes functionally relevant chromatin spatial rearrangements, and no genome-wide data was presented on how SVs influence the chromatin 3D architecture.

The recent advancements in chromosome conformation capture techniques, namely high-throughput conformation capture (Hi-C) [14, 15] and Chromatin Interaction Analysis by Paired-End Tag Sequencing (ChIA-PET) [16, 17], resulted in the release of high-resolution chromatin interaction datasets. ChIA-PET, in particular, is able to capture individual chromatin contacts mediated by specific protein factors. In turn, the great effort of the 1000 Genomes Consortium led to the creation of the catalog of human genomic sequence variations [6] identified in over 2500 human samples from 26 populations.

Taking advantage of the high-quality ChIA-PET and population-scale SVs data, we discuss a mechanistic model of the impact of SVs on the chromatin looping structure, provide the first genome-wide analysis of this impact for the human genome, and model SV-induced changes in 3D genomic structures observed in human population.

In our analyses of the impact of SVs on the 3D chromatin organization of the human genome, we pay a specific attention to chromatin interactions associated with enhancer regions and gene promoters. These interactions are likely to play a distinguished role in the regulation of gene transcription in a mechanistic fashion, bringing the genes and the regulatory elements close together or separating them in the nuclear space of the cell. We observe an interesting interplay between such genomic interactions and SVs.

## Results

### 3D human genome

In this study, we use ChIA-PET interactions as a representation of the higher-order spatial organization of the human genome. ChIA-PET targeting on CTCF and RNAPII performed on the GM12878 cell line [17, 18] was selected as the most comprehensive ChIA-PET dataset for humans presently. CTCF was shown to be the key protein factor shaping the architecture of mammalian genomes [15, 19], whereas RNAPII is essential for gene transcription. Together, the ChIA-PET data of these two protein factors account for structural and functional aspects of the higher-order organization and multiscale folding of chromatin 10 nm fiber in the human cell nucleus [20]. It was postulated that pools of interacting CTCF/cohesin-mediated loop anchors form the structural foci, toward which interactions mediated by RNAPII draw genes for coordinated transcription [17]. We will further refer to these structural foci as interaction centers.

ChIA-PET generates high-resolution (~ 100 bp) genome-wide chromatin contact maps. It identifies two types of chromatin interactions mediated by specific protein factors. The first type is highly reliable enriched interactions which appear in the data as closely mapped on the genome clustered inter-ligation paired-end-tag products (PET clusters). The second type is singletons, which reflect higher-order topological proximity [17].

**Fig. 1:**
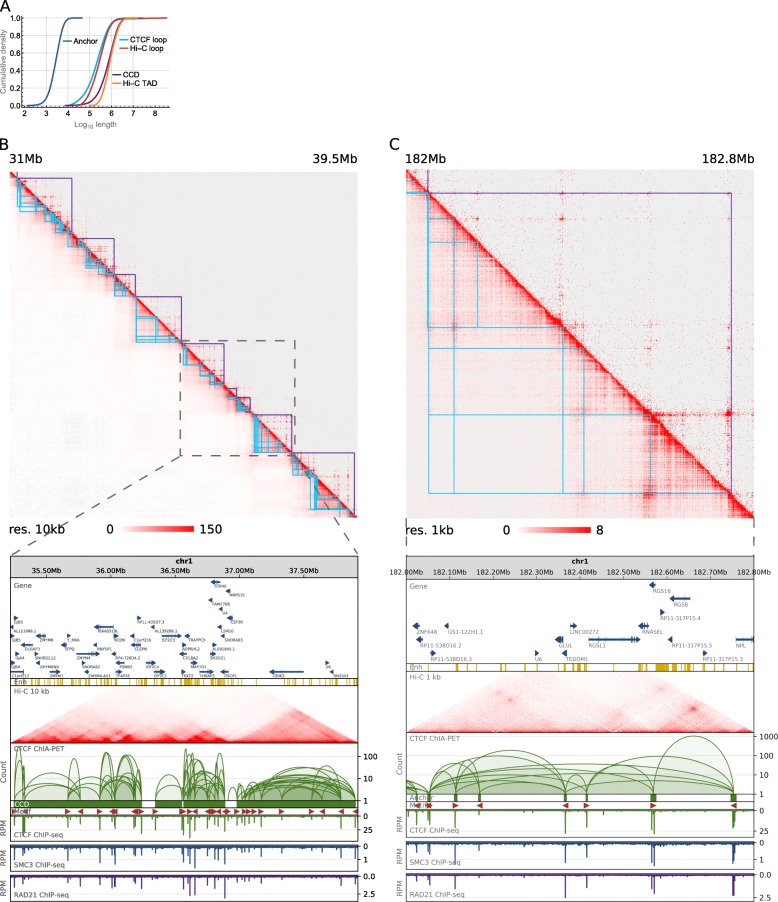
3D human genome. Data aggregation and comparison. **a** Cumulative density plot showing the genomic span distribution of genomic structural elements identified in ChIA-PET and HI-C. **b** Comparison of Hi-C (lower left) and ChIA-PET (upper right) heatmaps of a 8.5-Mb genomic region in 10 kb resolution. Annotation for chromatin loops (cyan) and domains (purple) from ChIA-PET is presented on the heatmaps. The same ChIA-PET data is shown in a browser view together with ChIP-seq tracks for CTCF and cohesin subunits. The height of an arc indicates the strength of an interaction, which is measured by the number of clustered individual inter-ligation paired-end-tag products. Annotations of genes (GENCODE v12) and enhancers (ChromHMM) are presented. Arrows at genes mark the direction of their transcription. **c** Similar to **b**, but a 0.8-Mb genomic region is presented in a heatmap in 1 kb resolution and in a browser view

We inspected the anchoring sites of PET clusters identified by the CTCF ChIA-PET experiment for the co-occupancy by CTCF and cohesin (SMC3 and RAD21 subunits), to select the set of high-quality chromatin interactions mediated by CTCF in GM12878 cell (see the “Methods” section). We identified 44,380 such pairwise interactions (Additional file 1: Table S1). The median length of genomic segments joined by these interactions is 2730 bp, and 99% of them are shorter than 10 kb (Fig. 1a). Nucleotide sequences of these segments usually contain multiple CTCF motifs. Chromatin loops formed by the CTCF interactions have lengths in the order of 100 kb (Fig. 1a).

The interactions mediated by CTCF are not uniformly distributed over the genome but rather form highly interacting, predominantly hundreds of kilobases-long chromatin blocks (which we will further refer to as chromatin contact domains (CCDs)) separated by segments of weak and rare contacts (gaps). Based on the CTCF ChIA-PET data, the genome of GM12878 cell was segmented into 2267 CCDs [18], and we adopt this segmentation in this study. The domains lengths vary from around 10 kb to few megabases with a median length of 750 kb. Only 1% of CCDs is longer than 2 Mb (Fig. 1a).

Even though CTCF ChIA-PET captures only the interactions mediated by the CTCF protein, it detects structural features exhibited by the non-specific Hi-C data [21]. It was shown that at a global scale, whole-chromosome Hi-C and ChIA-PET contact maps are highly correlated (Spearman’s correlation coefficient in the range of 0.7–0.9) [17]. Locally, ChIA-PET and Hi-C heatmaps identify a very similar landscape of genomic structures, both at the scale of topological domains (Fig. 1b) and chromatin loops (Fig. 1c). A large fraction of TADs identified in high-resolution Hi-C data are demarcated by anchors of chromatin loops highly enriched with CTCF and the cohesin subunits SMC3 and RAD21 [15]. Even though the borders of topological domains formed by CTCF loops identified in ChIA-PET data coincide with only a small fraction of anchors of those loops, they exhibit distinctively high levels of CTCF, SMC3, and RAD21 binding signals (Fig. 1b and Additional file 2: Figure S1). This underlines the specificity of those loci among CTCF loop anchors and is consistent with the findings based on Hi-C data. Furthermore, length distributions of chromatin loops and topological domains called from ChIA-PET data are concordant with the respective statistics for Hi-C (Fig. 1a). All this indicates that CTCF ChIA-PET dataset generated for GM12878 cell is a high-quality representation of the human 3D genome.

Following the authors of the dataset, we investigated the directionality of CTCF motifs in the anchors of the CTCF chromatin loops. Thirty-seven thousand two hundred eighty-nine out of the 44,380 PET clusters had motifs of unique orientation in both anchors. Among the 37,289, we found 24,181 (65%) interactions with motifs in the anchors having convergent orientation (convergent loops), 6118 (16%) interactions in tandem right orientation (tandem right loops), 6089 (16%) tandem left loops, and 901 (2%) divergent loops (see the “Methods” section). We adopted the coordinates of the outermost CTCF motifs in CCDs as indicators of their borders (see the “Methods” section).

The described ChIA-PET dataset is further used as the reference 3D genome of a human lymphoblastoid cell.

### Predicting chromatin architecture altered by SVs

It was demonstrated, by phasing CTCF PET clusters identified in GM12878, that allele-specific single-nucleotide variation in genome sequence can result in haplotype-specific chromatin topology [17].

**Fig. 2:**
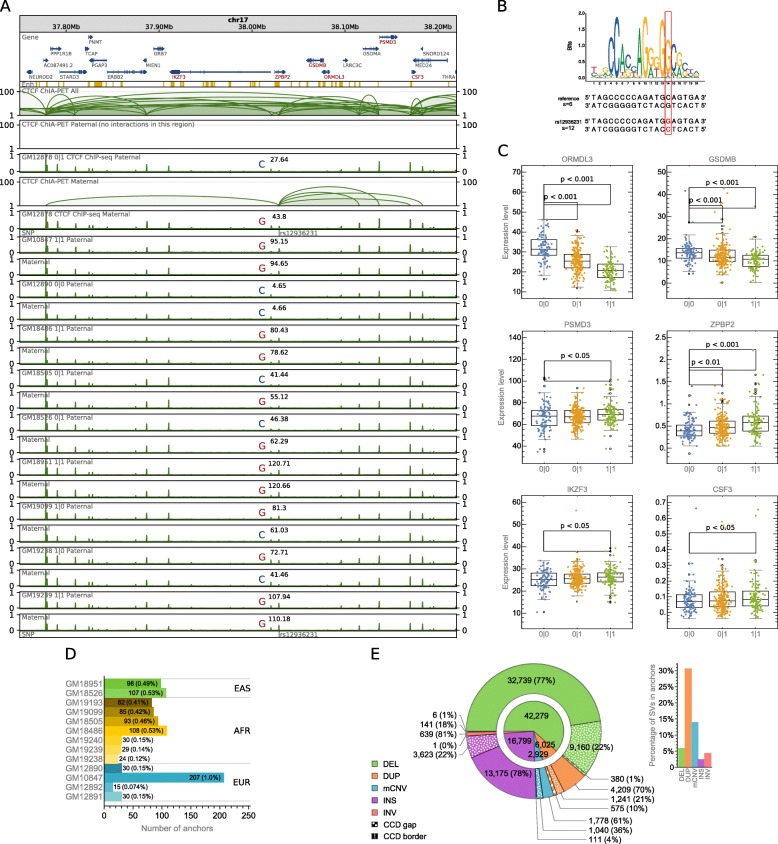
Predicting the impact of SVs on the chromatin topology. **a** Browser view of a 0.5-Mb genomic segment with asthma-associated SNP rs12936231 identified in a part of the human population. SNP rs12936231 alters the sequence of a CTCF motif involved in interactions. Haplotype-specific CTCF signals from 10 lymphoblastoid cells are presented along with haplotype-specific CTCF ChIA-PET interactions from GM12878 (only a subset of all interactions can be identified as specifically paternal/maternal as it is done based on allele-specific SNPs emerging at the interaction anchors). For each track, ChIP-seq signal values (originally in RPMs) were divided by the maximal value of the signal in the visualized region. Sum of the signal values over the genomic region occupied by the SNP-affected interaction anchor together with the genotype is marked in each signal track. **b** Comparison of sequences and scores of CTCF binding motifs carrying the reference C and the alternative G alleles of rs12936231. **c** Differences in gene transcription rates between genotypes set for rs12936231. Genes exhibiting differences in transcription which pass Mann-Whitney test with *p* value < 0.05 were reported. Center lines show the medians; box limits indicate the 25th and 75th percentiles; whiskers extend 1.5 times the interquartile range (IQR) from the 25th and 75th percentiles; outliers are represented by rings; far outliers (points beyond 3 times the IQR) are not represented by any element of box plots. *n* = 101, 227, 117 sample points. **d** CTCF anchors from GM12878 not intersected with CTCF ChIP-seq peaks identified in different lymphoblastoid cells. The anchors were filtered by consensus CTCF binding sites (see the “Methods” section). **e** Number of SVs, divided by type, intersecting (in case of interaction anchors), covering (in case of CCD boundaries), or contained in (in case of CCDs and CCD gaps) different genomic structural elements

We further show that relative values of haplotype-specific CTCF binding signals (see the “Methods” section) accurately reflect genotypes determined by this variation in a number of lymphoblastoid cell lines (Fig. 2a, b; Additional file 2: Figure S2A and S2B). Furthermore, CTCF binding profiles around CTCF interaction anchors of unchanged nucleotide sequences are very similar across the cells. The analogy between the changes in CTCF binding caused by anchor-targeting allele-specific SNPs between homologous chromosomes of GM12878 and among lymphoblastoid cells suggests that the major differences in chromatin topology between chromosomes of two lymphoblastoid cells are an effect of genetic variation and can be predicted based on genomic interactions identified in GM12878. Such predictions can in turn uncover causal relations underlying the associations observed between genetic variations and gene transcription rates (Fig. 2c).

In this study, we concentrate on predicting how SVs impact genome looping organization. SVs are the major source of sequence variation among human genomes and given their larger size have a higher potential than SNPs to induce changes in chromatin folding. They were also shown to contribute more than SNPs to variation in gene expression among human samples [22]. Chromatin contacts are thought to be largely invariant across individuals. To assess the level of conservation of CTCF-dependent genome architecture across individuals, we analyzed the abundance and arrangement of CTCF ChIP-seq peaks from 13 lymphoblastoid samples in genomic segments which were identified as CTCF-mediated interaction anchors in the GM12878 cell line. The ChIP-seq data originate from one study [23, 24] and include 5 samples of European ancestry (GM10847, GM12878, GM12890, GM12891, GM12892), 7 samples of African ancestry (GM18486, GM18505, GM19099, GM19193, GM19238, GM19239, GM19240), and 2 samples of East Asian ancestry (GM18526, GM18951). GM12891, GM12892, GM12878 and GM19239, GM19238 and GM19240 are families of father, mother, and child respectively. The datasets were rigorously filtered for comparisons (see the “Methods” section).

Our analysis shows that CTCF binding profiles at long-range interaction sites are highly similar across lymphoblastoid cells. Over 99% of interacting anchors occupied by CTCF peaks in GM12878 cell are supported by CTCF peaks in each of the 13 other samples (Fig. 2d). Moreover, the overall distribution of CTCFs involved in the formation of chromatin contacts is shared among individuals. For about 90% of all CTCF peaks identified in anchors of PET clusters in GM12878 cell, a CTCF peak in each of the compared cells can be found within a distance of 400 bp (Additional file 2: Figure S3). CTCF-interacting anchors and borders of CCDs identified in GM12878 cell are highly enriched with CTCF ChIP-seq peaks found in the other 13 lymphoblastoid cells (Additional file 2: Figure S4).

Similar analyses were performed for RNAPII-mediated interacting anchors using RNAPII ChIP-seq peaks (Additional file 2: Figure S5). Significantly bigger but moderate differences among samples were observed for this data.

Having tested the resemblance of genomic interaction site distribution in lymphoblastoid cells, we match SVs detected in human population in the 1000 Genomes Project [6] with the reference network of chromatin interactions to obtain individualized chromatin interaction patterns and assess the topological variability among human genomes.

There are 68,818 unique SVs deposited in the 1000 Genomes Catalog of Human Genetic Variation (CHGV) [25], including deletions, duplications, multiallelic CNVs (mCNVs), inversions, and insertions (Fig. 2e). Forty-four percent of them are shorter than 1 kb, and only 22% is longer than 10 kb (Additional file 2: Figure S6).

Most of the SVs reside inside CCDs, not intersecting borders of domains nor CTCF-mediated interaction anchors (Fig. 2e).

### Computational algorithm for modeling SV-induced chromatin conformational changes

**Fig. 3:**
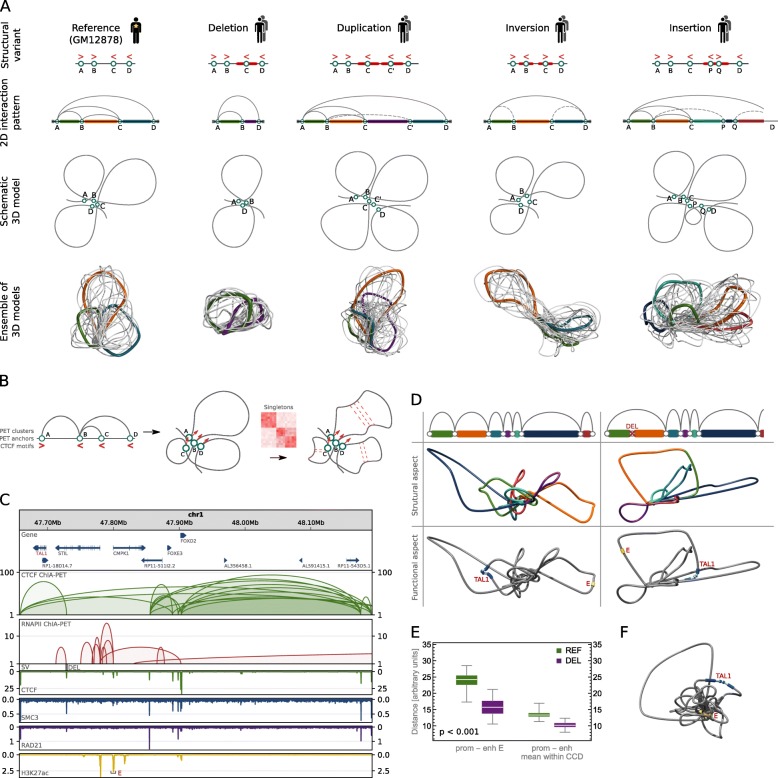
Computational algorithm for modeling topological alterations caused by SVs. **a** Predicted impact of particular SV types on looping structure of the genome. Simplified chromatin looping patterns and 3D models are presented for the reference and its SV-altered versions. **b** Scheme presenting the chromatin modeling method at the level of loops. The method uses PET clusters, singletons, and orientations of CTCF binding motifs to accurately model the genome looping structures. **c** Browser view of a topological domain containing TAL1 gene and a deletion causing its activation. The deletion removes CTCF insulating the TAL1 promoter from enhancer E. CTCF and RNAPII ChIA-PET interactions are shown along with ChIP-seq tracks for CTCF, cohesin subunits (SMC3 and RAD21), and H3K27ac which marks the enhancer E. **d** Models presenting 3D structure of the TAL1 locus without the deletion (left column) and with the deletion (right column). Schematic drawings of loops shown in **c** (first row); 3D models with loops colored as on schematic drawings (second row); 3D models with TAL1 and enhancer E marked (third row). **e** Distance in 3D Euclidian space between the TAL1 promoter and enhancer E and mean distance between the promoter and enhancers located in the same CCD. In green, distribution of distances calculated in 3D models of the reference structure (REF), in purple—in models with the deletion introduced (DEL). For each case, 100 models were generated. The differences between REF and DEL groups are statistically significant (*p* values much less than 0.001), see Fig. 2c for box plot description. **f** 3D model of the TAL1 locus including RNAPII-mediated chromatin interactions

While the SVs that miss the interacting binding sites in most cases have limited impact on the final structure (resulting only in shortening, or extending of the corresponding chromatin loops), the SVs that overlap the interacting sites may partially modify the interaction pattern and in turn cause serious changes of the 3D structure (Fig. 3a). Specifically, deletion removes an interacting anchor therefore deleting all chromatin loops mediated by this genomic site; duplication introduces a new interaction site, which has the same underlying sequence specificity to form chromatin loops as the original duplicated site; inversion which encircles a CTCF binding motif will revert its directionality therefore affecting the chromatin looping of the neighboring region; and finally, insertion containing CTCF motif enables new interaction sites capable of forming chromatin loops with other CTCF binding sites.

We earlier demonstrated that using CTCF ChIA-PET data, a 3D model of an averaged genome structure which recovers architectural features of the genome can be built [26]. Chromatin models constructed with our computational tool (3D-GNOME) can be used to illustrate the most probable arrangement of genomic structural elements in 3D space: from chromosomes, through topological domains, to individual chromatin loops (see the “Methods” section). 3D-GNOME uses PET clusters to position the binding sites relative to each other first and then employs singletons, orientations of the CTCF motifs, and biophysical constraints to accurately model the shape of individual chromatin loops (Fig. 3b).

We extended the 3D-GNOME modeling approach to include information on SVs in the recovery of 3D chromatin structures (see the “Methods” section). Our algorithm models individual chromatin loops, meaning that the remodeling effect of a genetic variant disrupting a single pair of interacting genomic segments will be represented in the model (Fig. 3a). 3D-GNOME is an optimization algorithm which returns models that fulfill spatial constraints coming from genomic interaction data. Typically, a number of solutions exist for a given set of constraints (Fig. 3a).

Analysis and modeling of genome organization levels of topological domains and chromatin loops are in the main scope of this study, as topological domains are believed to be the structural units regulating gene transcription by spatially isolating groups of enhancers and genes [7, 15, 17, 27]. In our opinion, our 3D models constitute a supportive insight into SV effects; their inspection can improve the understanding of functional impact and disease association of SVs.

As an example, deletion of a CTCF binding site insulating the promoter of TAL1 gene from regulatory elements adjacent to the CMPK1 promoter was shown by CRISPR/Cas9 experiments to cause activation of TAL1, an oncogenic driver of T cell acute lymphoblastic leukemia [11] (Fig. 3c). 3D structures of the TAL1 locus generated with our algorithm illustrate fusion of the TAL1 promoter with the enhancer regions inside the insulated neighborhood formed as a consequence of the deletion (Fig. 3d). 3D distances calculated from the models quantify the accessibility of transcription-enhancing elements for the TAL1 promoter. The 3D distance between the promoter and a strong enhancer in the CMPK1 promoter and the mean distance between the promoter and enhancers located in the same CCD decrease significantly after the deletion (Fig. 3e). The models show how the promoter and the enhancer are brought even closer together within the insulated neighborhood by RNA-mediated chromatin interactions (Fig. 3f). The models accurately illustrate the mechanisms pinpointed as causative for TAL1 overexpression based on extensive experimental testing [11]. This demonstrates that their inspection can give insights into the functional consequences of SVs.

The chromatin modeling method including SV information is provided as a web service at [28] together with a visualization tool.

### Topological impact of structural variations

**Fig. 4:**
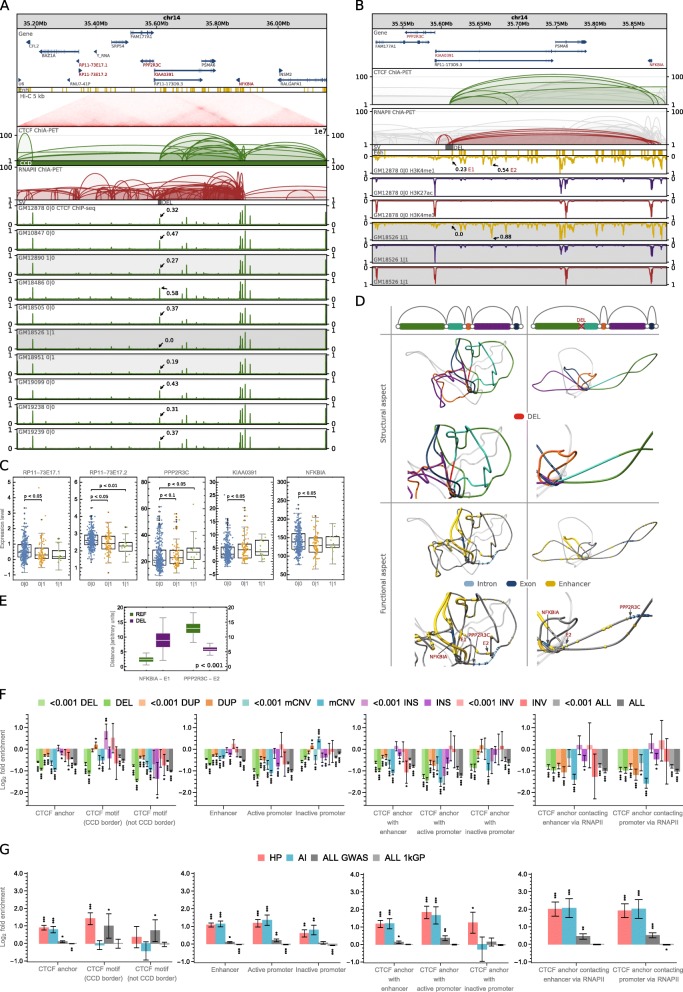
Impact of SVs on genome organization at the population scale. **a** Browser view of a 1-Mb genomic segment with a deletion identified in a part of the human population. The deletion removes a CTCF anchor with enhancer located in an intron of KIAA0391. CTCF ChIP-seq signals from 10 lymphoblastoid cells of different genotypes are presented for comparison. For each track, ChIP-seq signal values (originally in RPMs) were divided by the maximal value of the signal in the visualized region. The highest signal peak in the genomic region covered by the deletion is marked in each signal track. **b** Close-up on ChIA-PET interactions at the deletion site displayed above the ChIP-seq profiles of histone modifications for GM12878—no deletion and GM18526—homozygous deletion. H3K4me1 is primarily associated with active enhancers, H3K27ac—with active promoters and enhancers, H3K4me3—with promoters. Compare with Additional file 2: Figure S7. **c** Differences in gene transcription rates between genotypes defined by the deletion. Genes exhibiting the differences in transcription which pass Mann-Whitney test with *p* value < 0.1 were reported, see Fig. 2c for box plot description. *n* = 346, 85, 14 sample points. **d** 3D models of the domain shown in **a** without the deletion (left column) and with the deletion (right column). Schematic drawings of loops shown in **b** (first row); 3D models with loops colored as on schematic drawings (second row); 3D models with NFKBIA and PPP2R3C genes (arrows are pointing toward the TSSs) and enhancers marked (third row). Every picture has its duplicated zooming in on the deletion site. **e** Distance in 3D Euclidean space between the NFKBIA promoter and enhancer E1 and between the PPP2R3C promoter and enhancer E2. In green, distribution of distances calculated in 3D models of the reference structure (REF), in purple—in models with the deletion introduced (DEL). For each case, 100 models were generated. The differences between REF and DEL groups are statistically significant (*p* values much less than 0.001), see Fig. 2c for box plot description. **f** Enrichment/depletion of genomic structural elements with SVs of different types and of different VAF (VAF < 0.001 and VAF ≥ 0.001). In case of CCD borders, only these fully imbedded in SV intervals are counted as affected, whereas for other structural elements ≥ 1 bp overlaps are counted. Error bars represent SD. **g** Enrichment/depletion of genomic structural elements with the 1000 Genomes Project SNPs (ALL 1kGP), all GWAS SNPs (ALL GWAS), GWAS SNPs associated with hematological parameters (HP), and with autoimmune diseases (AI). Error bars represent SD

CTCF ChIP-seq data confirms that there are SVs which result in altered activity of reference interaction anchors. As an example, deletion chr14:35605439-35615196 of an interaction anchor leads to a significant depletion of CTCF signal in heterozygous samples and even to a complete vanishing of the signal in a homozygous sample (Fig. 4a). The CTCF signal drop reflects the lower or no potential of CTCF to bind to this segment. Therefore, in a cell line exhibiting the deletion, all of the chromatin contacts formed by this locus would not be present in one or both of the homologous chromosomes, depending on the genotype (Fig. 4b). The deletion is located in an intron of gene KIAA0391 but does not excise any coding sequence. Nevertheless, the genotypes show statistically significant differences in transcription rates of several genes (Fig. 4c). Even though the landscape of functional elements around the affected genes is complex to the extent that refrains from drawing definite conclusions, certain explanations may be proposed based on the changes of interaction patterns reflected in 3D models (Fig. 4d) and direct design of experiments. First, deletion chr14:35605439-35615196 removes a CTCF-mediated interaction anchor, which could be involved in the formation of insulated neighborhoods separating the PPP2R3C gene (upstream) from a group of enhancers (downstream). The loss of the putative insulated neighborhood boundary would promote higher activation of PPP2R3C (Fig. 4c), by allowing interactions between the gene and the enhancers. H3K4me1 signal, primarily associated with active enhancers [29], is notably stronger in deletion-affected homozygous GM18526 than in non-affected homozygous GM12878 in the enhancer region of interest (Fig. 4b and Additional file 2: Figure S7). This supports the proposed mechanism underpinning PPP2R3C increased activation. Moreover, the 3D distance between the PPP2R3C promoter and the strongest enhancer from the insulated neighborhood significantly decreases after introducing deletion in the 3D models (Fig. 4e) (see the “Methods” section). The existence of insulated neighborhoods is well established in the literature [11, 30, 31]. Second, deletion chr14:35605439-35615196 removes chromatin contact bringing the NFKBIA gene and one of the enhancers together in 3D space (Fig. 4d). This is reflected by the 3D distances between those two (Fig. 4e). The loss of the contact could explain lower NFKBIA expression in the samples carrying it (Fig. 4c). The association between NFKBIA transcription and genotype is not obvious as we did not find the difference in transcription between genotypes 0|0 and 1|1 statistically significant. However, we suspect that it would occur significant if the genotype 1|1 was represented by more samples than only 14. The deletion causes complex spatial rearrangements also around other genes, which contribute probably to the differences in their transcription rates between samples of different genotypes.

On the other hand, duplications of CTCF-mediated interacting genomic segments result in distinctively high relative values of CTCF signal in those segments in affected samples (Additional file 2: Figure S8A and S8B). The signal enrichment caused by those duplications supports a hypothesis that they create additional CTCF-binding loci with the potential to form additional long-range genomic contacts in the affected genomes.

Inversions in CTCF binding sites also modulate CTCF signal (Additional file 2: Figure S9A), which indicates they introduce changes in chromatin looping.

Apart from SVs disrupting the long-range chromatin interactions that join genomic segments located within one topological domain, there are examples of SVs modifying domain boundaries (Additional file 2: Figure S10A and S11A).

An aggregate analysis (see the “Methods” section) shows that CTCF ChIP-seq signals from samples having deletions (duplications) which intersect CTCF interacting anchors are depleted (enriched) in those anchors compared to signals from samples with the reference genotype (Additional file 2: Figure S12).

Genotypes defined by such SVs exhibit significant differences in the expression of particular genes (Fig. 4c, Additional file 2: Figure S8C, S9B, S10D, and S11E).

We analyze the 3D structures of a part of those loci in Additional file 2: Figure S10B and S10C, Additional file 2: Figure S11C and S11D, and Additional file 2: Figure S13D.

To statistically assess the impact of SVs on the spatial organization of the genome, we analyzed their positions in relation to genomic structural elements like anchors of PET clusters, borders of CCDs, or gaps between them.

We observe that anchors of CTCF PET clusters are depleted of SVs (Fig. 4f) and that the rate of depletion is consistent among the loops of different directionality (Additional file 2: Figure S14).

We further identified CTCF-mediated interaction anchors intersected with enhancers and active and inactive gene promoters (see the “Methods” section). These anchors have a distinguished potential to play an important role in gene regulation. We observe that enhancers and promoters located in CTCF-mediated interaction anchors are significantly more conserved than the respective functional regions residing outside them (Fig. 4f). This indicates the importance of the genomic architecture mediated by CTCF in proper genome regulation. We additionally examined the conservation of CTCF anchoring sites, which interact with enhancers and gene promoters through RNAPII ChIA-PET contacts, and they also seem to be more conserved than the respective genomic functional elements (Fig. 4f).

Surprisingly, borders of CCDs do not seem to be distinctively well conserved. CTCF binding motifs we identified in CTCF ChIP-seq peaks outside CCD borders are significantly more depleted of SVs than the CTCF motifs indicating borders of CCDs (Fig. 4f). Moreover, CCD borders are enriched with rare insertions and are targets of many duplications (Figs. 2e and 4f). There is also a slight enrichment of rare inversions in CCD borders, but because the set of inversions is small, the result is not statistically significant. However, we hypothesize that inverting the CTCF motifs at the borders of topological domains can be an important mechanism of genome reorganization and regulation. Six out of 786 inversions from the CHGV switch the directionality of CCD borders (Fig. 2e). Inversion chr10:15784798-15802449 is an example of such an event (Additional file 2: Figure S11A). It correlates with the transcription rate of a neighboring gene, VIM (Additional file 2: Figure S11E).

Stronger conservation of CTCF-mediated interaction anchors intersected with and connected to the known enhancers and promoters as compared to the conservation of enhancers and promoters located outside the anchors suggests that mutations of these anchors may lead to serious deregulations of gene transcription and can be related to a disease. To test this hypothesis, we intersected CTCF anchors with SNPs previously associated with disease in GWAS [32]. Having in mind the type of cell examined, we created separate sets of GWAS SNPs associated with hematological parameters and autoimmune diseases. Our analysis indeed shows a significant enrichment of these SNP classes in CTCF anchors intersected with enhancers and active promoters (Fig. 4g). Particularly, the enrichment is high in CTCF anchors being in RNAPII-mediated contact with enhancers and promoters. Both former and latter types of anchors are enriched with all GWAS SNPs. Importantly, enhancers and active promoters located outside the CTCF anchors are notably less enriched with GWAS SNPs than CTCF anchors associated with these functional elements (Fig. 4g). Generally, CTCF anchors and CCD boundaries are enriched with GWAS SNPs (Fig. 4g). Our observations are consistent with the studies using capture Hi-C [33, 34] and additionally highlight the role of CTCF in shaping the network of functionally important genomic contacts.

We investigated particular examples of SNPs associated with autoimmune diseases (rheumatoid arthritis and vitiligo, rs4409785 T/C) and hematological parameters (red blood cell distribution width, rs57565032 G/T) (Additional file 2: Figure S15A and S16A). Both alter strongest CTCF binding motifs in the corresponding interaction anchors. However, their effect on CTCF binding is the opposite: rs4409785 increases the strength of CTCF motif it modifies (Additional file 2: Figure S15B), rs57565032 decreases (Additional file 2: Figure S16B). It is reflected in the CTCF signals corresponding to different genotypes (Additional file 2: Figure S15A and S16A). No other SNPs affect the CTCF motifs in those interaction anchors in presented genomes. Samples genotyped by these SNPs demonstrate significant differences in transcription rates of particular genes (Additional file 2: Figure S15C and S16C). One of them, MAML2, has been associated with cancer traits.

The already presented SNP rs12936231 (Fig. 2a) has been reported as a high-risk allele for asthma and autoimmune diseases and suggested to cause chromatin remodeling and alter transcription of certain genes, including ZPBP2, GSDMB, and ORMDL3 [35]. We also found a correlation of genotypes set by rs12936231 with transcription rates of ZPBP2, GSDMB, and ORMDL3 (Fig. 2c). Gene IKZF3, which also exhibits a correlation with rs12936231, has been related to B cell chronic lymphocytic leukemia.

Examples of SNPs in interacting anchors, but not associated with disease so far, can also be found. SNP rs60205880 alters CTCF-mediated chromatin looping and transcription of certain genes (Additional file 2: Figure S2). One of them, CCDC19, has been associated with bilirubin levels; another, IGSF8, is a member of an immunoglobulin superfamily. This demonstrates the potential of investigating genetic variants which target genomic structural elements for the identification of the mechanisms relating them to a disease.

The important question is how large the structural variation among healthy individuals is. Individual genomes sequenced in the 1000 Genome Project carry from 2571 to 6301 SVs, which affect from 1024 to 1419 CCDs and 55–347 CTCF anchors (Additional file 2: Figure S17). Almost all CCDs (98%) have an overlap with at least one SV from the CHGV. However, serious changes in local genome architecture are introduced by disruptions of interaction anchors rather than modifications of genomic regions between them. We identified 4944 unique patterns of SVs altering the interaction anchors in CCDs (we treat 2 patterns as identical if anchor-intersecting SVs they contain are the same; patterns are limited to single CCDs). Together with the 2267 reference CCDs, it gives the number of CTCF-mediated topologies of genomic domains occurring in the 1000 Genomes Project population. We note that types of SVs are well separated in those patterns (Additional file 2: Figure S18). Eighty-seven percent of the patterns are comprised of only one SV type. There are 1539 patterns consisting of 2 or more SVs, and in 902 (59%) of them, all SVs are of the same type.

### Population-specific topological alterations affected by structural variations

We additionally analyzed the intersections of SVs with genomic structural elements in the context of five continental groups: Africa (AFR), the Americas (AMR), East Asia (EAS), Europe (EUR), and South Asia (SAS). These populations are defined by the 1000 Genomes Project [36] (Additional file 1: Table S2).

**Fig. 5:**
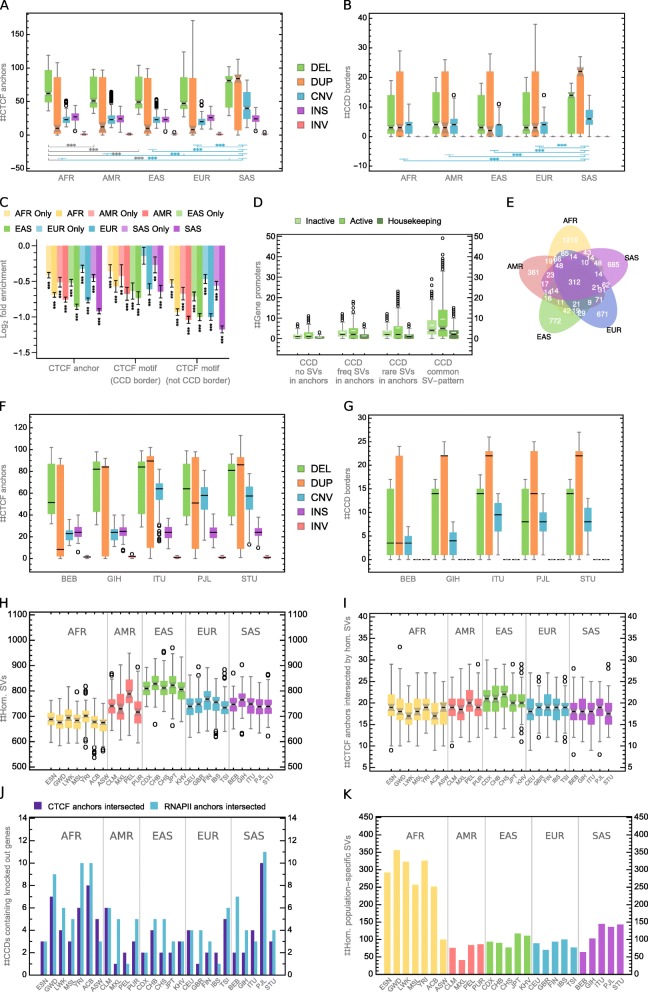
Impact of population-specific structural variants on genome organization. **a** Number of CTCF anchors intersected by SVs of a given type identified in individuals from 5 continental groups, see Fig. 2c for box plot description. **b** Number of domain borders fully overlapped by SVs of a given type identified in individuals from 5 continental groups. **c** Enrichment/depletion of CTCF anchors and CCD boundaries with SVs divided by continental groups. CTCF motifs at CCD borders and outside CCD borders are shown for comparison. Only SVs fully covering motifs are counted as hits. **d** Number of gene promoters in domains covering regions in which SVs are identified. **e** CCD topology variability patterns by continental groups. **f** Number of CTCF anchors intersected by SVs of a given type identified in individuals from South Asian continental group. **g** Number of domain borders fully overlapped by SVs of a given type identified in individuals from South Asian continental group. **h** Number of homozygous SVs in individual human genomes by population. CNVs are treated as homozygous when the number of copies on both homologous chromosomes is different than in the reference (hom., homozygous). **i** Number of CTCF anchors intersected by homozygous SVs in individual genomes by population. **j** Number of CCDs containing human knockouts with CTCF (purple) or RNAPII (cyan) anchors intersected by homozygous population-specific SVs. **k** Homozygous SVs identified in a single human population

In all the populations, deletions of interacting anchors are more frequent than duplications (Fig. 5a). This is not true for CCD borders (Fig. 5b), which agrees with the previous analyses showing that CCD borders are enriched with duplications (we note that there are significantly more deletions than duplications in the set of detected SVs (Fig. 2e)). Alterations of topological domain boundaries can be a general mechanism of genome structure evolution. The above results suggest that such a generic mechanism—similarly to the evolutionary process of introducing gene alterations by duplications—could use redundancy as a security measure. It could leave one chromatin loop with the original transcriptional function under evolutionary pressure, whereas the second could be acquiring novel local spatial landscape for genes and regulatory elements. This is in line with previous research on duplications [37, 38].

Our analysis shows that individual genomes from populations of African ancestry have the largest number of deletions in CTCF interaction sites (Fig. 5a). This is partly due to an outstanding number of all deletions identified in those genomes (Additional file 2: Figure S19). However, we still observe that African genomes, together with European genomes, have CTCF anchor sites less depleted of SVs unique to populations than genomes of other ancestries (Fig. 5c).

Interestingly, SVs found only in European genomes are significantly less depleted in CTCF interaction anchors intersected with enhancers or gene promoters than SVs unique to the rest of 5 distinguished continental groups (Additional file 2: Figure S20). We observe the same effect for borders of CCDs (Fig. 5c), but not for enhancers and promoters residing outside CTCF anchors (Additional file 2: Figure S21). This may suggest that part of the SVs identified in non-European populations overlap interaction sites specific for these populations and not observed in the reference 3D genome.

South Asian genomes, on the other hand, have a distinctively large number of duplicated CTCF anchor (Fig. 5a) and CCD border (Fig. 5b) sites. Whereas the distinctive number of altered structural elements in genomes of African ancestry could be expected based on the large genomic sequence variability in this population reported earlier [36], high structural variability in populations of South Asia is surprising. The ethnic groups which raise the statistics for South Asian continental group, especially those related to CNVs, are Indian Telugu in the UK (ITU), Punjabi in Lahore, Pakistan (PJL), and Sri Lankan Tamil in the UK (STU) (Fig. 5f, g). As a comparison, corresponding statistics for African and European continental groups seem to be more stable across the ethnic groups (Additional file 2: Figure S22). To investigate this further, we analyzed homozygous SVs. We hypothesized that the elevated number of structural changes observed in South Asian genomes could be caused by the high number of homozygous SVs that some of the populations in this continental group exhibit due to high consanguinity rates [39].

There are 13,767 homozygous SVs in the CHGV (we treat a CNV as homozygous, when there is a non-reference copy number on both homologous chromosomes). According to the data, genomes from East Asia, not South Asia, carry the largest number of the homozygous SVs (Fig. 5h). However, the differences in homozygous sequence variation are not reflected in the number of homozygously altered CTCF anchors. The latter seems not to be changing across populations (Fig. 5i).

The fruitful study of natural human knockouts performed on a cohort of 10,503 Pakistanis by the Human Knockout Project [40] made us investigate the homozygous SVs from the CHGV identified uniquely in a single population. We took the 1317 knocked out genes found in individuals from South Asia (in majority belonging to Urdu and Punjabi ethnic groups, over 70%) [40] and considered 656 CCDs they were located in. It turns out that homozygous SVs identified uniquely in Punjabi population intersect CTCF and RNAPII anchors in the largest number of CCDs (10 and 11 respectively) containing the gene knockouts (Fig. 5j), even though a moderate number of population-specific homozygous SVs was found for this group (Fig. 5k). This suggests that gene knockouts may be accompanied (preceded, followed or assisted) by homozygous structural rearrangements.

For each of the continental groups, we prepared a list of patterns (similar to those described in the previous section) of anchor-intersecting SVs, which alter CCDs in the individuals belonging to this group. Even though most of the patterns are population-specific, we found 312 (6%) patterns common for all 5 continental groups (Fig. 5e). CCDs in which we found the common SV patterns are characterized by a particularly high number of gene promoters, including promoters of housekeeping genes (Fig. 5d). There are statistically more gene promoters in those CCDs than in other CCDs with modified anchors and in domains covering segments without changes in CTCF anchor sites. It is worth noticing that more promoters are located in CCDs containing CTCF anchors under variation than in those without them, which may suggest that the architecture of transcriptionally active genomic regions is more prone to mutation. CCDs with CTCF anchors under rare variation contain statistically more promoters of active genes than CCDs with CTCF anchors affected only by frequent SVs (Fig. 5d). Moreover, rare SVs happen to affect CTCF anchors in domains containing outstanding number of promoters (Fig. 5d). CCDs with rare variants in CTCF-interacting anchors can have up to 96 promoters of active genes (compared to 39 in CCDs with frequent SVs in anchors).

### Regulation of gene transcription altered by topological variations in population

By combining information on chromatin interactions and population-scale genetic variation with transcriptome data from 462 lymphoblastoid cell lines gathered by the gEUVADIS Consortium [41, 42], we were able to draw first to our knowledge population-scale evidence-supported conclusions on the functional relation between SVs and genome architecture and provide a deeper insight into the functional role of genetic variation in the human genome. The results of our analysis indicate that SVs influence gene transcription primarily by rearranging local looping structure of the genome.

For 445 out of the 462 samples provided by the gEUVADIS Consortium, there are also genotypes available in the 1000 Genomes Project database. We thus used PEER-normalized [41] gene expression levels of these 445 individuals for the association with their genotypes to identify expression quantitative trait loci (eQTLs). We use the term eQTL for any variation of genomic sequence which is identified as having an effect on the gene transcription level. The eQTL analysis was performed only with SVs, excluding SNPs.

We performed principal component analysis (PCA) on the expression data, which pinpointed 14,853 genes having the biggest variation in expression rates among individuals from all 23,722 genes present in the gEUVADIS dataset. We then related the expression levels of each of the 14,853 genes to genotypes (see the “Methods” section).

In the studies on eQTLs published so far [6, 41, 43–46], a genomic region of arbitrary size around a gene in question was conventionally set, and only the genetic variants located within this linear region were tested for being eQTLs for this gene. We argue that a more natural approach is to look for eQTLs within the whole topological domain the gene is located in. Therefore, for each of the selected genes, we evaluated the associations of its expression levels with all the genotyped SVs residing in the same CCD. For every gene-SV pair, least-square linear regression was performed and the significance of the slope was then tested in the permutation test. The resulting *p* values were adjusted for multiple testing to control the false discovery rate (FDR). We set a threshold of acceptance for FDR ≤ 10% (see the “Methods” section).

**Fig. 6:**
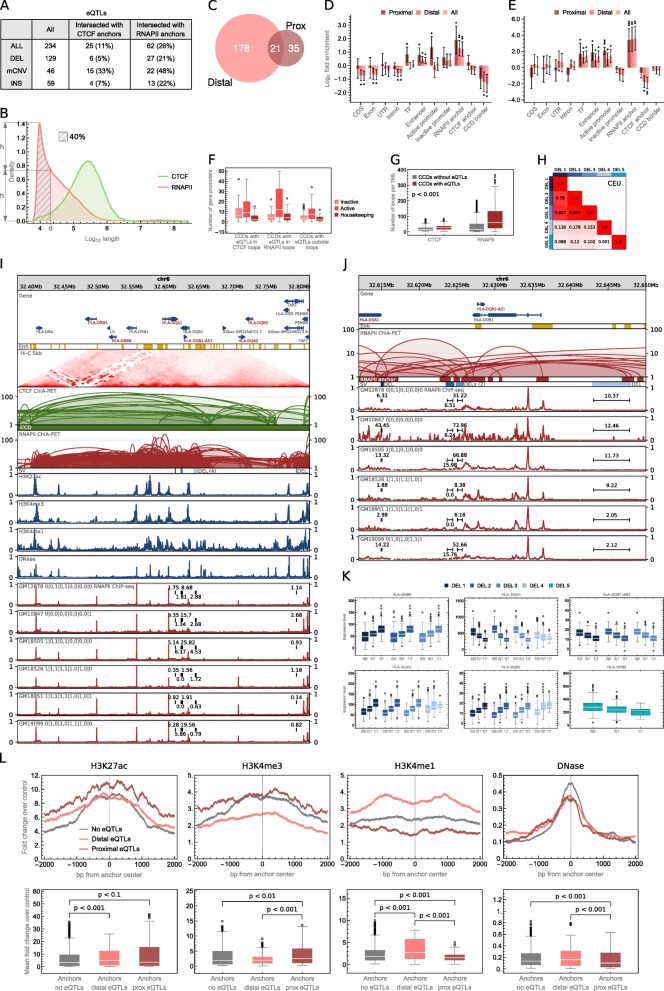
Role of chromatin rearrangements in the regulation of gene transcription. **a** Table summarizing identified eQTLs and their intersections with interaction anchors. **b** Density plot showing genomic span distribution of PET clusters. *d* is the value (17,800 bp) by which eQTLs were split into proximal and distal*.* **c** Venn diagram showing the number of proximal (Prox) and distal eQTLs. **d** Enrichment/depletion of genomic elements with eQTLs. Error bars represent SD. **e** Enrichment/depletion of genomic elements with eQTLs of housekeeping genes. Error bars represent SD. **f** Abundance of gene promoters in CCDs, in which eQTLs were identified, see Fig. 2c for box plot description. *n* = 16 (CCDs with eQTLs in CTCF loops), 32 (CCDs with eQTLs in RNAPII loops), and 106 (CCDs with eQTLs outside loops) sample points. **g** Distributions of chromatin loop density in CCDs in which eQTLs were identified and in other CCDs. The density is measured for a particular CCD as an average number of CTCF-/RNAPII-mediated chromatin loops covering a 1-Mb fragment of this CCD. Differences between the groups are significant (*p* values < 0.001), see Fig. 2c for box plot description. *n* = 2125 (CCDs without eQTLs) and 142 (CCDs with eQTLs) sample points. **h** Linkage disequilibrium (measured as *r*^2^ value in the CEU population) between deletions shown in **i** and **j**. Colors are assigned to the deletions as in **i**–**k**. **i** Browser view of a 0.4-Mb genomic segment with 5 deletions identified in a part of the human population, which disrupt RNAPII anchors and are eQTLs for 6 neighboring genes (signed with the red font). Each deletion has its color. RNAPII ChIP-seq signals from 6 lymphoblastoid cells of different genotypes are presented for comparison. For each track, normalized ChIP-seq signal values were divided by the maximal value of the signal in the visualized region. Sum of the signal values over the genomic regions occupied by the deletions is marked in each signal track. H3K27ac, H3K4me3, H3K4me1, and DNase-seq signal tracks from GM12878 are shown. **j** Close-up on the RNAPII-mediated interactions affected by 4 of the 5 deletions. Only the loops affected by the deletions are shown for clarity. **k** Genes which transcription is correlated with one or more of the deletions shown in **i** and **j** (*p* value < 0.001). Boxes with transcription rates associated with a particular deletion are marked with the color assigned to the deletion, as in **i** and **j**, see Fig. 2c for box plot description. *n* = 132, 167, 146 (DEL 1); 91, 208, 146 (DEL 2); 91, 208, 146 (DEL 3); 257, 158, 30 (DEL 4); 265, 154, 26 (DEL 5) sample points. **l** Signal strength of histone marks and DNase hypersensitivity sites in interaction anchors intersected with proximal eQTLs, distal eQTLs, and not intersected by eQTLs. For each mark, two plots are presented. A signal track around anchor center (± 2 kb) showing values for each genomic position averaged over all anchors from a given group (top). A box plot showing mean signal values in the same regions (bottom). Original signal values represent fold change over control. CTCF and RNAPII anchors were analyzed jointly, see Fig. 2c for box plot description. *n* = 1000 (anchors no eQTLs), 523 (anchors distal eQTLs), and 242 (anchors prox eQTLs) sample points

We identified 234 unique SV-eQTLs modifying expression levels of 192 genes. The majority of the eQTLs found (55%) are deletions (Fig. 6a).

Earlier studies on eQTLs were limited in exploring the causal relation between genetic variation and gene expression to analyzing gene-variant and exon-variant intersections [43, 45], or the influence of genetic variation on transcription factor binding sites (TFBSs), transcription start sites (TSSs), or transcription end sites (TESs) [41, 46, 47]. In particular, one of the latest to our knowledge big study on the impact of structural variation on human gene expression reported that over 88% of predicted causal SVs did not alter gene structure or dosage [22]. The study showed enrichment of causal non-coding SVs in regions occupied by transcription factors or surrounding genes at distances up to 10 kb, but no deepened analysis of these regions was performed. Our analysis gives a broader idea of this relation and sheds light on the mechanisms through which SVs take part in genome regulation.

In agreement with [22, 46, 47], we observe an enrichment of eQTLs in TFBSs, but we see significantly higher enrichment of these in the genomic regions responsible for chromatin spatial organization. We divided the identified eQTLs into two sets: those located on the DNA chain closer than 17,800 bp to the genes they modify (proximal) and those located further apart (distal) (see the “Methods” section). The splitting value of 17,800 bp was chosen based on the distribution of RNAPII PET clusters lengths. It is a value for which the density of lengths of RNAPII clusters is equal to the half of the maximum density (Fig. 6b). The division is not exclusive—some of the eQTLs correlated with more than one gene are distal for one of them and proximal for other (Fig. 6c).

Active promoters and TFBSs are enriched with proximal eQTLs demonstrating their importance as gene-adjacent regulatory sites. Enhancers apart from being enriched with proximal eQTLs are enriched with the distal ones and represent regulatory elements interacting with genes by the nuclear space. However, the genomic elements most enriched with both proximal and distal eQTLs are anchors of RNAPII PET clusters (Fig. 6d). The abundance of eQTLs in the anchoring regions of the strong chromatin interactions mediated by RNAPII reaffirms the crucial role of this element of genome architecture in gene regulation.

As an example of eQTLs altering chromatin looping mediated by RNAPII, we investigate 5 deletions located in a HLA region (Fig. 6i). All of these deletions affect RNAPII interacting anchors (Fig. 6j) and are correlated with one or more of 5 HLA genes neighboring them (Fig. 6k). Three of the deletions are in a very strong linkage disequilibrium (LD) with each other (tested on the Central European population, Fig. 6h).

We hypothesize that proximal eQTLs modify TFBSs, TSSs, and TESs of genes as well as gene sequences but mostly they alter genes’ spatial contacts with regulatory elements and possibly with interaction centers, which has an immediate and straightforward influence on genes’ expression levels. Distal eQTLs have in turn higher potential to, apart from altering long-range RNAPII interactions, disrupt CTCF interactions that are longer than RNAPII-mediated chromatin loops (Fig. 6b) and shape the spatial structures of the whole topological domains.

Deletion chr1:248849861-248850138 is one of the eQTLs intersecting CTCF-mediated interaction anchors (Fig. 6a). Together with two other deletions (all in a very strong LD (Additional file 2: Figure S13F)), it introduces architectural changes correlated with increased transcription of a group of at least 6 olfactory receptor family genes, all residing on one chromatin loop (Additional file 2: Figure S13C). 3D models give more insight into the topological alterations induced by the deletions (Additional file 2: Figure S13D).

Another example of identified eQTL which alters CTCF-mediated chromatin structure is duplication chr17:44341412-44366497 (Additional file 2: Figure S23A). It duplicates the border of a CCD, and its emergence correlates with the transcription of the KANSL1-AS1 gene (Additional file 2: Figure S23B).

Even though we observed examples, we did not find anchors of CTCF PET clusters to be enriched with distal eQTLs (Fig. 6d). The fact we note, however, is that 17 of the identified eQTLs (24% of the anchor-intersecting eQTLs) intersect both RNAPII and CTCF anchors (Fig. 6a) and 36 (58%) of the eQTLs intersecting RNAPII anchors were detected in CCDs in which eQTLs targeting CTCF anchors were also found. This suggests that a change in gene expression observed among individuals can often be a result of a coordinated modification of RNAPII and CTCF anchors, but more investigation is needed to confirm this claim. Interestingly, CCDs in which eQTLs alter RNAPII anchors tend to embrace more active genes and housekeeping genes than CCDs with eQTLs not overlapping any interacting segments (Fig. 6f). On the other hand, CCDs with eQTLs in CTCF anchors contain many inactive genes (Fig. 6f).

Furthermore, we suspect that the enrichment analysis does not indicate that alterations of CTCF anchors significantly contribute to the variation of gene expression in population because the disruption of CTCF chromatin contacts would often provoke drastic changes in the local spatial organization of a genome not observed in healthy people. As we showed earlier, SNPs associated with disease favorably emerge in CTCF anchors (Fig. 4g).

For comparison with capture Hi-C (CHi-C) data, we mapped the identified eQTLs on genomic interactions reported in Mifsud et al. [33, 48]. Anchors of promoter-promoter and promoter-other CHi-C interactions were analyzed for the enrichment with the eQTLs (Additional file 2: Figure S24). The analysis shows that CHi-C anchors containing promoters are enriched with proximal eQTLs and depleted of the distal ones. A similar effect can be observed for ChIA-PET RNAPII anchors intersected with promoters (Additional file 2: Figure S24). However, unlike CHi-C anchors containing enhancers, ChIA-PET anchors intersected with enhancers are significantly enriched with distal eQTLs. The results for ChIA-PET data highlight the role of distal enhancers in gene regulation and may suggest that the interactions identified in RNAPII ChIA-PET are more transcriptionally active than the ones reported from CHi-C.

To state more firmly the relationship between proximal and distal eQTLs and chromatin activity, we collected (see the “Methods” section) sequencing (ChIP-seq) data for three histone modifications (H3K27ac, H3K4me3, H3K4me1) and information on chromatin accessibility (DNase-seq) and analyzed it in interaction anchors intersected with the eQTLs. H3K4me3 is primarily associated with promoters, H3K4me1 with active enhancers, and H3K27ac with active promoters and enhancers [29]. As expected, the interaction anchors altered by proximal eQTLs are enriched with promoter signal, whereas those affected by distal eQTLs with enhancer signal (Fig. 6l). This confirms that proximal eQTLs disrupt promoter-enhancer communication at the site of the promoter and distal eQTLs at the site of the enhancer. The results are statistically significant, even though interaction anchors are enriched with chromatin marks in general (Fig. 6l) [17]. Furthermore, eQTLs emerge in densely connected genomic regions (Fig. 6g, l). This is also reflected by the fact that a single eQTL often intersects more than one RNAPII interaction anchor (Fig. 6j).

We repeated the eQTL analysis described above for housekeeping genes only (selected based on Eisenberg and Levanon [49]) to see if we find eQTLs for them and where the potential eQTLs are localized (see the “Methods” section). We found 36 unique eQTLs for 33 different housekeeping genes. None of the eQTLs is located within CTCF anchor, but we observe significant enrichment of them in RNAPII anchors (Fig. 6e). Therefore, there are differences in the expression rates of housekeeping genes among the samples, and they are mainly correlated with alternations of long-range chromatin contacts mediated by RNAPII.

On the other hand, we separately analyzed immune-related genes as genes specific to the lymphoblastoid cell lines (see the “Methods” section). Fourteen eQTLs were identified for these genes, out of which 4 intersect CTCF anchors. Three of these are anchors which contain enhancers and 1 contains a promoter region.

Two of the immune-related eQTLs (deletion chr22:39357694-39388574 and CNV chr22:39359355-39379392) cover the same CTCF anchor (Additional file 2: Figure S25A). Both are eQTLs for genes APOBEC3A, APOBEC3B, and CTA-150C2.16 (Additional file 2: Figure S25B), but the deletion completely excises APOBEC3B gene. In the presented samples (Additional file 2: Figure S25A), both of the SVs were identified, meaning that locus chr22:39357694-39388574 is (haplotype-specifically) excised in those genomes, which is reflected in CTCF signal for these samples.

Another example of an eQTL altering a CTCF anchor and regulating an immune-related gene is deletion chr17:73107713-73108273 (Additional file 2: Figure S26A). It is located over 750 kb apart from the correlated gene TRIM47 (Additional file 2: Figure S26B).

Whether cell type-specific genes are distinguished targets for SVs altering core chromatin architecture (as CTCF is believed to form the backbone network of genomic interactions) is an interesting question. However, more extensive testing has to be done to explore this hypothesis.

## Discussion

It is already well established that part of the transcriptional variation between genomes or pathogenic phenotypes can be caused by chromatin topological alterations, but we still lack extensive genome-wide testing to assess the abundance and importance of these events. Our understanding of the importance of chromatin architecture in genome regulation is based mainly on particular cases of SVs disrupting local 3D chromatin structure and subsequently leading to the deregulation of transcription of particular genes (in most of the studied cases associated with disease) [7, 9, 11]. There were more general insights into chromatin spatial rearrangements, but only in cancer genomes and at a less detailed level of the whole topological domains and their boundaries [8, 10]. No attempt was made so far to assess the abundance of chromatin architecture alterations in normal genomes, their functional impact, and the frequency with which genetic variations (related and not related with pathogenic phenotypes) target genomic regions responsible for the proper chromatin folding. The latter is specifically intriguing in the context of genome-wide association studies showing that over 95% of identified SNPs are located outside coding sequences [5]. This study is the first such attempt.

We mapped genetic variants identified in individuals from 26 human populations in the 1000 Genomes Project and disease-associated SNPs from GWAS onto chromatin three-dimensional structure of human lymphoblastoid cell line represented by CTCF and RNAPII ChIA-PET data. Our strategy for analyzing high-resolution CTCF and RNAPII genomic interaction data gives a comprehensive insight into the impact of SVs on chromatin organization. In agreement with previous studies [15, 50, 51], the analysis shows a high conservation of CTCF/cohesin-mediated chromatin topology between individual genomes. Moreover, the CTCF/cohesin-mediated promoter-enhancer interactions resulted as more conserved than enhancers and gene promoters not forming these type of interactions (Fig. 4f). This and the enrichment of disease-associated SNPs in CTCF/cohesin interaction anchors (Fig. 4g) indicate that they are critical mutational targets and that a significant part of non-coding regions of the genome targeted by disease-associated genetic variations is responsible for chromatin organization in cell nuclear space. On the other hand, alterations of chromatin interaction networks mediated by RNAPII are closely associated with the variation of gene transcription among population (Fig. 6d). The analysis of histone marks in RNAPII-mediated interaction segments targeted by transcription-associated SVs confirms that these SVs disrupt promoter-enhancer contacts (Fig. 6l). We found SVs correlated with gene transcription rates and disrupting local chromatin architecture built by RNAPII in a critical HLA region (Fig. 6i–k). We observe cases in which both CTCF and RNAPII anchors are modified in a topological domain containing genes with altered transcription rates, but the overall analysis indicates that the chromatin structure built by CTCF is strongly conserved across individuals and variation in gene transcription occurs by modifications of RNAPII interactions formed within this structure. This hypothesis is consistent with the model presented by Tang et al. [17]. However, we suspect that the fact that we did not identify many examples of eQTLs located in CTCF anchors may also mean that the linear model used to detect eQTLs is too simple to account for complex nonlinear changes in gene transcription caused by modification of CTCF-mediated chromatin looping. This requires further investigation. Intriguingly, the evidence provided in this study identifies CTCF binding sites involved in the insulation of topological domains as frequently affected by duplications. This suggests that duplications of domain boundaries can have a distinguished role in evolutionary adaptation, similarly to duplications of genome coding sequences [37, 38].

We identified African and South Asian genomes as exhibiting the highest rates of structural variation in genomic interaction anchors and topological domain boundaries (Fig. 5a, b). South Asian genomes further stand out from the rest by having distinctively high numbers of mCNVs occurring in these genomic elements. This statistic is high within the continental group of South Asia mainly due to the input of three ethnic groups: Indian Telugu in the UK (ITU), Punjabi in Lahore, Pakistan (PJL) and Sri Lankan Tamil in the UK (STU) (Fig. 5f, g). We attempted to link the high rates of genome topology-affecting SVs observed in South Asia continental group to high consanguinity rates exhibited by some of the populations in this group. However, we did not detect an association between those two. A further investigation is needed including chromatin conformation capture experiments for different ethnic groups to address this question. On the other hand, we do note that SVs unique for the PJL ethnic group target genomic interaction anchors in the highest number of topological domains carrying knocked out genes found in the Human Knockout Project from all the populations sequenced in the 1000 Genomes Project (Fig. 5j). This suggests that gene knockouts may be accompanied (preceded, followed, or assisted) by homozygous structural rearrangements. Interestingly, we observe that the rate of depletion of population-specific SVs in structural elements of the reference 3D genome is the smallest for European genomes (Fig. 5c and Additional file 2: Figure S20). Given that the 3D genome we use as the reference was obtained from a sample of European ancestry, it may suggest that part of the SVs specific for genomes of other ancestry target genomic interactions unique for those populations and not represented by the reference. This is an argument for more diversity in generating 3D genome data. Interestingly, domains which topology is affected by SV patterns occurring in genomes from all continental groups carry a distinctively high number of genes, including housekeeping genes (Fig. 5d). We generally observe that domains in which we identified SVs targeting CTCF/cohesin-mediated interaction anchors carry significantly more genes than domains in which SVs occur only outside the anchors. These results may suggest that the replication process exhibits specificity in gene-rich genomic regions which causes common faults in copying sequences around genomic interaction sites which could be involved in this process. Another explanation could be that there is a set of topology-affecting SVs which occurred in gene-rich domains early in the evolution. The latter, however, does not explain the high abundance of genes in domains affected by rare SVs targeting CTCF/cohesin-mediated interaction anchors (Fig. 5d).

The analysis of spatially interacting genomic segments was possible using data from ChIA-PET experiments which identify such segments with high accuracy genome-wide. We applied additional filtering on CTCF ChIA-PET interacting segments, checking them for the co-occupancy by CTCF and cohesin ChIP-seq peaks, to obtain a highly credible set of CTCF-mediated chromatin interactions supported by cohesin. In case of CTCF ChIA-PET, it is even possible to identify individual CTCF binding motifs involved in the formation of the interactions, which brings high precision to the analysis. Such analysis would not be possible using Hi-C data in case of which genomic interaction segments are demarcated artificially, by segmenting the genome into adjacent bins of equal size and which resolution is rarely under the order of tens of kilobases, as a high resolution is obtained at the cost of very deep genome sequencing. The limitation of ChIA-PET experiment is that it captures chromatin interactions mediated by a particular protein, in contrast to Hi-C which identifies interactions of all kinds. Thus, Hi-C is a more complete representation of chromatin contacts present in the cell nucleus. However, CTCF ChIA-PET detects structural features exhibited by the non-specific Hi-C data. Data from these sources are highly correlated at the global whole-chromosome scale (Spearman’s correlation coefficient in the range of 0.7–0.9) [17] and identify a very similar landscape of genomic structures at the local scale of topological domains (Fig. 1b) and chromatin loops (Fig. 1c). Moreover, the distinction between CTCF- and RNAPII-mediated interactions enabled us to spot the differences in the impact of SVs on them.

We used ChIA-PET data for GM12878 cell and treated it as a reference 3D genome for mapping SVs from other lymphoblastoid cell lines. CTCF and RNAPII ChIA-PET datasets for GM12878 are of the highest quality, and to our knowledge, no such datasets are currently available for any other lymphoblastoid cell line. Based on these highly credible genomic interactions and information on SVs identified in a population of 2504 human lymphoblastoid cells, we computationally predict chromatin interaction patterns for those cells. We claim that our computational tool is very useful for obtaining individualized chromatin interaction patterns and in silico models of chromatin structures in the absence of experimental data, which generation requires expertise and certain money investments. On the other hand, because of the data unavailability, we could not confront our predictions with experimentally generated genomic interaction maps. No new biological experiments were conducted to support the correctness of our predictions, as this is purely computational analysis. However, we presented examples supported by available ChIP-seq datasets (Figs. 2a, 4a, and 6j; Additional file 2: Figure S2, S8, S9, S10, S11, S13, S15, S16, S23, S25, and S26) and genome-wide ChIP-seq analyses (Fig. 2d and Additional file 2: Figure S3 and S12) showing that predicting chromatin interactions based on ChIA-PET data for GM12878 and SVs from other lymphoblastoid cells is reasonable. Furthermore, we presented 3D models of an extensively studied (including the execution of CRISPR/Cas9 experiments) genomic region showing that their features are perfectly in line with discoveries and claims reported on this region [11] and that they could serve as accurate models for the mechanisms described in the earlier study. We do not claim that the models generated with our modeling method can alone explain the mechanisms underpinning associations of some SVs with gene transcription or constitute a proof of such mechanisms actually being the cause of observed changes in gene transcription rates. However, we believe that they can be a supporting tool in the analysis of potential disruptive effects of studied SVs on chromatin spatial organization and functional consequences of these alterations, helping to design a comprehensive study and to plan experiments more strategically.

We show that the topological variability of the human genome is rather limited (Additional file 2: Figure S18). However, because of the data used, the predictions are rather confined to lymphoblastoid cell lines. Nonetheless, our modeling method and web service providing the modeling tool can operate on uploaded data, if such data is at user’s disposal.

## Conclusions

This is the first genome-wide study on the influence of genetic variants on the chromatin organization and topological variability in the human population. It shows the critical impact of genetic variants on the higher-order organization of chromatin folding and provides a unique insight into the mechanisms regulating gene transcription at the population scale, among which the local arrangement of chromatin loops seems to be the leading one. This study highlights the importance and reason for further study on the role of chromatin architecture in genome regulation. It shows that further work on computational prediction of the chromatin 3D structures based on different factors changing among individuals is required, as the emerging evidence shows that chromatin spatial organization is a crucial element to understand the genome regulation.

## Methods

### Genomic interactions

Genomic interactions analyzed in this study are 92,808 CTCF PET clusters and 100,263 RNAPII PET clusters identified by Tang et al. [17] for the GM12878 cell line (Additional file 1: Table S3 and S4) [18]. We refer the reader to this work for details on data processing pipeline used to find these interactions. Briefly, pair-end reads (PETs) sequenced in long-read ChIA-PET experiment were mapped to the human reference genome (hg19). Inter-ligation PETs were selected by the criterion of genomic span between the two ends of a PET exceeding 8 kb. Inter-ligation PETs overlapping at both ends were clustered together creating unique contacts (PET clusters) between 2 specific interaction loci, of strength equal to the size of the cluster. Anchors of CTCF PET clusters located within the distance of 500 bp along the DNA sequence were merged to more accurately correspond to loci covered by single CTCF binding peaks. This step led to further clustering of CTCF PET clusters and reduced their number from 92,808 to 80,157. Individual inter-ligation PET clusters and PET clusters of strength smaller than 4 are referred to as singletons.

### ChIP-seq consensus peaks

We analyzed the directionality of CTCF interactions similarly to Tang et al. [17]. CTCF, SMC3 and RAD21 uniform ChIP-seq peaks available for the GM12878 cell line were downloaded from the ENCODE database (Additional file 1: Table S5) [52]. We extracted the 4-way consensus regions from all 4 sets of CTCF peaks to get highly credible CTCF-binding peaks. The same consensus was performed on SMC3 and RAD21 ChIP-seq segments to identify cohesin-binding peaks. Finally, 25,250 consensus regions from the CTCF and cohesin consensus peaks were obtained.

### CTCF motif identification

We searched the CTCF/cohesin consensus peaks for CTCF-binding motifs. Nucleotide sequence of each of the CTCF/cohesin peaks was extracted from the hg19 assembly (downloaded from the UCSC database) using BEDTools (version 2.26.0) getfasta utility [53] and provided as an input to STORM (CREAD package version 0.84) [54]. Given the position weight matrix of a particular transcription factor-binding motif, STORM predicts the occurrences of the factor-binding motifs in provided DNA sequences. We performed the search with CTCF position weight matrix MA0139.1 downloaded from the JASPAR database [55] and found CTCF-binding motifs in 24,013 out of the 25,250 CTCF/cohesin consensus peaks. Only the motifs having a score higher than 0 were considered as valid, and for each peak, a motif with the highest score was selected.

### Assigning orientation to CTCF loops

The CTCF motifs were overlapped with CTCF PET clusters. Forty-four thousand three hundred eighty out of the 80,157 CTCF PET clusters had both anchors overlapped by CTCF/cohesin consensus peaks (Additional file 1: Table S1), and only 2334 (3%) of them had no intersections with the consensus peaks at neither of sides. For 40,624 out of the 44,380 clusters (92%), at least one CTCF motif was found at both anchors. Thirty-three thousand sixty-two of these had exactly one motif at either side. PET clusters with anchors having more than one CTCF motif and of contradictory orientations were filtered out. From the 37,289 CTCF PET clusters with motifs of unique orientation in both anchors, 24,181 (65%) had motifs of convergent orientation at the two anchors, 6118 (16%) had motifs of tandem right orientation, 6089 (16%) PET clusters were of tandem left orientation, and 901 (2%) were of divergent orientation.

### Chromatin contact domains

In this study, we used 2267 CTCF-mediated chromatin contact domains (CCDs) identified by Tang et al. (Additional file 1: Table S6) [18]. We refer the reader to this work for the details of CCD calling. Briefly, CCDs were identified by searching each chromosome for genomic segments continuously covered with CTCF PET clusters supported by CTCF/cohesin consensus peaks. Each identified CCD starts where the most upstream CTCF anchor from all the anchors of the CTCF PET clusters comprising the CCD starts and ends where the most downstream CTCF anchor ends. To define the borders of the CCDs more accurately, CTCF motifs found in the CTCF/cohesin consensus peaks and positioned within outermost anchors were identified. From these, the outermost CTCF motifs were selected as CCD borders. CTCF/cohesin consensus peaks with CTCF motifs were found in 4346 (96%) out of the 4534 outermost anchors. In case of the remaining 188 anchors, the strongest CTCF motifs identified in the full DNA sequence covered by the anchors were selected as indicators of CCD boundaries. Genomic regions complementary to CCDs (less hg19 reference genome assembly gaps) were defined as CCD gaps.

### Enhancers and promoters

Definitions of enhancers used throughout this study were extracted from ChromHMM [56] hg19 annotations for the GM12878 cell line downloaded from the ENCODE database (Additional file 1: Table S5) [52]. Both weak and strong enhancer annotations were adopted. Promoters were defined as ± 2 kb regions surrounding the gene transcription start sites (TSSs). The TSS coordinates were adopted from the GENCODE release 27 (mapped to hg19) [57, 58]. Only the promoters for protein-coding genes were considered. Promoters were defined as active if overlapped with anchors of RNAPII PET clusters or with RNAPII consensus ChIP-seq peaks and defined as inactive otherwise. RNAPII consensus peaks were obtained by performing consensus on 3 sets of RNAPII uniform ChIP-seq peaks available for the GM12878 cell line in the ENCODE database (Additional file 1: Table S5).

### Enrichment analyses of SVs in genomic elements

In this study, we tested various genomic elements for the enrichment or depletion with structural variants (SVs). These tests were conducted according to a common scenario. Genomic elements of a given type represented by their positions in the hg19 reference genome were intersected with the positions of SVs. The ones having at least 1 bp overlap with at least 1 SV were counted. The genomic elements were then intersected with simulated SVs from 1000 sets generated by randomly shuffling positions of the original SVs, and the null distribution of counts of genomic elements overlapped with SVs was calculated. Each shuffled set contained the same number of elements in total and the same number of elements in subsets (deletions, duplications, etc.) as the real SV set. Elements of these sets were equally distributed on chromosomes and equally distributed in length to the real SVs. The operations of shuffling and intersecting genomic segments were performed with BEDTools (version 2.26.0) [53]. The enrichment or depletion of genomic elements overlapped with the real SVs compared to the same elements overlapped with randomly positioned simulated SVs was expressed as log2 fold change of the number of the former versus the mean of the distribution of the number of the latter. Values of the measure were represented by the height of bars in the plots. Error bars in the plots show standard deviations of log2 fold changes in each permutation test. To estimate the statistical significance of the test results, one-sided *p* values were calculated from the simulated distributions and marked above the bars by stars (3 stars, *p* value < 0.001; 2 stars, *p* value < 0.01; 1 star, *p* value < 0.1).

### Subsets of SVs in enrichment analyses

For individual tests, the set of SVs was divided into various subsets. In particular, SVs with variant allele frequency (VAF) lower than 0.001 were considered separately in certain tests. In others, SVs were grouped according to the ancestry of individuals they were identified in, and sets of SVs emerging uniquely in one subpopulation were also created. The special set of SVs correlated with gene expression (eQTLs) was subdivided into 2 sets: a set of eQTLs located closer on the DNA chain than 17,800 bp apart from the genes they modified and a set of eQTLs located further apart from their genes. The distances were calculated between TSSs (as defined in GENCODE version 12) [58] and centers of eQTL segments.

### Subsets of GWAS SNPs

The set of GWAS SNPs used in this study was derived from the NHGRI-EBI GWAS Catalog, version from January 31, 2018 [32]. SNPs of traits associated with autoimmune diseases and hematological parameters were extracted as separate sets and mapped to dbSNP Build 150 for hg19 human genome assembly. SNPs mapping outside the main chromosome contigs, not having dbSNP ID or without coordinates on the hg19 and records containing multiple SNPs were excluded. This resulted in 2330 and 3919 unique SNPs associated with autoimmune diseases and hematological parameters respectively. For permutation tests with SNPs identified in healthy samples in the 1000 Genomes Project, we extracted a random sample of 1 million elements from the whole set of SNPs to limit the computation time and storage space.

### Genomic elements in enrichment analyses

Analyzed in permutation tests, genomic elements associated with genes (annotated protein-coding sequence regions (CDSs), untranslated regions (UTRs) in protein-coding regions, exons, and introns) were adopted from the GENCODE release 27 (mapped to hg19). Permutation tests with eQTLs were an exception—in this case, gene elements from version 12 of GENCODE were used to maintain the consistency with the expression data which was analyzed with the earlier version of GENCODE. The positions of transcription factor-binding sites (TFBSs) were adopted from a file with uniform TFBS peaks downloaded from ENCODE (Additional file 1: Table S5).

### Analysis of CTCF interaction anchors altered by SNPs

To test the impact of SNPs on the probability of CTCF binding to a CTCF anchor, we searched the nucleotide sequence of the anchor for CTCF motifs and compared the number and scores of these motifs with the CTCF motifs identified in the nucleotide sequence of this anchor after the introduction of alternative alleles. Only the motifs with a score higher than 0 were taken into consideration. Identification of CTCF motifs was performed as described in the “CTCF motif identification” section above.

We used ggseqlogo R package [59] to generate sequence logos from the frequency matrix MA0139.1 downloaded from the JASPAR database.

### mRNA quantifications

PEER-normalized expression levels of 23,722 genes provided by the gEUVADIS Consortium [42] were used in our analyses. We refer the reader to Lappalainen et al. [41] for details on the process of transcriptome quantifications. In short, RNA-seq read counts over genes annotated in GENCODE (version 12) were calculated. This was done by summing all transcript RPKMs per gene. Read counts were corrected for variation in sequencing depth by normalizing to the median number of well-mapped reads among the samples and for technical noise. The latter was removed using PEER [60]. We logarithmized the corrected quantifications and standardized the distributions of transcription rates (for each gene individually).

### Genotypes

Definitions of genomic sequence variations were taken from Sudmant et al. [25]. This SV set is a refined version of the callset released with the 1000 Genomes Project marker paper [36]. Only SVs (deletions, duplications, copy number variants, inversions, and insertions) were considered; SNPs were not included in the analysis. The genotype of an individual was represented as a sum of SV copies present on homologous chromosomes of the individual. Deletions were indicated by negative numbers. For example, if an individual had a deletion on both copies of a chromosome, the genotype was − 2. If it had 2 more copies of a genomic region (in relation to the reference genome) on one chromosome from the pair and 1 additional copy of this region on the second chromosome from the pair, the genotype was 3. Genotypes unchanged compared to the reference sequence (hg19 in this case) had codes 0. Genotypes of abundance lower than 1% in the studied population were neglected.

### Linear models

The gEUVADIS Consortium provides RNA-seq data for 462 samples, but only a subset of these (445 samples) was genotyped in the 1000 Genomes Project. Thus, our analyses were performed on a population of 445 individuals for which both transcription and genotype data were available. Only the genotypes of abundance higher than 1% were considered. Sex chromosomes were excluded from the analyses. We took the logarithms of the PEER-normalized expression levels for calculations to correct it for far outliers and standardized the data. We started the analysis by performing principal component analysis (PCA) in the 23,722-dimensional space of gene expression rates. Based on the Scree Plot (Additional file 2: Figure S27), we decided to keep the first 100 principal components. Only the genes having contributions to these components not smaller than 0.01 were considered in the further analyses. By running this procedure, we got 14,853 genes of the largest contribution to the variance in gene transcription between samples. Every SV lying in the same CCD as one of these genes was tested for being eQTL for this gene. Least-squares linear regression between expression rates and genotypes was performed for each gene-SV pair. The slopes of the linear models were tested for statistical significance. First, for each linear model, two-sided *p* value was calculated in the test with a null hypothesis that slope is 0 (Wald test with *t*-distribution of the test statistics). Second, for each gene, we permutated the expression rates relative to genotypes 1000 times, recalculating at each iteration the linear regression for each gene-SV pair and recording the minimal *p* value among all pairs. Adjusted *p* values were calculated for each gene by dividing the ranks of the observed *p* values in the list of *p* values obtained in permutations by the number of permutations. Finally, to correct for multiple testing across genes, we applied the Benjamini-Hochberg procedure to the adjusted *p* values, estimating *q* values. At FDR 0.1, we found 192 genes with eQTLs. The same procedure was employed to identify eQTLs for housekeeping genes, except that PCA step was omitted. By mapping the names of housekeeping genes reported in Eisenberg and Levanon [49] on GENCODE (version 12), we obtained a list of 3784 genes. We found eQTLs for 33 of them. Lists of discovered eQTLs are provided in Additional file 1: Table S7 and S8.

### Immune-related genes

Names and coordinates on the hg19 assembly of immunity genes were downloaded from InnateDB [61]. The gene names were mapped on GENCODE (version 12) for the purpose of the eQTL analysis. The final gene set contained 1051 elements.

### ChIP-seq signal tracks

Raw sequencing data from ChIP-seq experiments published by Kasowski et al. [23] was processed to obtain signal tracks of CTCF and histone marks for 10 lymphoblastoid cell lines (GM12878, GM10847, GM12890, GM18486, GM18505, GM18526, GM18951, GM19099, GM19238, GM19239) [24]. The sequencing reads were aligned to the hg19 assembly using Bowtie2 (version 2.3.4.1) aligning tool [62]. The alignments were then passed to the bamCoverage utility from the deepTools2.0 (version 3.0.2) toolkit [63] to obtain RPM values genome-wide (the following command was evoked: bamCoverage -b input.bam -o output.bw -of bigwig --binSize 10 --numberOfProcessors max/2 --normalizeUsing CPM --ignoreForNormalization chrX --extendReads --samFlagInclude 64). Sequencing reads for every sample and for every experimental replicate were processed separately. Signals prepared for different biological replicates but for the same sample were merged to an averaged signal using the mean operator from the WiggleTools1.2 package [64]. Each CTCF signal track included in the figures presents RPM values for a particular genomic region divided by the maximal value of the signal in this region.

The same post-alignment steps were applied to obtain signal tracks for SMC3 and RAD21 from the alignments downloaded from ENCODE (Additional file 1: Table S5).

H3K27ac, H3K4me3, H3K4me1, and DNase-seq data analyzed in the “Regulation of gene transcription altered by topological variations in population” section was downloaded from ENCODE in a form of bigWig files containing signal fold change over control (Additional file 1: Table S5).

Presented RNAPII signals were downloaded from the UCSC database (Additional file 1: Table S5).

### Phased ChIP-seq signal tracks

Haplotype-specific CTCF and H3K4me1 ChIP-seq signals for 10 lymphoblastoid cell lines (GM12878, GM10847, GM12890, GM18486, GM18505, GM18526, GM18951, GM19099, GM19238, GM19239) were obtained from the raw sequencing data [24]. The reads sequenced for a particular cell line were aligned with Bowtie2 (version 2.3.4.1) aligning tool to the individualized nucleotide sequences of maternal and paternal chromosomes of this cell line. Only perfectly aligned reads were considered as valid. The sequences of maternal and paternal chromosomes were prepared with the vcf2diploid (version 0.2.6) tool from the AlleleSeq pipeline [65] using SNP phasing information from phase 3 of the 1000 Genomes Project. Additionally, the sequences of maternal and paternal chromosome 1 including SNP and SV phasing information from phase 3 of the 1000 Genomes Project were prepared. VCF files for chromosome 1 were processed by a custom script to represent alternative alleles as a sequence rather than SV identifier. Then, CTCF and H3K4me1 ChIP-seq data for 10 lymphoblastoid cell lines were mapped to those maternal and paternal sequences. To enable the comparison of phased ChIP-seq signals including SNP and SV information between individuals, aligned reads were remapped to hg19 reference with CrossMap (version 0.2.5) [66]. This step required chain files which were prepared as described in Minimal Steps For LiftOver [67]. Separate ChIP-seq signals for maternal and paternal chromosomes of the individual samples were calculated from the alignments prepared for the respective chromosomes analogously to the non-phased signals.

### Linkage disequilibrium calculation

Linkage disequilibrium (LD) between selected SVs was calculated in the CEU population. Genotype information for 99 individuals from CEU population was extracted from phase 3 of the 1000 Genomes Project vcf files and passed to vcftools (version 0.1.15) [68] to convert it into PLINK PED format (the following command was evoked: vcftools –vcf input_svs.vcf –plink-tped –out plinksvs). As some variants were multiallelic, the input vcf file was first processed with bcftools (version 0.1.19) to convert multiallelic variants to biallelic (the following command was evoked: bcftools norm –m - -o svs.vcf –O v input_svs.vcf). Then, LD between selected SVs measured as *r*^2^ value was calculated with PLINK (version 1.07) [69] (the following commands were evoked: plink –tfile plinksvs –make-bed –out out_svs; plink –bfile out_svs –r2 –ld-window-kb 1000 –ld-window 99999 –ld-window-r2 0.5).

### Modeling three-dimensional chromatin structures with 3D-GNOME

The ChIA-PET datasets typically consist of two types of interactions: high-frequency PET clusters (in the order of tens of thousands) representing strong, specific chromatin interactions, and singletons, numerous (in the order of tens of millions), but representing mostly non-specific and spurious contacts.

To make the best use of the information carried by these two distinct types of contacts, we employed a multiscale approach: first, we used the singletons to guide the low-resolution, megabase-scaled modeling, and then we used PET clusters to refine the obtained structures, achieving resolutions up to a few kilobases. We note that this approach is consistent with the widely accepted model of genome organization, in which the main roles are played by topological domains and chromatin loops. Here, at the stage of the low-resolution modeling, we attempt to position the topological domains relative to each other, and in the high-resolution, we model the position and shape of individual chromatin loops.

### Low-resolution (chromosome level) modeling

The structure of a chromosome is represented using a “beads on a string” model. First, the chromosome is split into a number of approximately megabase-sized regions. Ideally, each region would correspond to a single topological domain. In practice, the split is made based on the patterns of PET clusters interactions (as a consequence, different regions typically will have different lengths (see [26] for details of the procedure)). Next, singleton heatmaps are created (much like the widely used Hi-C heatmaps, but with unequal bins). We treat an interaction frequency *f*_*ij*_ between a pair of regions *i* and *j* as a proxy of 3D distance *d*_*ij*_ between corresponding beads, assuming an inverse relationship ${d}_{ij}\sim c{f}_{ij}^{-\alpha }$, with *α* being a scaling exponent, and use Monte Carlo simulated annealing to position the beads to minimize the energy function $E=\sum \limits_{i,j}{\left({d}_{ij}-{r}_{ij}\right)}^2$, where *r*_*ij*_ is the actual distance between beads corresponding to regions *i* and *j.*

### High-resolution modeling

In the second step, we model the shape and position of chromatin loops inside a single domain. We begin by splitting the interaction network given by PET clusters contained within a domain into a number of disjoint connected components that we call *blocks*. This allows us to model blocks independently. The modeling of each block is carried out in 2 steps. First, in the anchor step, we position the anchors of the loops identified by ChIA-PET relative to each other. The preferred distance between a pair of anchors *i* and *j* connected by a loop depends on the frequency of the PET cluster solely and is given by ${d}_{ij}=\delta +\alpha {e}^{-\upsilon \left({f}_{ij}-\gamma \right)}$, where *δ*, *α*, *υ*, and *γ* are all parameters (if the anchors are not connected, then *d*_*ij*_ is not specified). The energy function is identical in the form to the one used at the chromosome level, and we again use Monte Carlo simulations to find the optimal arrangements of the anchors. Then, in the subloop step, we keep the anchors’ positions fixed, and we try to model the loops so that their shape, as well as relative position to other loops, best fit both the data and the physical constraints. Each loop is represented by *k* subanchor beads inserted between the neighboring anchors. We define stretching and bending energy terms as ${E}_s=\sum \limits_i{\left({r}_{i,i+1}-{N}_{i,i+1}^{\beta}\right)}^2$ and ${E}_b=\frac{1}{2}\sum \limits_i{\left(1-{\hat{\upsilon}}_{i-1,i}\cdotp {\hat{\upsilon}}_{i,i+1}\right)}^2$, where *N*_*j*, *j* + 1_ is a genomic distance between anchors *j* and *j + 1*, ${\hat{\upsilon}}_{j,j+1}$ is a unit vector pointing from anchor *j* to *j + 1*, and *β* is a constant parameter. To model the influence of short-range singleton interactions, we calculate the expected distances between all subanchor beads given only the physical constraints and then modify these distances based on the high-resolution singleton heatmaps for blocks. These updated distances are used in the third energy term ${E}_h=\sum \limits_{i,j}{\left({d}_{ij}-{r}_{ij}\right)}^2$, with *d*_*ij*_ and *r*_*ij*_ defined previously, but now for subanchor beads. The total energy function is simply *E*_*s*_ + *E*_*b*_ + *E*_*h*_, and again, the Monte Carlo simulation is used for the optimization.

### Modeling impact of SVs onto the three-dimensional chromatin structure

The algorithm modifies the reference genomic interactions and topological domains introducing information on SVs. The resulting genomic interaction data is then passed to the 3D-GNOME modeling engine to obtain predicted 3D structures adjusted for SVs.

Anchors intersected by deletions are removed from the reference interaction pattern. As a result, all the interactions stemming from these anchors are eliminated yielding a structure with fewer loops, and with the loops directly neighboring the deletion being shorter or longer depending on the interaction pattern and the deletion size. If an outermost anchor in a CCD is intersected by a deletion, the boundary of the CCD is deleted and the CCD is fused with a neighboring CCD. CCDs covered by deletions are removed and the ones partially excised fused with neighboring CCDs. The anchors intersected by duplications are duplicated along with the contacts they have with other genomic segments in a way that interactions with anchors located upstream from the duplication are kept by the affected anchor and downstream interactions in respect to the duplication are acquired by the duplicate. The duplicate is positioned downstream from the affected anchor. If larger genomic fragments are duplicated, interactions between anchor duplicates are established equivalently to those between the duplicated anchors and anchor duplicates are not linked with the anchors of the original fragments. If a duplication expands over a CCD boundary, parts of the CCDs placed at the breakpoint after duplication are fused. Introducing duplications also results in elongation of loops overlapping the duplicated site. Inversions of genomic segments containing anchor sites result in changing positions of the anchors and its directionality relative to other anchors. After an anchor is inversed, we delete all its previous contacts and link it to the closest anchor with which it can form a convergent loop to reflect the preference of CTCFs to have symmetric conformation in dimers [15, 17]. In case of undirected protein targets, we link an inverted anchor with the closest anchor with no additional criteria. If the inverted anchor indicates a CTCF-mediated CCD border and it forms a convergent loop with the other border of the CCD, the border is removed. The anchors which lose all their connections as a consequence of SV introduction are linked with the closest anchor of orientation enabling the formation of convergent loop, in case of CTCF interaction networks, or closest anchor with no additional criteria in case of undirected protein targets. The insertions detected in the 1000 Genomes Project are almost solely insertions of transposable elements, which do not introduce new CTCF binding sites to the genome. Nevertheless, our algorithm enables introducing new CTCF binding sites to domain structures. Along with such insertions, new contacts are introduced between the inserted anchors and their neighbors.

The algorithm accounts also for SVs that miss the CTCF binding sites, but the introduction of these results only in shortening or extending the corresponding chromatin loops.

### Comparison of CTCF interaction segments among different individuals

In order to assess the fraction of CTCF interaction segments conserved among different lymphoblastoid cell lines, we calculated a number of CTCF anchors identified in GM12878, which were intersected with CTCF ChIP-seq peaks called in the remaining lymphoblastoid cell lines.

Since the available sets of CTCF ChIP-seq peaks for multiple lymphoblastoid cell lines [24] differed highly in size (Additional file 2: Figure S28), we performed an additional filtering on them. Only those CTCF ChIP-seq peaks which intersected with consensus CTCF binding sites were selected for each of the lymphoblastoid cell lines. The consensus CTCF binding sites were collected from the Ensembl Regulation 92 database [70]. They were identified by first performing a genome segmentation based on a variety of genome-wide assays from multiple cell types (including histone modification ChIP-seqs, TF ChIP-seqs, DNase-seq) and selecting segmentation state corresponding to CTCF peaks, and second, annotating the position of CTCF binding sites within the peaks using JASPAR position weight matrix MA0139.1, for more details, see the Ensembl website.

The consensus CTCF binding sites were downloaded via BioMart interface in genomic coordinates of hg38 assembly and converted to hg19 coordinates using UCSC liftOver tool [71].

Comparable datasets were obtained by the filtering (Additional file 2: Figure S29).

When using these filtered datasets, over 99% of interacting anchors occupied by CTCF peaks in GM12878 cell were identified as supported by CTCF peaks in each of the other lymphoblastoid samples. However, we note that a similar rate of 83% and higher is observed when using unfiltered sets of CTCF ChIP-seq peaks (Additional file 2: Figure S30).

### Aggregate analysis of ChIP-seq signals in altered interaction anchors

In order to analyze the overall behavior of ChIP-seq signal in interacting genomic segments affected by deletions or duplications, we performed an aggregate analysis. The same procedure was applied regardless of SV type (deletion or duplication) and ChIP-seq target protein (CTCF or RNAPII). We describe it using an example of CTCF interacting segments affected by deletions.

First, CTCF anchors intersected by deletions exhibited by at least one of the 10 lymphoblastoid cell lines with available CTCF ChIP-seq data [24] were identified. For each such anchor, 200 bins were defined—100 bins of equal size covering an anchor and 50 equal-size bins covering genomic regions 500 bp upstream and downstream from the anchor. Averaged raw CTCF ChIP-seq signal was calculated in every bin for every sample. For each sample, a mean of the signal over the whole genome was found and subtracted from the extracted binned signal values. The maximal mean from all the samples was then added to the values to make them positive. Obtained values were then averaged over the samples exhibiting and not exhibiting the deletion. The log2 of the ratio of the signal values obtained for the first group to the ones obtained for the second group was calculated. The log2 fold changes calculated for all the anchors affected by deletions were then averaged and plotted in Additional file 2: Figure S12A.

## Additional files

Additional file 1: Supplemental tables. (XLSX 12146 kb)

Additional file 2: Supplemental figures. (PDF 17323 kb)

## Data Availability

The authors declare that the data supporting the findings of this study are available within the supplemental files or in public repositories to which references are given in the paper and supplemental information files. ChIA-PET dataset supporting the conclusions of this article was downloaded from the Gene Expression Omnibus (GEO), accession number GSE72816, https://www.ncbi.nlm.nih.gov/geo/query/acc.cgi?acc=GSE72816 [18]. Positions of the CTCF- and RNAPII-mediated chromatin interactions and chromatin contact domains in the hg19 reference genome assembly are additionally provided in Additional file 1: Table S3, S4, and S6 respectively. Hi-C data analyzed in this study was downloaded from GEO, accession number GSE63525, https://www.ncbi.nlm.nih.gov/geo/query/acc.cgi?acc=GSE63525 [21]. CTCF, SMC3, RAD21 and RNAPII ChIP-seq datasets used for chromatin interactions filtering and support were downloaded from The Encyclopedia of DNA Elements (ENCODE) [52]; the accession numbers and URLs of these datasets are provided in Additional file 1: Table S5. The set of ChIA-PET interactions filtered by the co-occupancy by CTCF and cohesin (SMC3 and RAD21 subunits) is provided in Additional file 1: Table S1. CTCF and histone marks in ChIP-seq datasets for multiple lymphoblastoid cell lines were downloaded from GEO, accession number GSE50893, https://www.ncbi.nlm.nih.gov/geo/query/acc.cgi?acc=GSE50893 [24]. RNAPII ChIP-seq signals for multiple lymphoblastoid cell lines were downloaded from ENCODE; detailed accession information is provided in Additional file 1: Table S5. Structural variants analyzed in this study were adopted from phase 3 of the 1000 Genomes Project, ftp://ftp.1000genomes.ebi.ac.uk/vol1/ftp/phase3/integrated_sv_map/ALL.wgs.mergedSV.v8.20130502.svs.genotypes.vcf.gz [25]. NHGRI-EBI GWAS Catalog released on January 31, 2018, was analyzed in this study, http://ftp.ebi.ac.uk/pub/databases/gwas/releases/2018/01/31/gwas-catalog-associations.tsv [32]. RNA-seq dataset used for eQTL analysis was downloaded from ArrayExpress, accession number E-GEUV-1, https://www.ebi.ac.uk/arrayexpress/experiments/E-GEUV-1/ [42]. Genomic interactions identified in the GM12878 cell line through Capture Hi-C experiments were used for comparison. The dataset was downloaded from ArrayExpress, accession number E-MTAB-2323, https://www.ebi.ac.uk/arrayexpress/experiments/E-MTAB-2323/ [48]. Gene annotations from GENCODE versions 12 and 27 were used throughout the study [58]. Immune-specific genes were selected based on the InnateDB annotation, https://www.innatedb.com/annotatedGenes.do?type=innatedb [61]. Chromatin state segmentation for the GM12878 cell line by ChromHMM and TFBS clusters were downloaded from ENCODE and the detailed accession information is provided in Additional file 1: Table S5. Python code developed to include SV information in chromatin interaction patterns is available at https://bitbucket.org/4dnucleome/spatial_chromatin_architecture under the BSD 2-Clause License. The version used in the manuscript is deposited in zenodo 10.5281/zenodo.2837248 [72]. The algorithm was integrated with the 3D-GNOME modeling engine [26] and a visualization tool into a web service (3D-GNOME 2.0) [28].
