# Correction to: Spatial chromatin architecture alteration by structural variations in human genomes at the population scale

**DOI:** 10.1186/s13059-019-1780-6

**Published:** 2019-09-03

**Authors:** Michal Sadowski, Agnieszka Kraft, Przemyslaw Szalaj, Michal Wlasnowolski, Zhonghui Tang, Yijun Ruan, Dariusz Plewczynski

**Affiliations:** 1 Centre of New Technologies, University of Warsaw, Banacha 2c, 02-097 Warsaw, Poland; 2 Faculty of Physics, University of Warsaw, Pasteura 5, 02-093 Warsaw, Poland; 3 Faculty of Mathematics and Information Science, Warsaw University of Technology, Koszykowa 75, 00-662 Warsaw, Poland; 4 Centre for Innovative Research, Medical University of Bialystok, Kilinskiego 1, 15-089 Bialystok, Poland; 5 I-BioStat, Hasselt University, Agoralaan building D, BE3590, Diepenbeek, Belgium; 6 Zhongshan School of Medicine, Sun Yat-sen University, Guangzhou, 510080 China; 7 The Jackson Laboratory for Genomic Medicine, 10 Discovery Drive, Farmington, CT 06032 USA

**Correction to: Sadowski et al. Genome Biol (2019) 20:148**

**https://doi.org/10.1186/s13059-019-1728-x**

Following publication of the original article [1], it was noticed that the incorrect Fig. 2 and Fig. 3. were processed during production. It was also noticed that Fig. 4a was processed with a superfluous “1e7” symbol in the upper right corner.

The corrected Fig. 2, Fig. 3 and Fig. 4 are provided below. The original article has been corrected. The publishers apologize for the error.

**Fig. 2:**
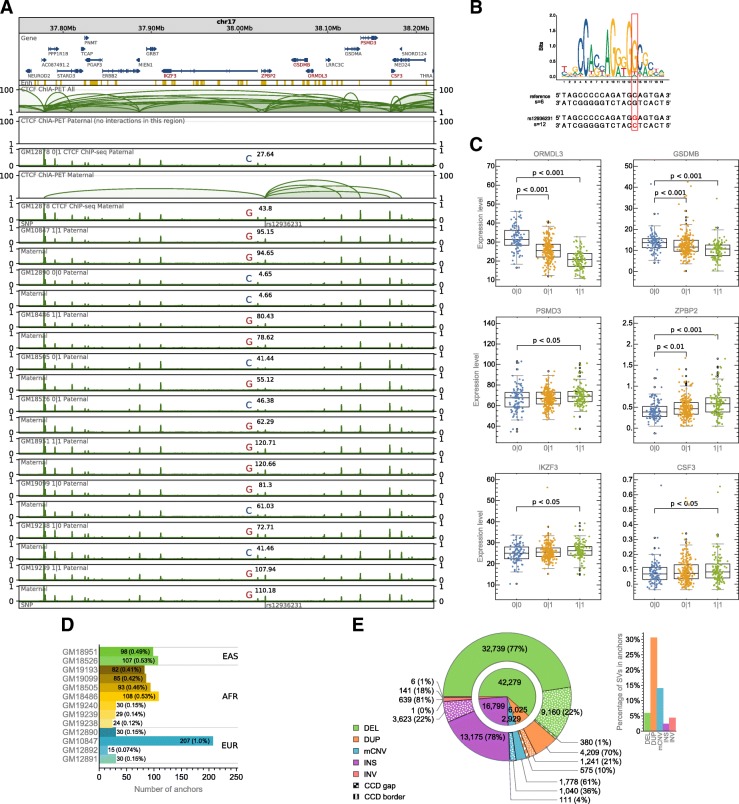
Predicting the impact of SVs on the chromatin topology. **a** Browser view of a 0.5-Mb genomic segment with asthma-associated SNP rs12936231 identified in a part of the human population. SNP rs12936231 alters the sequence of a CTCF motif involved in interactions. Haplotype-specific CTCF signals from 10 lymphoblastoid cells are presented along with haplotype-specific CTCF ChIA-PET interactions from GM12878 (only a subset of all interactions can be identified as specifically paternal/maternal as it is done based on allele-specific SNPs emerging at the interaction anchors). For each track, ChIP-seq signal values (originally in RPMs) were divided by the maximal value of the signal in the visualized region. Sum of the signal values over the genomic region occupied by the SNP-affected interaction anchor together with the genotype is marked in each signal track. **b** Comparison of sequences and scores of CTCF binding motifs carrying the reference C and the alternative G alleles of rs12936231. **c** Differences in gene transcription rates between genotypes set for rs12936231. Genes exhibiting differences in transcription which pass Mann-Whitney test with *p* value < 0.05 were reported. Center lines show the medians; box limits indicate the 25th and 75th percentiles; whiskers extend 1.5 times the interquartile range (IQR) from the 25th and 75th percentiles; outliers are represented by rings; far outliers (points beyond 3 times the IQR) are not represented by any element of box plots. *n* = 101, 227, 117 sample points. **d** CTCF anchors from GM12878 not intersected with CTCF ChIP-seq peaks identified in different lymphoblastoid cells. The anchors were filtered by consensus CTCF binding sites (see the “Methods” section). **e** Number of SVs, divided by type, intersecting (in case of interaction anchors), covering (in case of CCD boundaries), or contained in (in case of CCDs and CCD gaps) different genomic structural elements

**Fig. 3:**
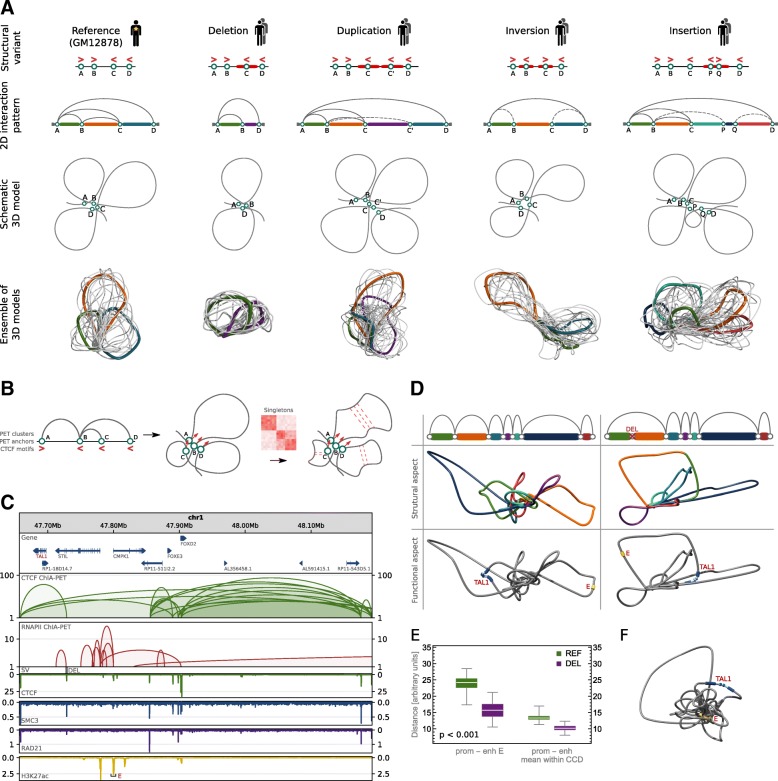
Computational algorithm for modeling topological alterations caused by SVs. **a** Predicted impact of particular SV types on looping structure of the genome. Simplified chromatin looping patterns and 3D models are presented for the reference and its SV-altered versions. **b** Scheme presenting the chromatin modeling method at the level of loops. The method uses PET clusters, singletons, and orientations of CTCF binding motifs to accurately model the genome looping structures. **c** Browser view of a topological domain containing TAL1 gene and a deletion causing its activation. The deletion removes CTCF insulating the TAL1 promoter from enhancer E. CTCF and RNAPII ChIA-PET interactions are shown along with ChIP-seq tracks for CTCF, cohesin subunits (SMC3 and RAD21), and H3K27ac which marks the enhancer E. **d** Models presenting 3D structure of the TAL1 locus without the deletion (left column) and with the deletion (right column). Schematic drawings of loops shown in **c** (first row); 3D models with loops colored as on schematic drawings (second row); 3D models with TAL1 and enhancer E marked (third row). **e** Distance in 3D Euclidian space between the TAL1 promoter and enhancer E and mean distance between the promoter and enhancers located in the same CCD. In green, distribution of distances calculated in 3D models of the reference structure (REF), in purple—in models with the deletion introduced (DEL). For each case, 100 models were generated. The differences between REF and DEL groups are statistically significant (*p* values much less than 0.001), see Fig. 2c for box plot description. **f** 3D model of the TAL1 locus including RNAPII-mediated chromatin interactions

**Fig. 4:**
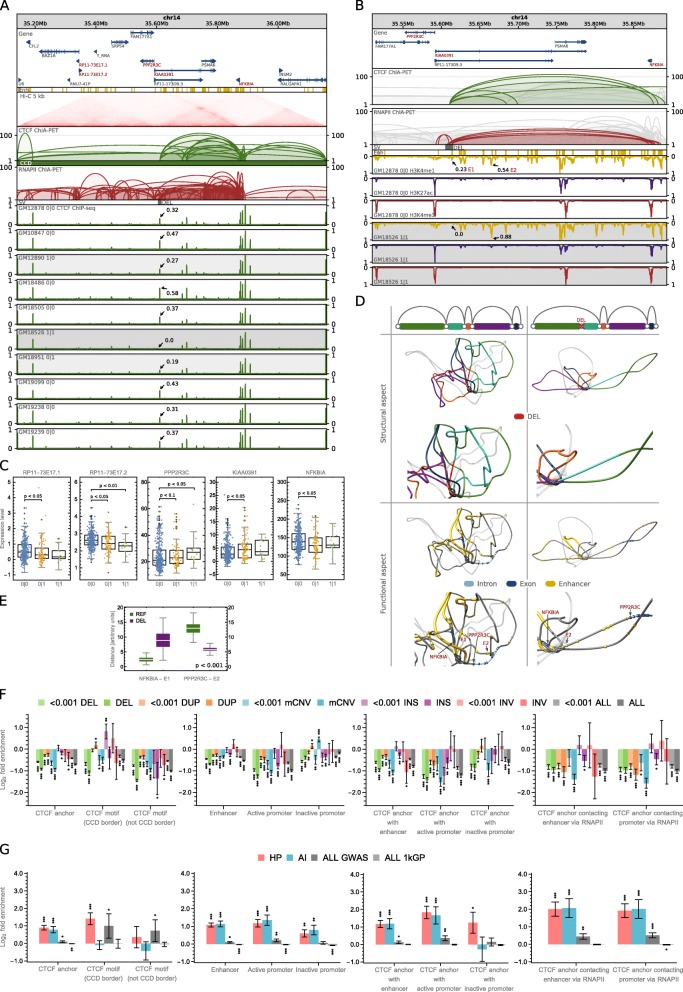
Impact of SVs on genome organization at the population scale. **a** Browser view of a 1-Mb genomic segment with a deletion identified in a part of the human population. The deletion removes a CTCF anchor with enhancer located in an intron of KIAA0391. CTCF ChIP-seq signals from 10 lymphoblastoid cells of different genotypes are presented for comparison. For each track, ChIP-seq signal values (originally in RPMs) were divided by the maximal value of the signal in the visualized region. The highest signal peak in the genomic region covered by the deletion is marked in each signal track. **b** Close-up on ChIA-PET interactions at the deletion site displayed above the ChIP-seq profiles of histone modifications for GM12878—no deletion and GM18526—homozygous deletion. H3K4me1 is primarily associated with active enhancers, H3K27ac—with active promoters and enhancers, H3K4me3—with promoters. Compare with Additional file 2: Fig. S7. **c** Differences in gene transcription rates between genotypes defined by the deletion. Genes exhibiting the differences in transcription which pass Mann-Whitney test with *p* value < 0.1 were reported, see Fig. 2c for box plot description. *n* = 346, 85, 14 sample points. **d** 3D models of the domain shown in **a** without the deletion (left column) and with the deletion (right column). Schematic drawings of loops shown in **b** (first row); 3D models with loops colored as on schematic drawings (second row); 3D models with NFKBIA and PPP2R3C genes (arrows are pointing toward the TSSs) and enhancers marked (third row). Every picture has its duplicated zooming in on the deletion site. **e** Distance in 3D Euclidean space between the NFKBIA promoter and enhancer E1 and between the PPP2R3C promoter and enhancer E2. In green, distribution of distances calculated in 3D models of the reference structure (REF), in purple—in models with the deletion introduced (DEL). For each case, 100 models were generated. The differences between REF and DEL groups are statistically significant (*p* values much less than 0.001), see Fig. 2c for box plot description. **f** Enrichment/depletion of genomic structural elements with SVs of different types and of different VAF (VAF < 0.001 and VAF ≥ 0.001). In case of CCD borders, only these fully imbedded in SV intervals are counted as affected, whereas for other structural elements ≥ 1 bp overlaps are counted. Error bars represent SD. **g** Enrichment/depletion of genomic structural elements with the 1000 Genomes Project SNPs (ALL 1kGP), all GWAS SNPs (ALL GWAS), GWAS SNPs associated with hematological parameters (HP), and with autoimmune diseases (AI). Error bars represent SD
