# Supplementary material for: Spatial chromatin architecture alteration by structural variations in human genomes at the population scale
Source: Genome Biol. 2019 Jul 30;20:148. doi: 10.1186/s13059-019-1728-x (PMC6664780; doi:10.1186/s13059-019-1728-x)
Supplement: Supplementary file 2 — Supplemental figures. (PDF 17323 kb) [file 13059_2019_1728_MOESM2_ESM.pdf]

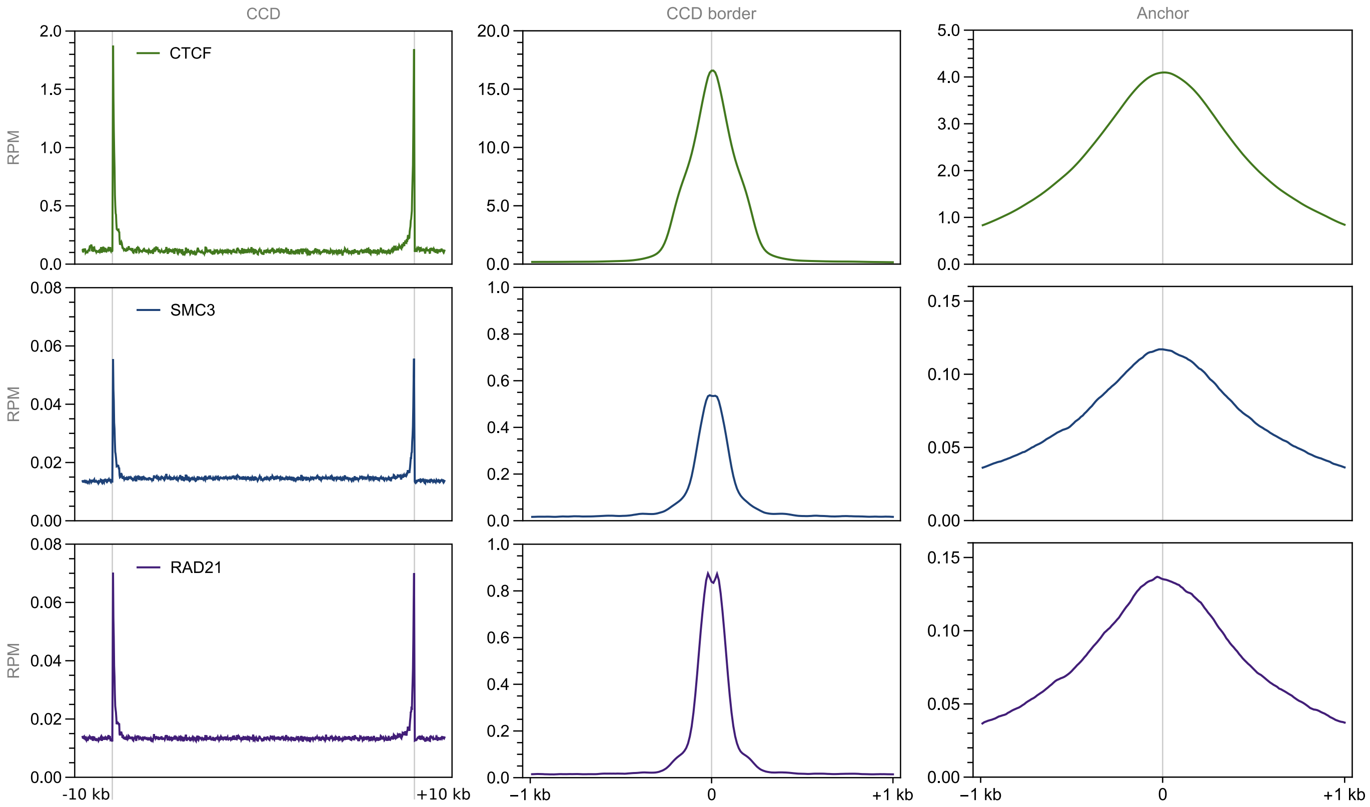

Fig. S1: Distributions of CTCF and cohesin signals around genomic structural elements. Aggregation plots showing CTCF, SMC3 and RAD21 binding distribution profiles around CCDs, CCD boundaries and CTCF anchors. CCDs were taken with ±10% extension proportional to the genomic regions enclosed by the domains. In case of CCD boundaries and CTCF anchors, 2 kb genomic regions centered at CTCF biding motifs indicating the boundaries and around the anchor centers were taken respectively. Each of the extended genomic segment corresponding to a CCD was split into 1,000 equal-sized bins and RPM value of binding signal along each bin was extracted. Similarly, 2 kb genomic region around each CCD boundary or CTCF anchor center was split into 200 equal-sized bins. The values across corresponding bins from all instances of a certain genomic structural element were averaged and plotted as a curve. Even though CTCF anchors are highly enriched with CTCF, SMC3 and RAD21 binding signals, those signals are significantly higher at CCD borders. In plots for whole CCDs, high peaks at boundaries can be observed as positions of CTCF anchors inside domains vary across CCDs and the signal from them averages out.

A

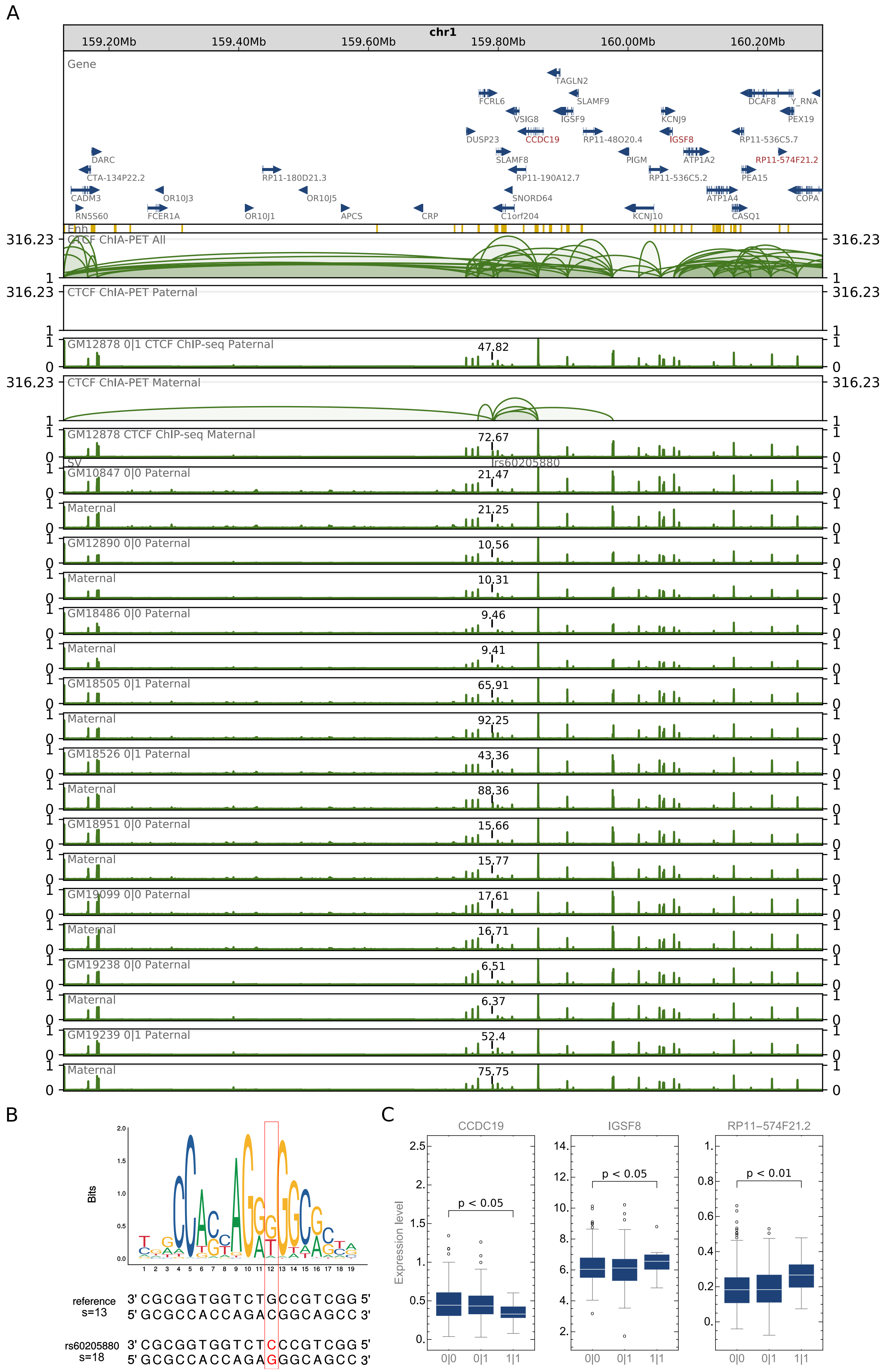
chr1
159.20Mb
159.40Mb
159.60Mb
159.80Mb
160.00Mb
160.20Mb
Gene
TAGLN2
FCRL6
SLAMF9
DCAF8
Y_RNA
VSIG8
IGSF9
KCNJ9
PEX19
DUSP23
CCDC19
RP11-48O20.4
IGSF8
RP11-536C5.7
DARC
SLAMF8
PIGM
ATP1A2
RP11-574F21.2
CTA-134P22.2
RP11-180D21.3
RP11-190A12.7
RP11-536C5.2
PEA15
CADM3
OR10J3
OR10J5
SNORD64
ATP1A4
COPA
RN5S60
FCER1A
OR10J1
APCS
CRP
C1orf204
KCNJ10
CASQ1
Enh
CTCF ChIA-PET All
CTCF ChIA-PET Paternal
GM12878 0|1 CTCF ChIP-seq Paternal
CTCF ChIA-PET Maternal
GM12878 CTCF ChIP-seq Maternal
SV
rs60205880
GM10847 0|0 Paternal
Maternal
GM12890 0|0 Paternal
Maternal
GM18486 0|0 Paternal
Maternal
GM18505 0|1 Paternal
Maternal
GM18526 0|1 Paternal
Maternal
GM18951 0|0 Paternal
Maternal
GM19099 0|0 Paternal
Maternal
GM19238 0|0 Paternal
Maternal
GM19239 0|1 Paternal
Maternal
47.82
72.67
21.47
21.25
10.56
10.31
9.46
9.41
65.91
92.25
43.36
88.36
15.66
15.77
17.61
16.71
6.51
6.37
52.4
75.75
316.23
B
Bits
reference s=13
3' CGCGGTGGTCTGCCGTCGG 5'
5' GCGCCACCAGACGGCAGCC 3'
rs60205880 s=18
3' CGCGGTGGTCTCCCGTCGG 5'
5' GCGCCACCAGAGGGCAGCC 3'
C
CCDC19
IGSF8
RP11–574F21.2
Expression level
p < 0.05
p < 0.05
p < 0.01
0|0
0|1
1|1

Fig. S2: SNP in a CTCF motif in region chr1:159130000-160300000. **a** Browser view of a 1 Mb genomic segment with SNP rs60205880 identified in a part of the human population. SNP rs60205880 alters sequence of a CTCF motif residing in an interaction anchor. Haplotype-specific CTCF ChIP-seq signals from 10 lymphoblastoid cell lines genotyped for this SNP are presented for comparison along with genes, enhancers and haplotype-specific (GM12878) CTCF ChIA-PET interactions located in this genomic segment. ChIP-seq signal from each sample is measured in RPMs and divided by the maximal value of the signal in the visualized region (separately for each homologous chromosome from the pair). In each individual signal track, value of the signal summed over genomic region occupied by the altered CTCF anchor is additionally marked. **b** Comparison of sequences and scores of CTCF motifs with reference and alternative alleles defined by rs60205880. CTCF sequence logo demonstrates the importance of particular nucleotide positions in the motif. **c** Differences in gene transcription rates between genotypes set for rs60205880. Only genes exhibiting differences in transcription which pass Mann-Whitney test with p-value < 0.05 were reported.

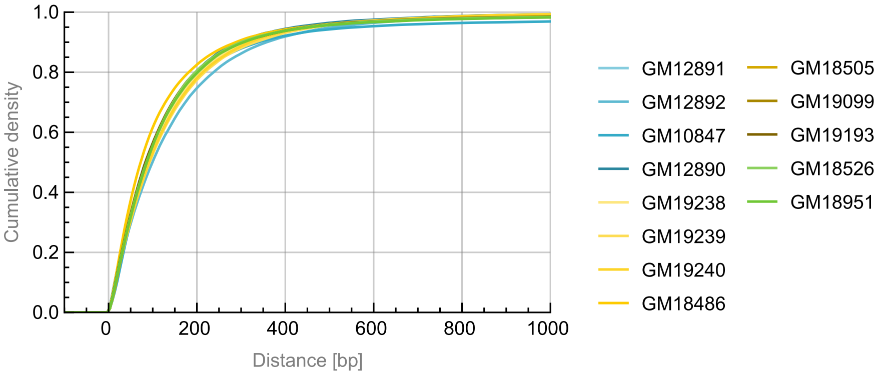

Fig. S3: Cumulative density plot of distances between CTCF ChIP-seq peaks located in GM12878 interaction anchors and closest CTCF ChIP-seq peaks from different lymphoblastoid cells.

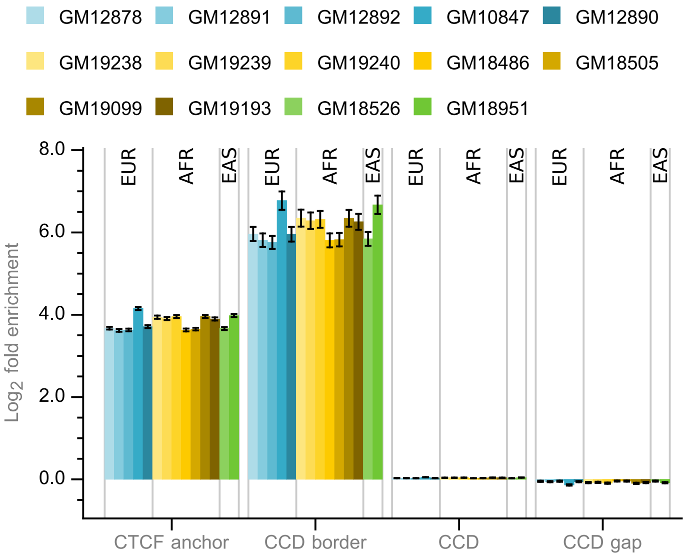

Fig. S4: Enrichment/depletion of structural elements identified in the GM12878 cell line with CTCF ChIP-seq peaks from different lymphoblastoid cells.

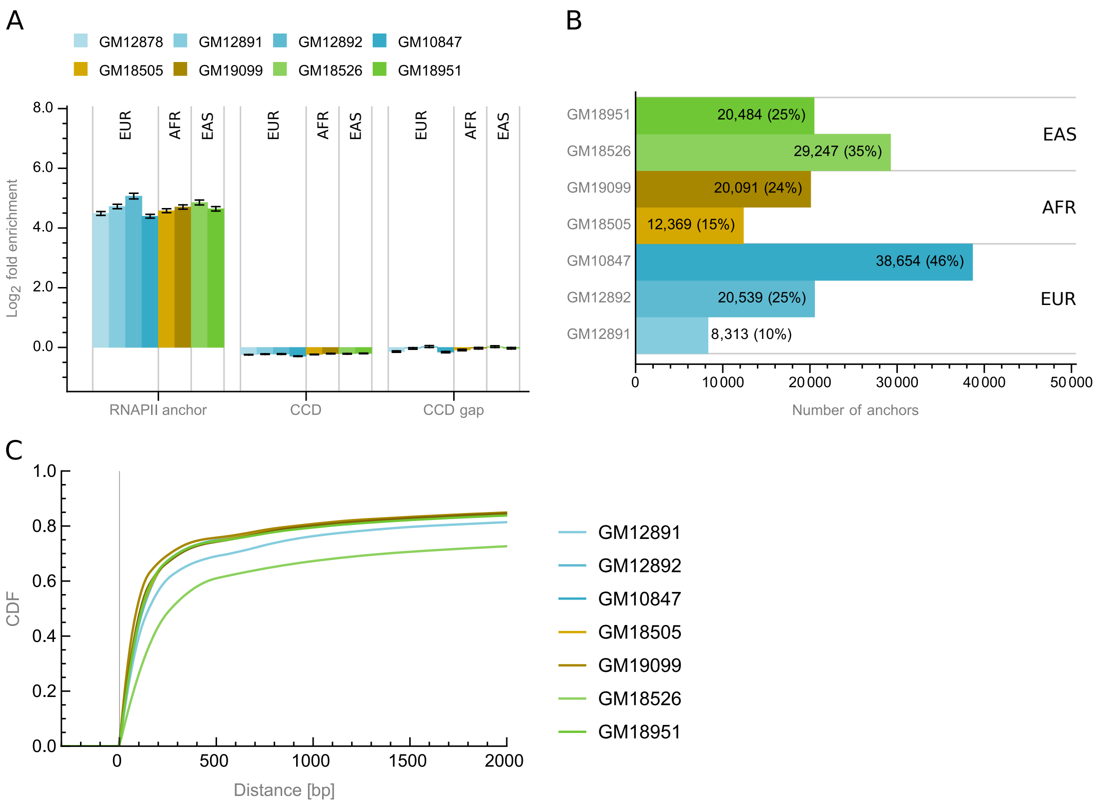

Fig. S5: RNAPII ChIP-seq peaks in interaction segments. a Enrichment/depletion of structural elements identified in the GM12878 cell line with RNAPII ChIP-seq peaks from different lymphoblastoid cells. **b** RNAPII anchors from the GM12878 not intersected with RNAPII ChIP-seq peaks identified in different lymphoblastoid cells. **c** Cumulative density plot of distances between RNAPII ChiP-seq peaks located in GM12878 interaction anchors and closest RNAPII ChIP-seq peaks from different lymphoblastoid cells.

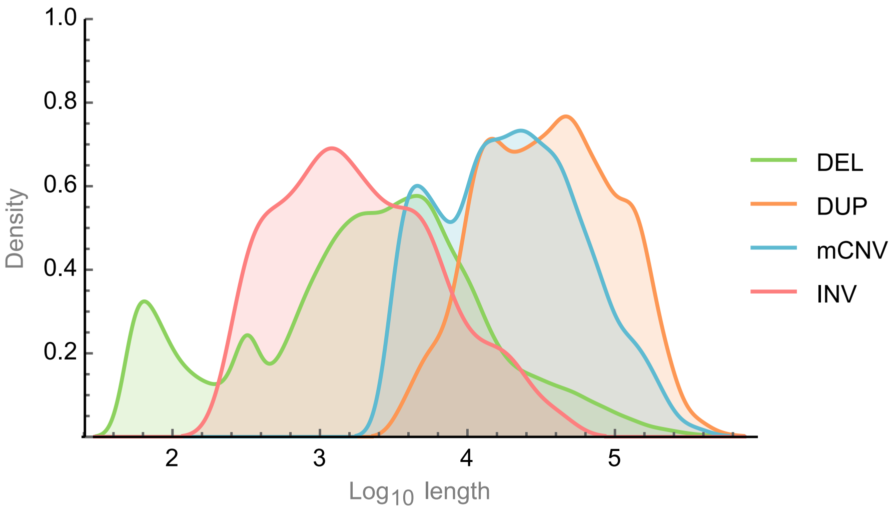

Fig. S6: Density plot showing genomic span distribution of SVs by type.

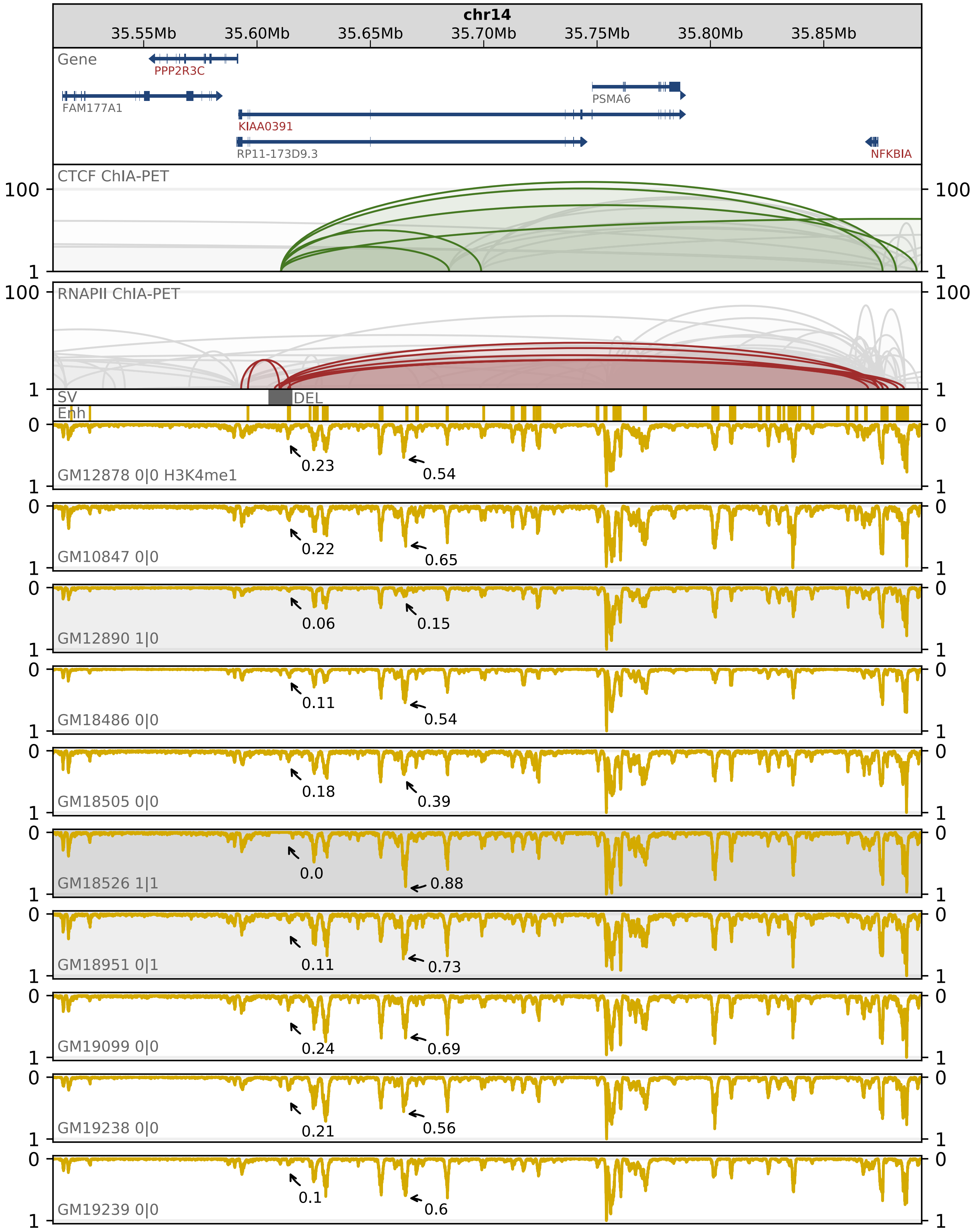
chr14
35.55Mb
35.60Mb
35.65Mb
35.70Mb
35.75Mb
35.80Mb
35.85Mb
Gene
PPP2R3C
FAM177A1
PSMA6
KIAA0391
RP11-173D9.3
NFKBIA
CTCF ChIA-PET
RNAPII ChIA-PET
SV
DEL
Enh
GM12878 0|0 H3K4me1
0.23
0.54
GM10847 0|0
0.22
0.65
GM12890 1|0
0.06
0.15
GM18486 0|0
0.11
0.54
GM18505 0|0
0.18
0.39
GM18526 1|1
0.0
0.88
GM18951 0|1
0.11
0.73
GM19099 0|0
0.24
0.69
GM19238 0|0
0.21
0.56
GM19239 0|0
0.1
0.6

Fig. S7: Deletion of a CTCF interaction anchor in region chr14:35510000-35893204. ChIA-PET interactions at the deletion site displayed above the ChIP-seq profiles of H3K4me1 (primarily associated with active enhancers) for a number of lymphoblastoid cell lines exhibiting different genotypes. The deletion removes a CTCF-mediated interaction anchor possibly involved in formation of insulated neighborhoods separating the PPP2R3C gene (upstream) from a group of enhancers (downstream) and promotes higher PPP2R3C transcription. H3K4me1 signal is notably stronger in deletion-affected homozygous GM18526 than in non-affected homozygous GM12878 in the enhancer region of interest. The signal seems comparable among samples with the reference genotype. It is higher for heterozygous GM18951, but not for heterozygous GM12890. The distinctive profile of the latter shows that other factors may also be involved in enhancer activity of this region in this cell. Compare with Fig. 4b.

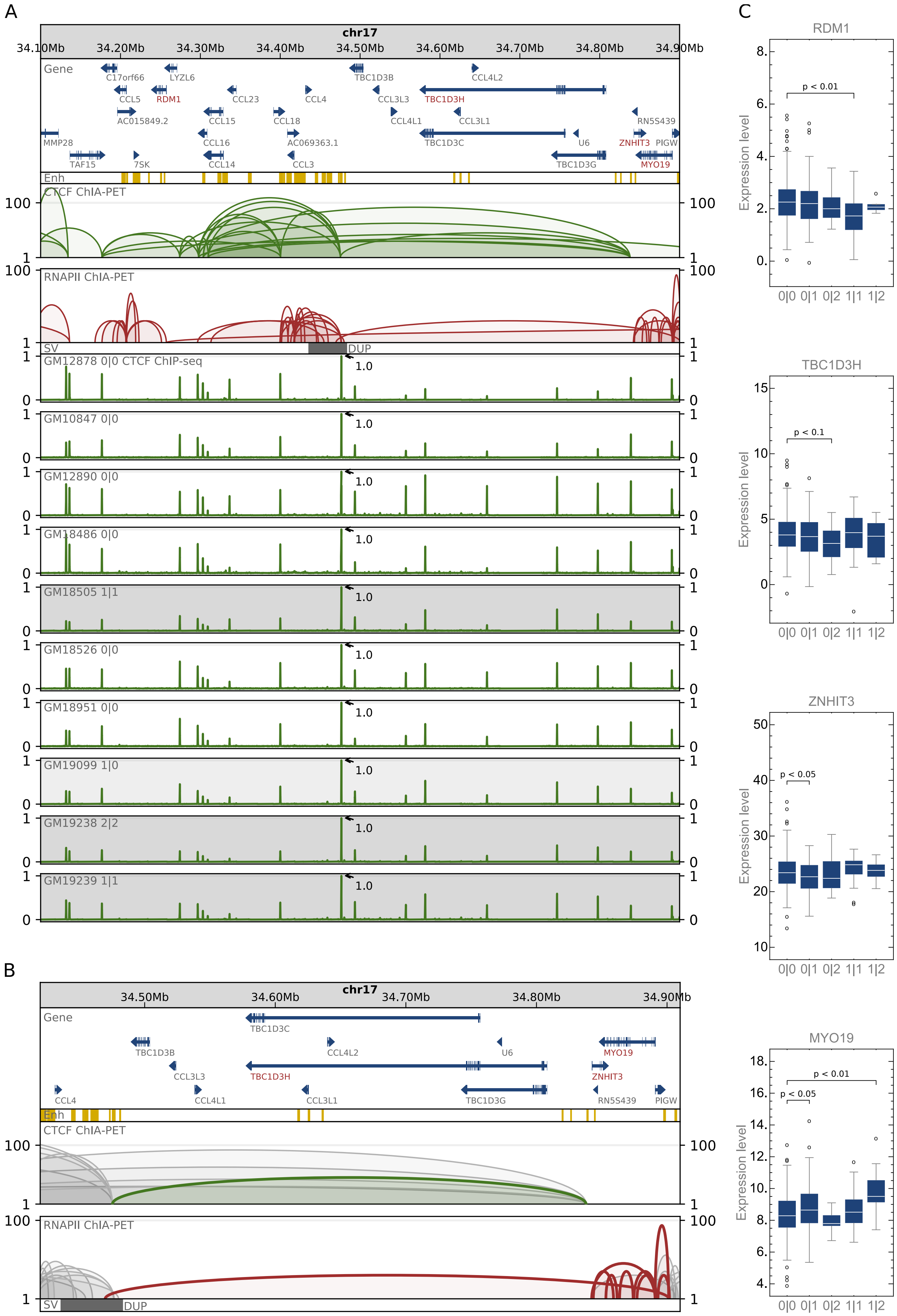
A
B
C
chr17
34.10Mb
34.20Mb
34.30Mb
34.40Mb
34.50Mb
34.60Mb
34.70Mb
34.80Mb
34.90Mb
Gene
Enh
CTCF ChIA-PET
RNAPII ChIA-PET
SV
DUP
GM12878 0|0 CTCF ChIP-seq
GM10847 0|0
GM12890 0|0
GM18486 0|0
GM18505 1|1
GM18526 0|0
GM18951 0|0
GM19099 1|0
GM19238 2|2
GM19239 1|1
1.0
RDM1
TBC1D3H
ZNHIT3
MYO19
Expression level
p < 0.01
p < 0.1
p < 0.05
0|0 0|1 0|2 1|1 1|2

Fig. S8: Duplication of a CTCF interaction anchor in region chr17:34100000-34900000. a Browser view of a 0.8 Mb genomic segment with duplication chr17:34436099-34482872 identified in a part of the human population, which duplicates a CTCF anchor containing enhancers. CTCF ChIP-seq signals from 10 lymphoblastoid cell lines genotyped for this duplication are presented for comparison along with genes, enhancers and CTCF and RNAPII ChIA-PET interactions located in this genomic segment. In each ChIP-seq signal track, the value of the highest signal peak in genomic region targeted by the SV is marked. ChIP-seq signal from each sample is measured in RPMs and divided by the value of the peak neighboring the annotated peak. **b** Close-up on the CTCF and RNAPII ChIA-PET interactions affected by duplication chr17:34436099-34482872. **c** Differences in gene transcription rates between genotypes set for duplication chr17:34436099-34482872. Only genes exhibiting differences in transcription which pass Mann-Whitney test with p-value < 0.1 were reported.

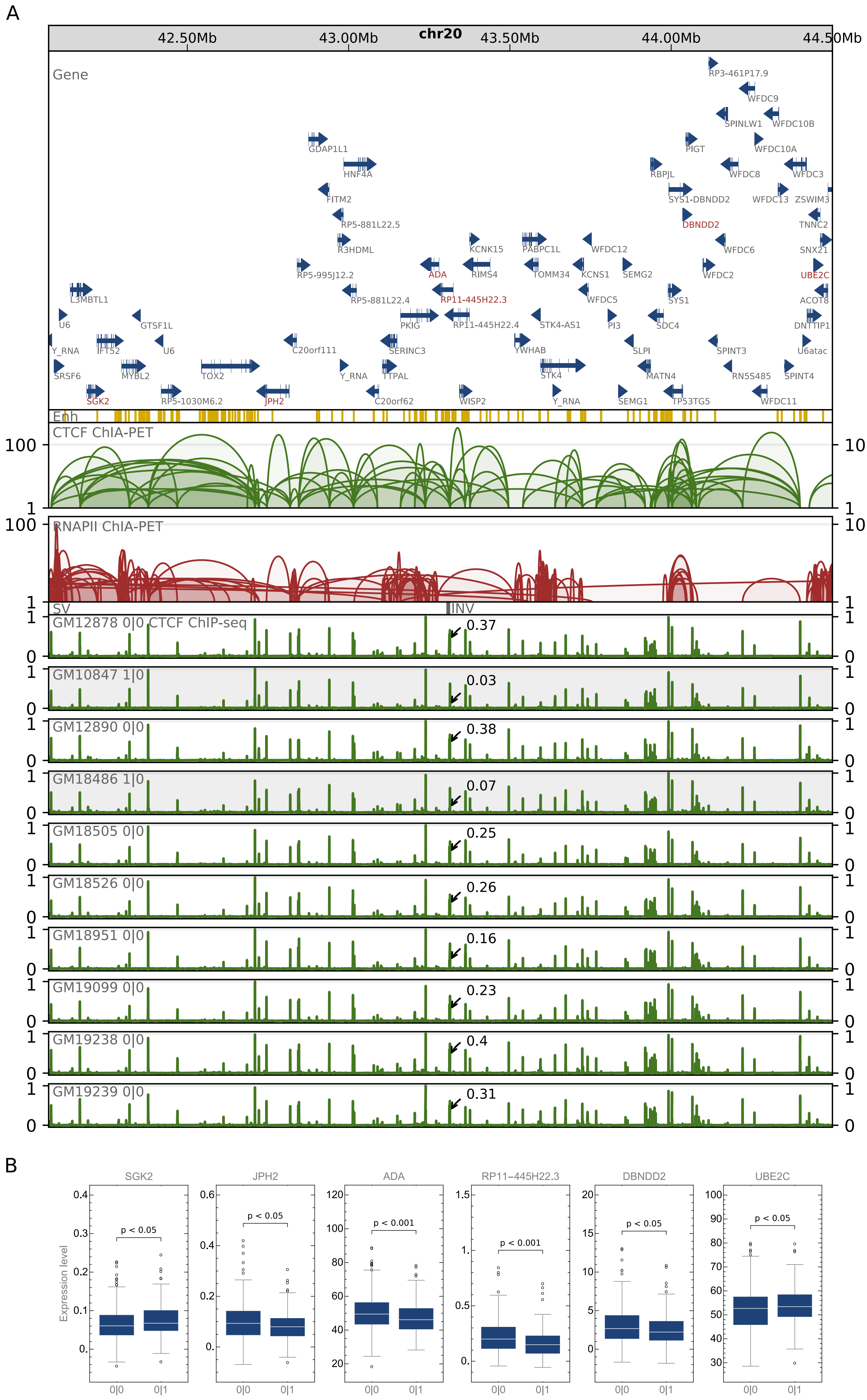
A
chr20
42.50Mb
43.00Mb
43.50Mb
44.00Mb
44.50Mb
Gene
Enh
CTCF ChIA-PET
RNAPII ChIA-PET
SV
INV
GM12878 0|0 CTCF ChIP-seq
0.37
GM10847 1|0
0.03
GM12890 0|0
0.38
GM18486 1|0
0.07
GM18505 0|0
0.25
GM18526 0|0
0.26
GM18951 0|0
0.16
GM19099 0|0
0.23
GM19238 0|0
0.4
GM19239 0|0
0.31
SGK2
JPH2
ADA
RP11-445H22.3
DBNDD2
UBE2C
B
Expression level
p < 0.05
p < 0.001
0|0
0|1

Fig. S9: Inversion in a CTCF interaction anchor in region chr20:42070000-44500000. a Browser view of a 2.5 Mb genomic segment with inversion chr20:43305746-43312261 identified in a part of the human population, which inverts a CTCF motif in an interaction anchor containing an enhancer. CTCF ChIP-seq signals from 10 lymphoblastoid cell lines genotyped for this inversion are presented for comparison along with genes, enhancers and CTCF and RNAPII ChIA-PET interactions located in this genomic segment. ChIP-seq signal from each sample is measured in RPMs and divided by the maximal value of the signal in the visualized region. In each individual signal track, value of the highest signal peak in genomic region targeted by the SV is additionally marked. **b** Differences in gene transcription rates between genotypes set for inversion chr20:43305746-43312261. Only genes exhibiting differences in transcription which pass Mann-Whitney test with p-value < 0.05 were reported.

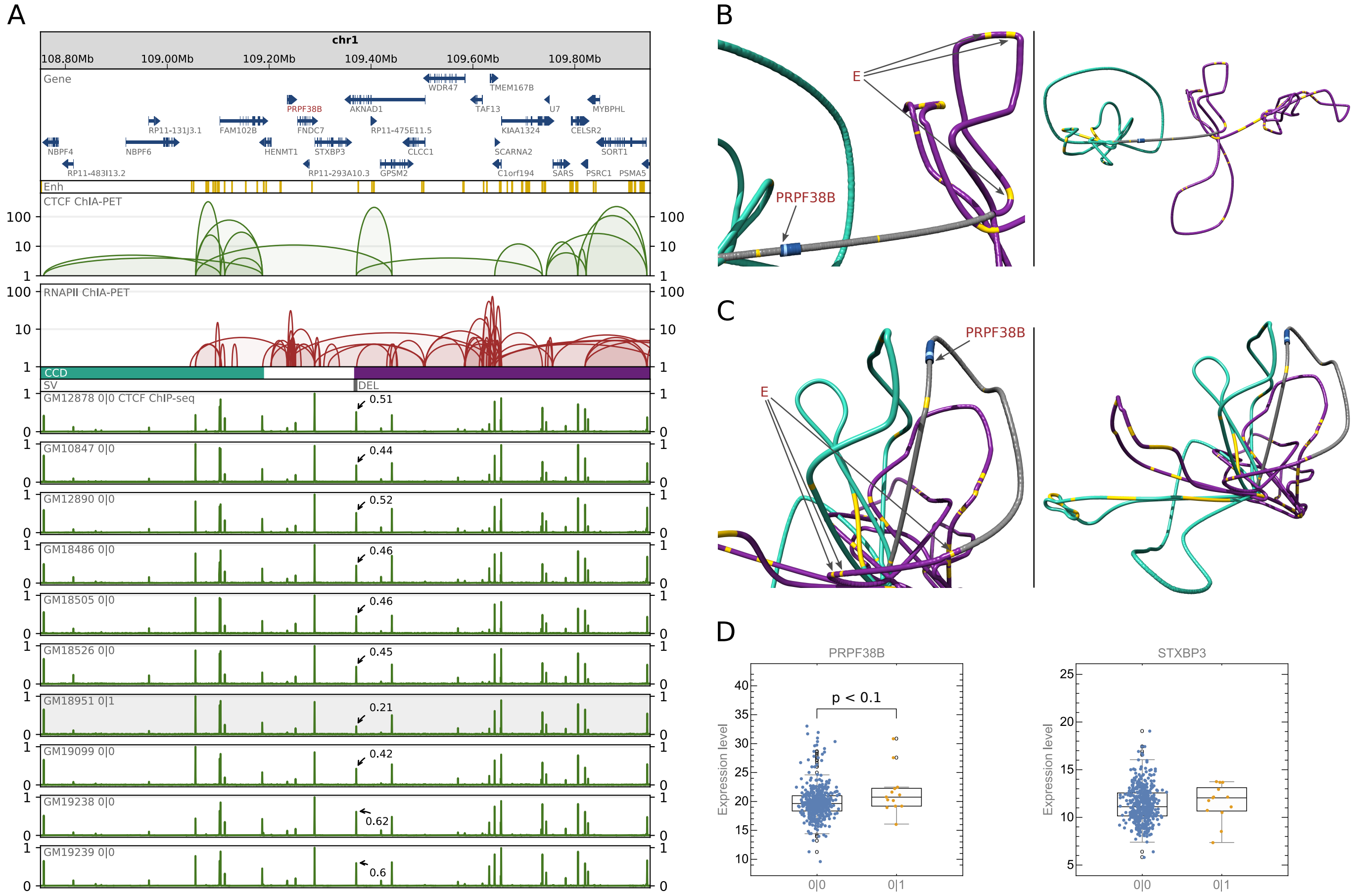

Fig. S10: Deletion of a CCD border in region chr1:108751000-109948000. a ChIA-PET interactions at the deletion site chr1:109366972-109372000 displayed above the CTCF ChIP-seq profiles for a number of lymphoblastoid cells exhibiting specific genotypes. The deletion removes a topological domain boundary. ChIP-seq signal from each sample is measured in RPMs and divided by the maximal value of the signal in the visualized region. In each individual signal track, value of the highest signal peak in genomic region targeted by the SV is additionally marked. **b** Models showing PRPF38B gene and enhancers relative arrangement in 3D (left) and arrangement of all chromatin loops in the genomic region shown in (a) (right). In genomes of the reference genotype, the PRPF38B gene is located at the genomic segment linking two topological domains and insulated from the enhancers located in those domains. **c** Similar to (b), but models represent topology of genomes having the deletion. The PRPF38B gene is fused with a bigger domain and becomes a part of a chromatin loop including a set of enhancers. The gene may spatially interact with those enhancers, which could be the cause of increased transcription rates (d). There is one another gene at the gap for which transcription rates are at our disposal (STXBP3), but its expression does not show significant differences (d). The reason for this could be sparsely represented genotype 0|1, but other mechanisms could also be involved. Experiments should be performed for comprehensive explanation. **d** Transcription of genes located at the gap neighboring the domain boundary excised by the deletion. The difference in PRPF38B transcription between genotypes is statistically significant (Mann-Whitney test).

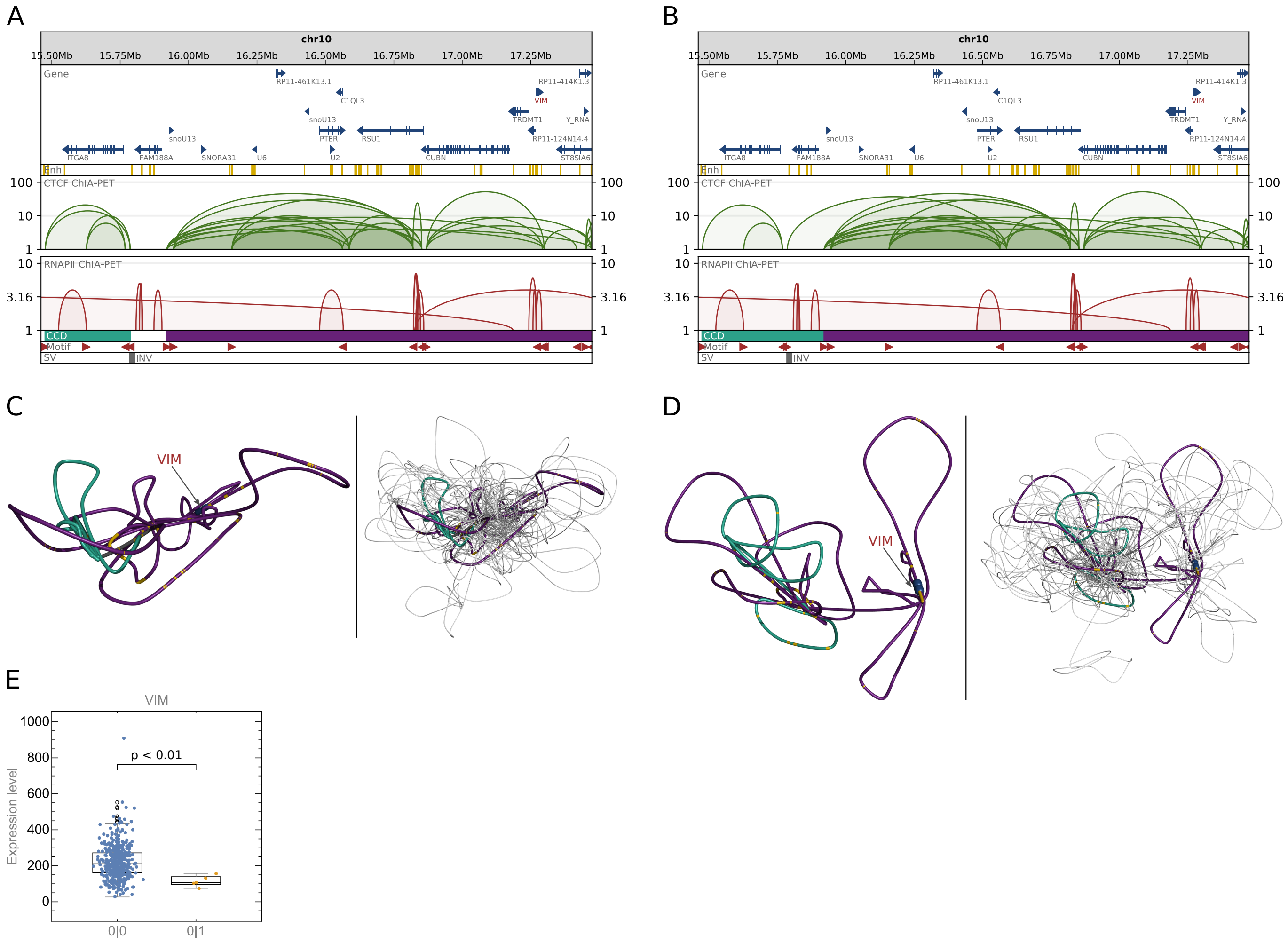

Fig. S11: Inversion of a CCD border in region chr10:15460694-17473625. **a** ChIA-PET interactions at the inversion chr10:15784798-15802449 site in genomes not carrying the inversion. Orientation of CTCF motifs at the interaction anchors are marked with red arrows. **b** Predicted ChIA-PET interactions at the inversion site in genomes carrying the inversion. **c** Model showing spatial arrangement of chromatin loops in the genomic region shown in (a) (left) and an ensemble of such models superimposed (right). **d** Similar to (c), but interaction network shown in (b) is modeled. According to our model, CTCF anchor after the inversion loses the old contacts and is linked to the closest interaction anchor that can form a convergent loop with it. Since the anchor affected by the inversion forms convergent loop encompassing the domain, the domain boundary is lost after the inversion. Interestingly, it seems that the motif inversion leads to a topology with two domains, but different than those before inversion. The smaller of the two borders contains the VIM gene, which transcription drops with the inversion. **e** The VIM gene which transcription correlates with occurrence of the inversion (p-value from the Mann-Whitney test).

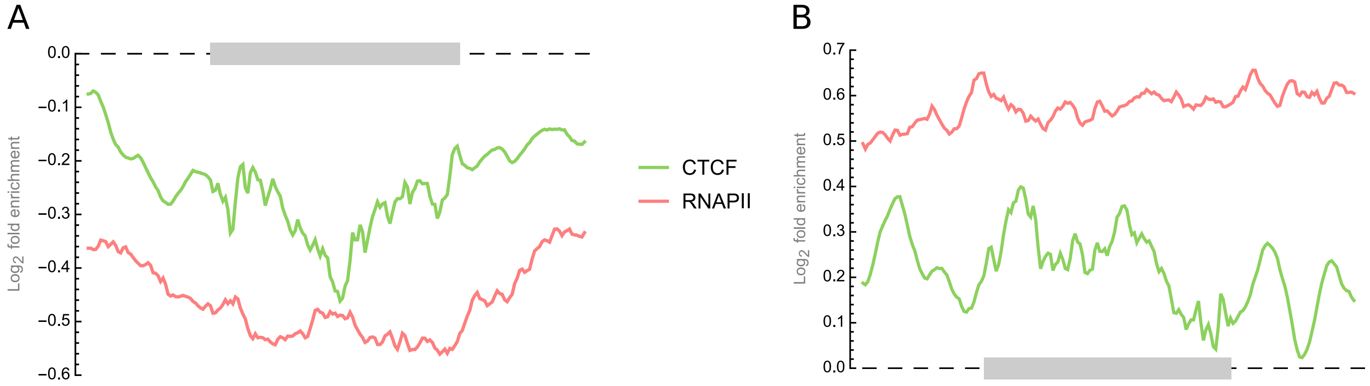

Fig. S12: Aggregate analysis of ChIP-seq signals in altered interaction anchors. **a** Enrichment/depletion of the CTCF and RNAPII ChIP-seq signal in anchors hit by deletions. **b** Enrichment/depletion of the CTCF and RNAPII ChIP-seq signal in anchors hit by duplications.

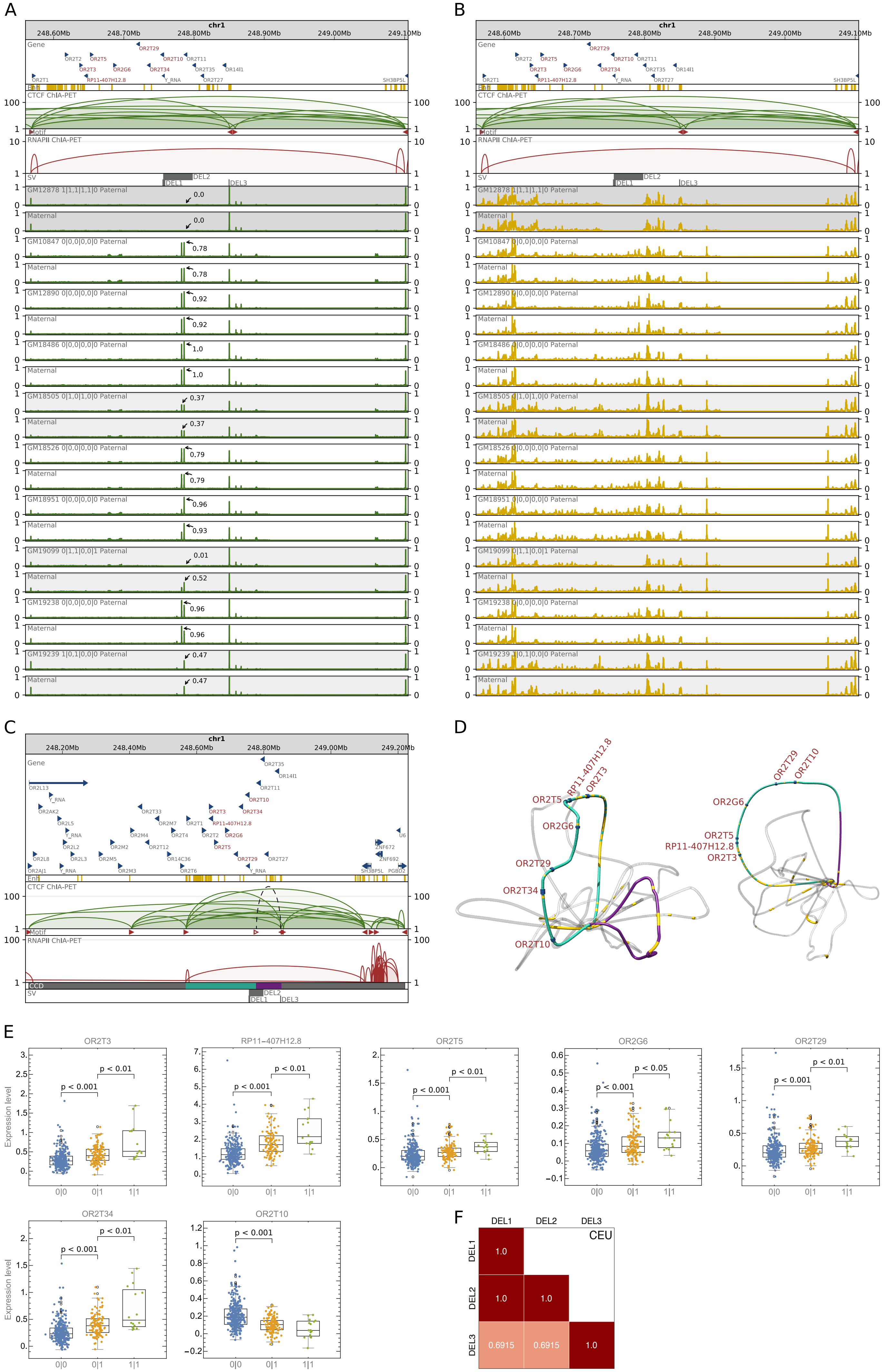
A
B
C
D
E
F
chr1
248.60Mb
248.70Mb
248.80Mb
248.90Mb
249.00Mb
249.10Mb
Gene
Enh
CTCF ChIA-PET
Motif
RNAPII ChIA-PET
SV
DEL1
DEL2
DEL3
GM12878 1|1,1|1,1|0 Paternal
GM10847 0|0,0|0,0|0 Paternal
GM12890 0|0,0|0,0|0 Paternal
GM18486 0|0,0|0,0|0 Paternal
GM18505 0|1,0|1,0|0 Paternal
GM18526 0|0,0|0,0|0 Paternal
GM18951 0|0,0|0,0|0 Paternal
GM19099 0|1,1|0,0|1 Paternal
GM19238 0|0,0|0,0|0 Paternal
GM19239 1|0,1|0,0|0 Paternal
Maternal
248.20Mb
248.40Mb
248.60Mb
248.80Mb
249.00Mb
249.20Mb
CCD
OR2T3
RP11-407H12.8
OR2T5
OR2G6
OR2T29
OR2T34
OR2T10
Expression level
p < 0.001
p < 0.01
p < 0.05
0|0
0|1
1|1
CEU
1.0
0.6915

Fig. S13: Deletions introducing architectural changes at olfactory receptor family genes locus. **a** ChIA-PET interactions at the deletion site chr1:248560000-249105000 displayed above the haplotype-specific CTCF ChIP-seq profiles for a number of lymphoblastoid cells. For each track, ChIP-seq signal values (originally in RPMs) were divided by the maximal value of the signal in the visualized region. In each signal track, value of the highest signal peak in genomic region targeted by a SV is additionally marked. **b** Similar to (a), but H3K4me1 ChIP-seq signals are presented. **c** ChIA-PET interactions forming the topological domain around the deletion site. Predicted chromatin loop disrupted in GM12878 cell by DEL2 is represented by the black dotted line. **d** Models showing enhancers and selected genes relative arrangement in 3D within topological domain shown in (c). Topology of genomes without the deletions (left) and with the deletions (right). DEL2 removes a potential insulation boundary between a group of olfactory receptor family genes (light green region) and a group of enhancers (magenta region). There is a strong CTCF motif at the positive strand of DNA fragment covered by the deletion. The loss of the insulation site may promote higher transcription rates of those genes (we did not obtain transcription rates for OR2T11, OR2T27 and Y_RNA). DEL1 (in a very strong LD with DEL2) intersects the OR2T10 gene, which may be the reason why OR2T10 is the only gene which transcription drops with occurrence of the deletions. DEL3 excises a strong CTCF binding motif in a interaction anchor. However it is not reflected in the CTCF signal and requires experiments to gain more certainty about the details of the chromatin topology in the region. Interestingly, it seems that there are no RNAPII chromatin loops within the CTCF loop containing the genes, which may suggest that potential spatial interactions between those genes and enhancers are regulated by CTCF. **e** Genes which transcription correlates with occurrence of DEL2 (p-values from the Mann-Whitney test). **f** Linkage disequilibrium (measured as r2 value in the CEU population) between the deletions shown in (a), (b) and (c).

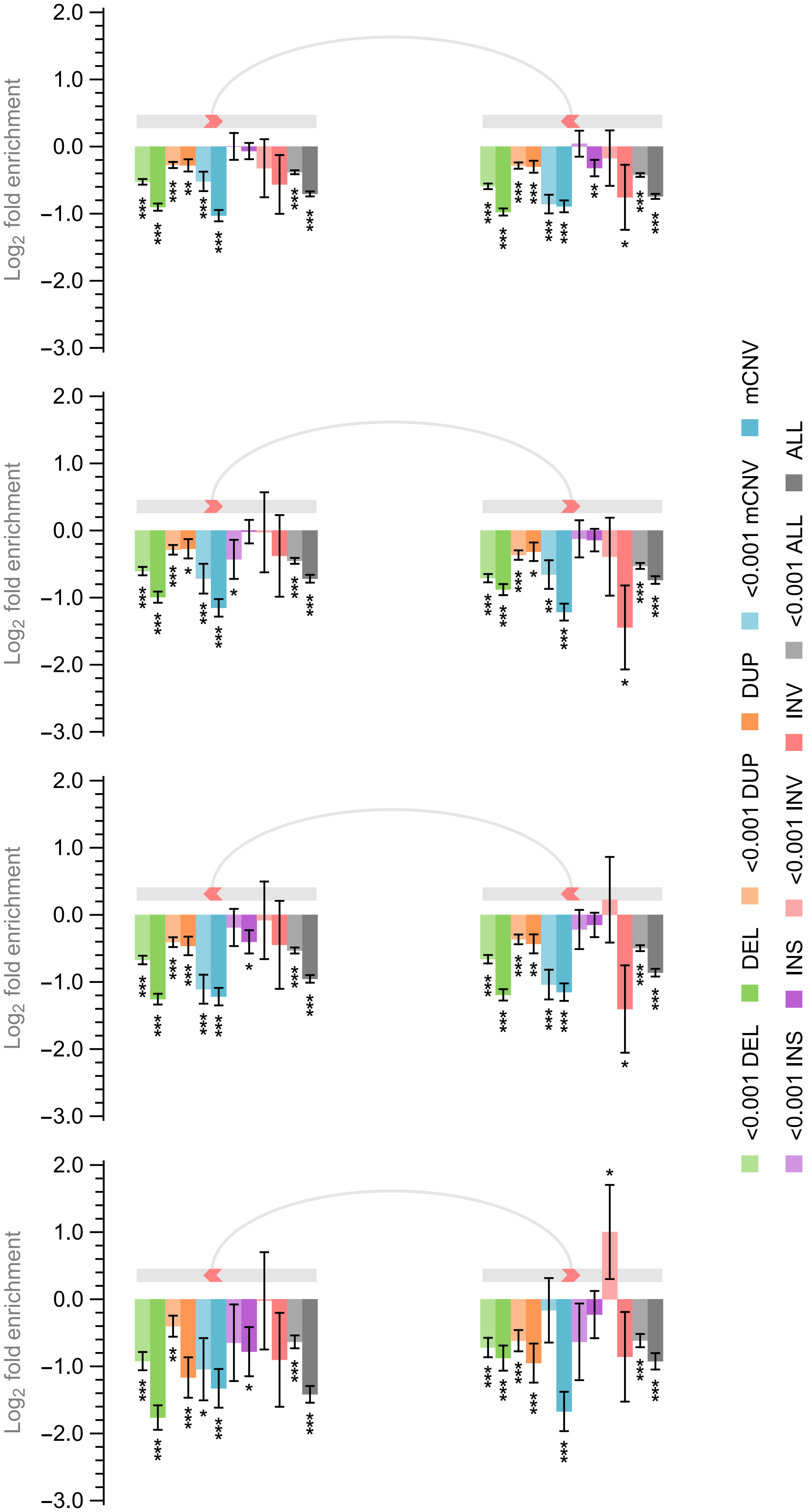

Fig. S14: Impact of structural variants on CTCF ChIA-PET interactions in GM12878 cells. Enrichement/depletion of anchors of CTCF PET clusters with SVs of different types and of different VAF (VAF < 0.001 and VAF $\geqslant 0.001$). Calculations for convergent, tandem right, tandem left and divergent loops are plotted separately.

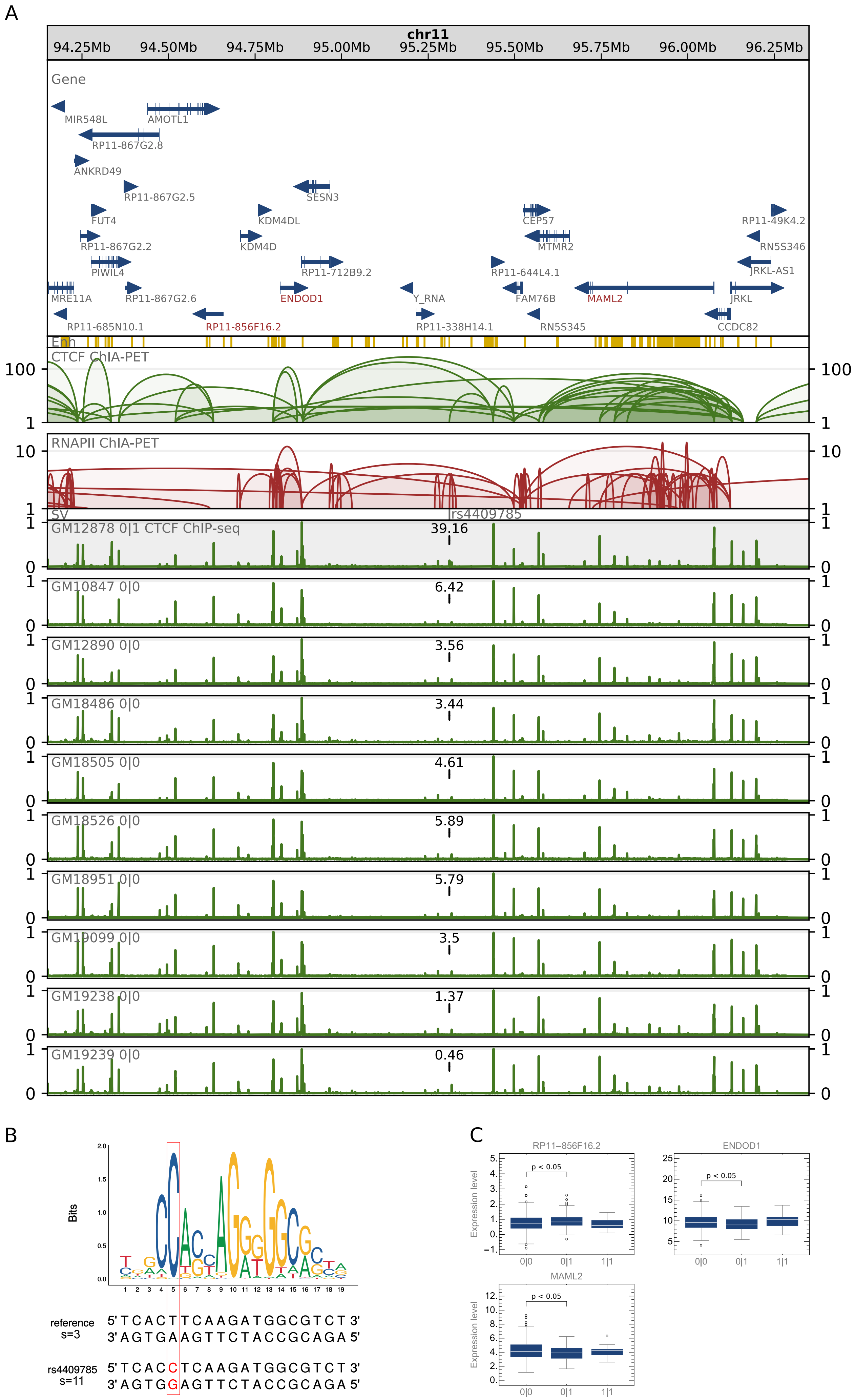

A
chr11
94.25Mb 94.50Mb 94.75Mb 95.00Mb 95.25Mb 95.50Mb 95.75Mb 96.00Mb 96.25Mb
Gene
MIR548L
AMOTL1
RP11-867G2.8
ANKRD49
RP11-867G2.5
SESN3
FUT4
KDM4DL
CEP57
RP11-49K4.2
RP11-867G2.2
KDM4D
MTMR2
RN5S346
PIWIL4
RP11-712B9.2
RP11-644L4.1
JRKL-AS1
MRE11A
RP11-867G2.6
ENDOD1
Y_RNA
FAM76B
MAML2
JRKL
RP11-685N10.1
RP11-856F16.2
RP11-338H14.1
RN5S345
CCDC82
Enh
CTCF ChIA-PET
RNAPII ChIA-PET
SV
rs4409785
GM12878 0|1 CTCF ChIP-seq
39.16
GM10847 0|0
6.42
GM12890 0|0
3.56
GM18486 0|0
3.44
GM18505 0|0
4.61
GM18526 0|0
5.89
GM18951 0|0
5.79
GM19099 0|0
3.5
GM19238 0|0
1.37
GM19239 0|0
0.46
B
Bits
reference
s=3
5' T C A C T T C A A G A T G G C G T C T 3'
3' A G T G A A G T T C T A C C G C A G A 5'
rs4409785
s=11
5' T C A C C T C A A G A T G G C G T C T 3'
3' A G T G G A G T T C T A C C G C A G A 5'
C
RP11−856F16.2
ENDOD1
MAML2
Expression level
p < 0.05
0|0
0|1
1|1

Fig. S15: GWAS SNP in a CTCF motif in region chr11:94150000-96350000. **a** Browser view of a 2 Mb genomic segment with SNP rs4409785 identified in a part of the human population and associated with rheumatoid arthritis and vitiligo. SNP rs4409785 alters sequence of a CTCF motif residing in an interaction anchor. CTCF ChIP-seq signals from 10 lymphoblastoid cell lines genotyped for this SNP are presented for comparison along with genes, enhancers and CTCF and RNAPII ChIA-PET interactions located in this genomic segment. ChIP-seq signal from each sample is measured in RPMs and divided by the maximal value of the signal in the visualized region. In each individual signal track, value of the signal summed over genomic region occupied by the altered CTCF anchor is additionally marked. **b** Comparison of sequences and scores of CTCF motifs with reference and alternative alleles defined by rs4409785. CTCF sequence logo demonstrates the importance of particular nucleotide positions in the motif. **c** Differences in gene transcription rates between genotypes set for rs4409785. Only genes exhibiting differences in transcription which pass Mann-Whitney test with p-value < 0.05 were reported.

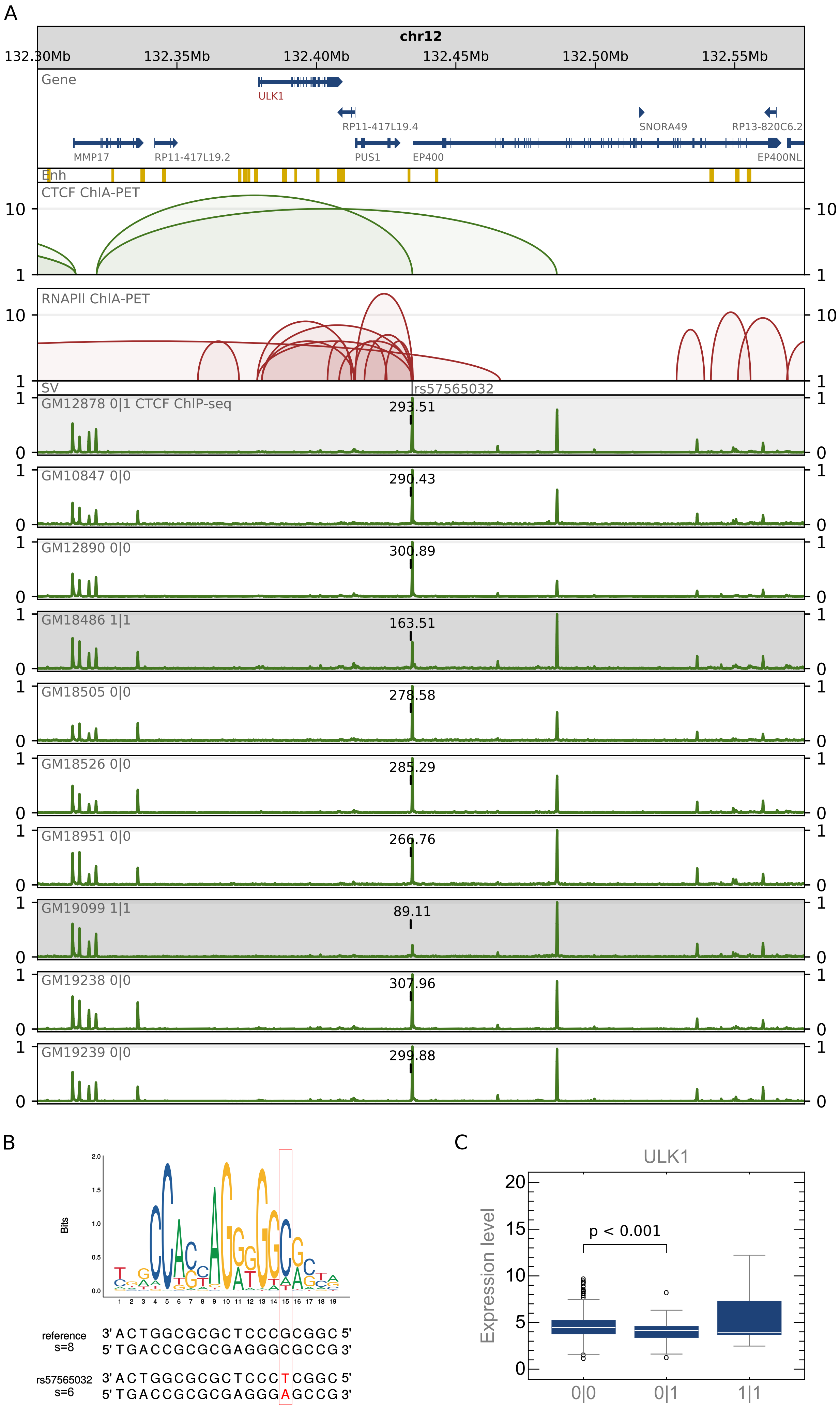
A
chr12
132.30Mb
132.35Mb
132.40Mb
132.45Mb
132.50Mb
132.55Mb
Gene
ULK1
RP11-417L19.4
SNORA49
RP13-820C6.2
MMP17
RP11-417L19.2
PUS1
EP400
EP400NL
Enh
CTCF ChIA-PET
RNAPII ChIA-PET
SV
rs57565032
GM12878 0|1 CTCF ChIP-seq
293.51
GM10847 0|0
290.43
GM12890 0|0
300.89
GM18486 1|1
163.51
GM18505 0|0
278.58
GM18526 0|0
285.29
GM18951 0|0
266.76
GM19099 1|1
89.11
GM19238 0|0
307.96
GM19239 0|0
299.88
B
Bits
reference
s=8
3' A C T G G C G C G C T C C C G C G G C 5'
5' T G A C C G C G C G A G G G C G C C G 3'
rs57565032
s=6
3' A C T G G C G C G C T C C C T C G G C 5'
5' T G A C C G C G C G A G G G A G C C G 3'
C
ULK1
Expression level
p < 0.001
0|0
0|1
1|1

Fig. S16: GWAS SNP in a CTCF motif in region chr12:132300000-132575000. **a** Browser view of a 0.3 Mb genomic segment with SNP rs57565032 identified in a part of the human population and associated with red blood cell distribution width. SNP rs57565032 alters sequence of a CTCF motif residing in an interaction anchor. CTCF ChIP-seq signals from 10 lymphoblastoid cell lines genotyped for this SNP are presented for comparison along with genes, enhancers and CTCF and RNAPII ChIA-PET interactions located in this genomic segment. ChIP-seq signal from each sample is measured in RPMs and divided by the maximal value of the signal in the visualized region. In each individual signal track, value of the signal summed over genomic region occupied by the altered CTCF anchor is additionally marked. **b** Comparison of sequences and scores of CTCF motifs with reference and alternative alleles defined by rs57565032. CTCF sequence logo demonstrates the importance of particular nucleotide positions in the motif. **c** Transcription of gene ULK1 by genotypes set for rs57565032.

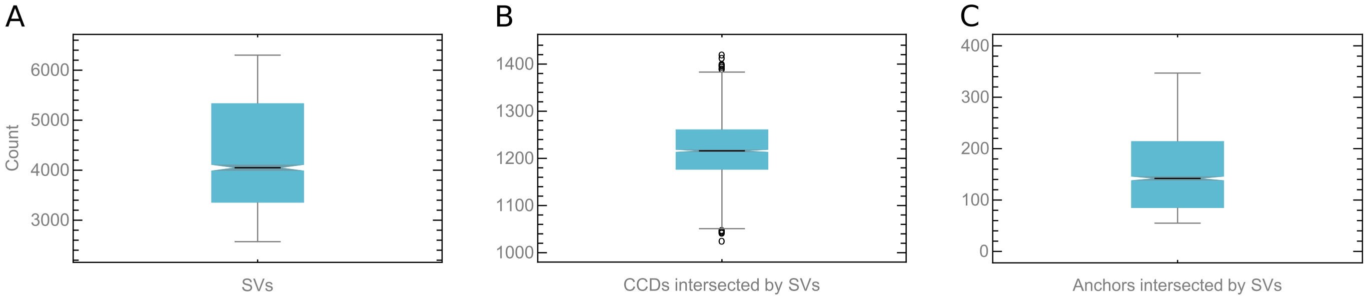

Fig. S17: Quantification of genomic elements in individual genomes. **a** Number of SVs (deletions, duplications, inversions and insertions adopted from the 1000 Genomes Project) per sample. **b** Number of the reference CCDs intersected with SVs in other lymphoblastoid cells. **c** Number of the reference CTCF-mediated interaction anchors intersected with SVs in other lymphoblastoid cells.

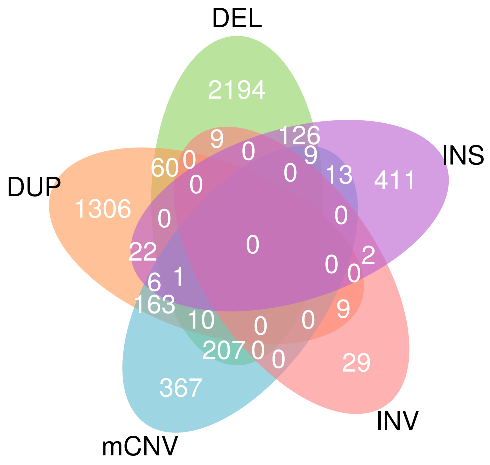

Fig. S18: Division of domain variability patterns by SV classes they contain.

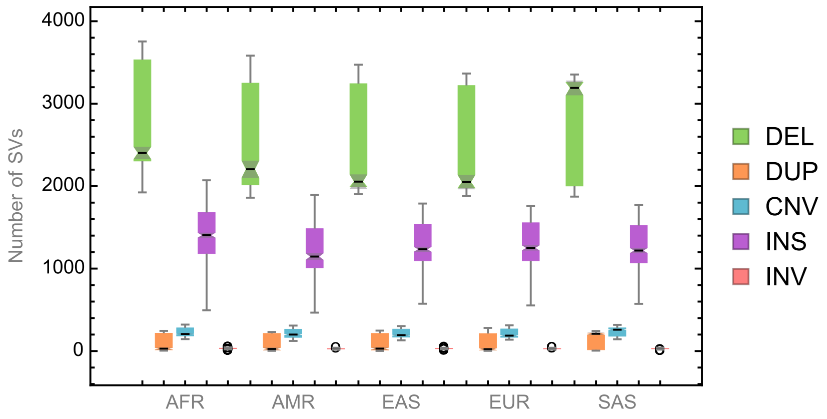

Fig. S19: Number of SVs per individual genome, grouped by SV class and population.

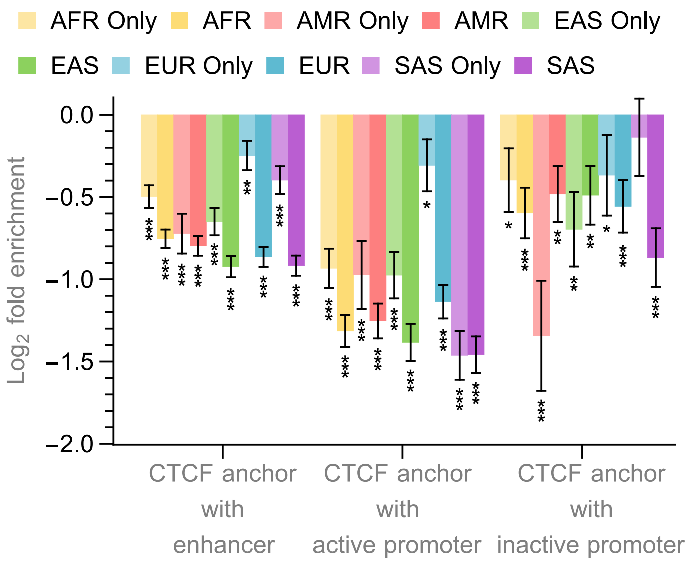

Fig. S20: Enrichment/depletion of CTCF anchors intersected with genomic functional elements identified in GM12878 cells with SVs divided by continental groups.

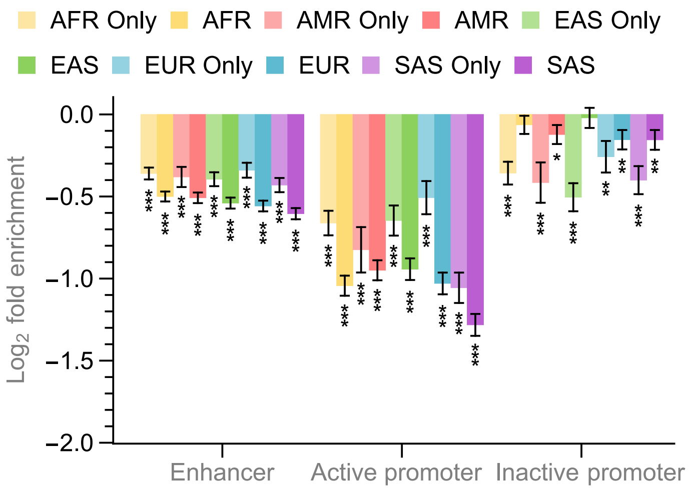

Fig. S21: Enrichment/depletion of genomic functional elements identified in GM12878 cells with SVs divided by continental groups.

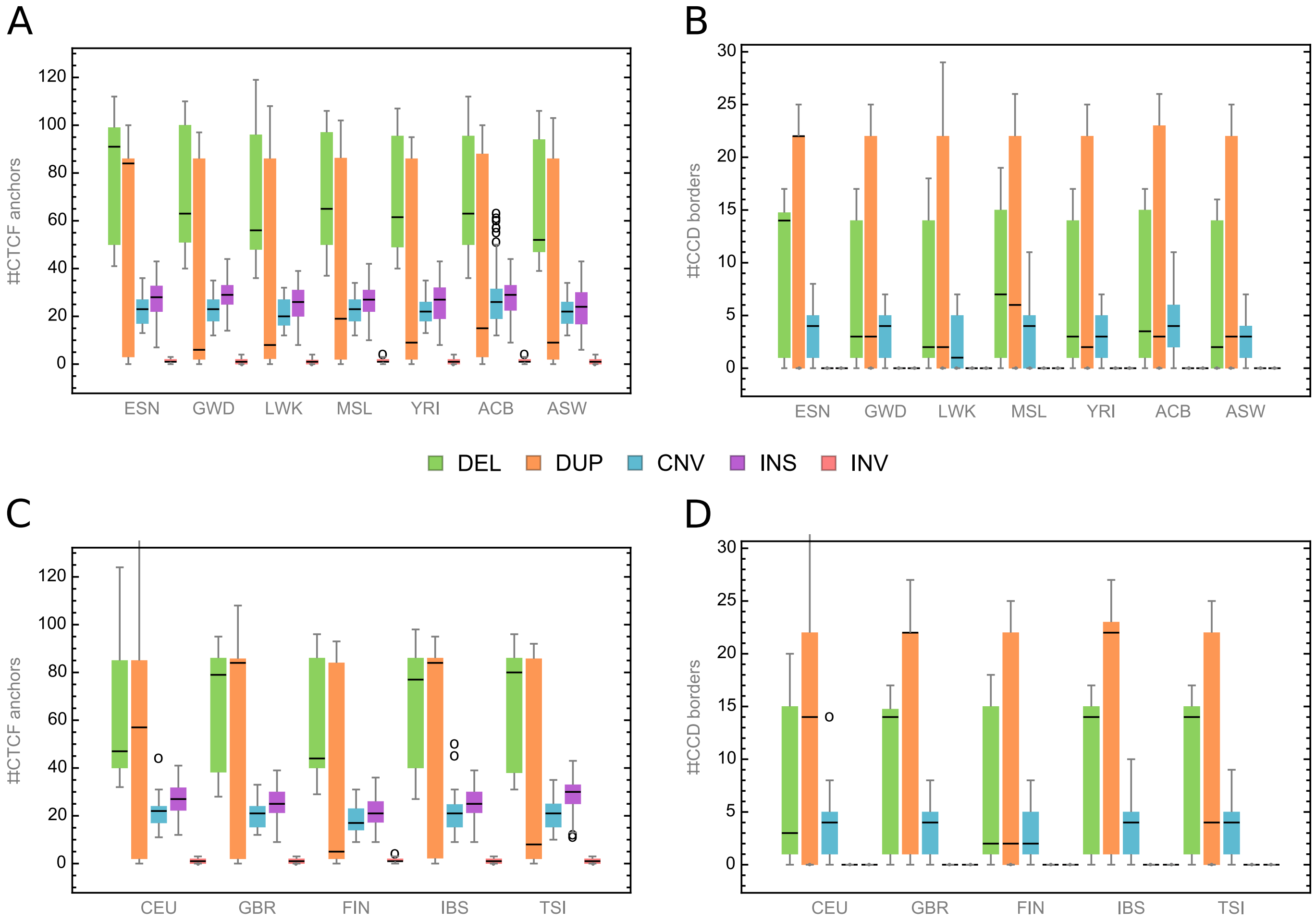

Fig. S22: Individual structural variation in African and European continental groups. **a** Number of CTCF anchors intersected by SVs of a given type identified in individuals from African continental group. **b** Number of domain borders fully overlapped by SVs of a given type identified in individuals from African continental group. **c** Similar to (a), but for European continental group. **d** Similar to (b), but for European continental group.

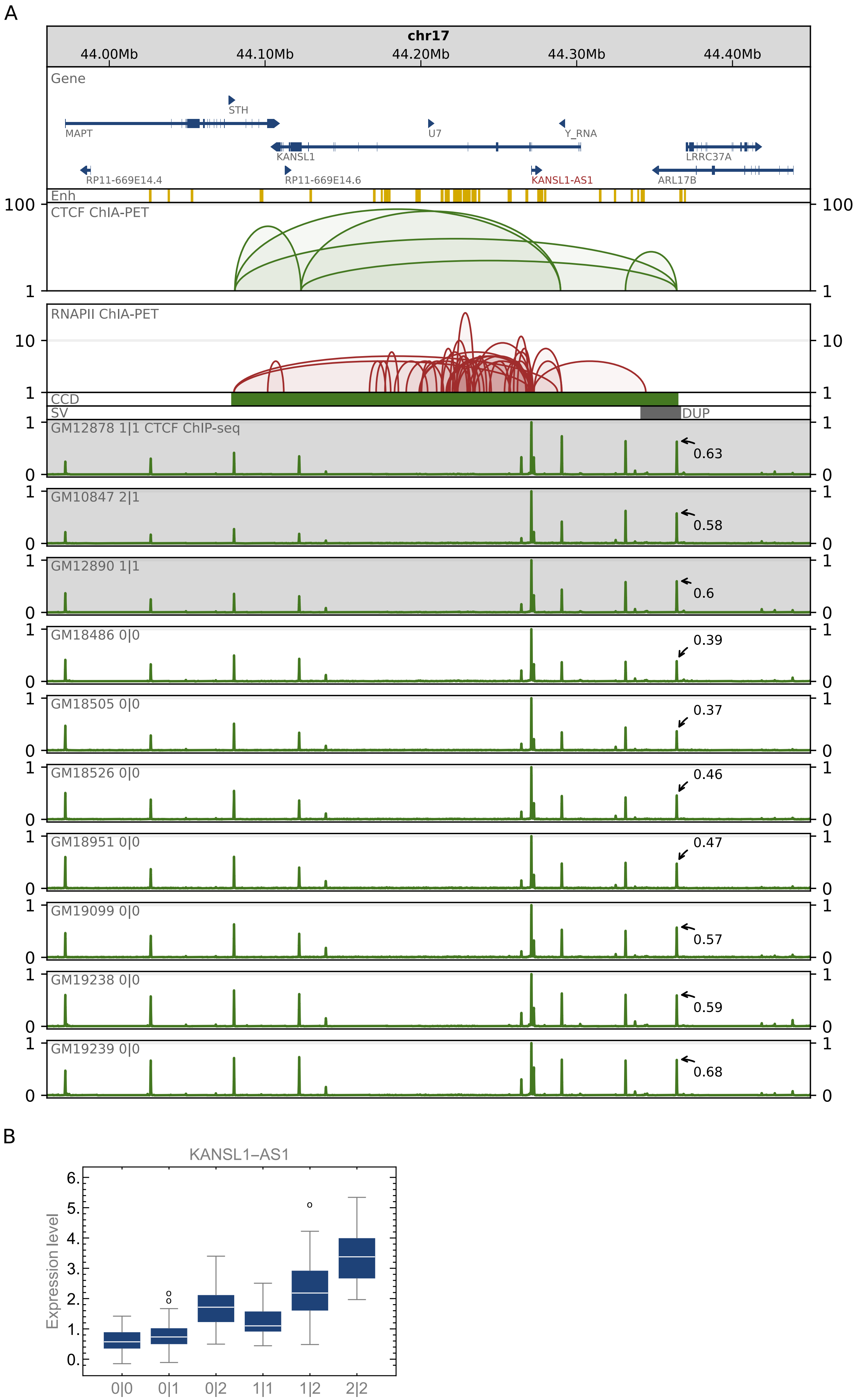
A
chr17
44.00Mb
44.10Mb
44.20Mb
44.30Mb
44.40Mb
Gene
STH
MAPT
U7
Y_RNA
KANSL1
LRRC37A
RP11-669E14.4
RP11-669E14.6
KANSL1-AS1
ARL17B
Enh
CTCF ChIA-PET
RNAPII ChIA-PET
CCD
SV
DUP
GM12878 1|1 CTCF ChIP-seq
0.63
GM10847 2|1
0.58
GM12890 1|1
0.6
GM18486 0|0
0.39
GM18505 0|0
0.37
GM18526 0|0
0.46
GM18951 0|0
0.47
GM19099 0|0
0.57
GM19238 0|0
0.59
GM19239 0|0
0.68
B
KANSL1–AS1
Expression level
0|0
0|1
0|2
1|1
1|2
2|2

Fig. S23: Duplication of a CCD border in region chr17:43960000-44450000. a Browser view of a 0.5 Mb genomic segment with duplication chr17:44341412-44366497 identified in a part of the human population, which duplicates border of a topological domain and is an eQTL for the KANSL1-AS1 gene. CTCF ChIP-seq signals from 10 lymphoblastoid cell lines genotyped for this duplication are presented for comparison along with genes, enhancers and CTCF and RNAPII ChIA-PET interactions located in this genomic segment. ChIP-seq signal from each sample is measured in RPMs and divided by the maximal value of the signal in the visualized region. In each individual signal track, value of the highest signal peak in genomic region targeted by the SV is additionally marked. **b** Transcription of the KANSL1-AS1 gene by genotypes.

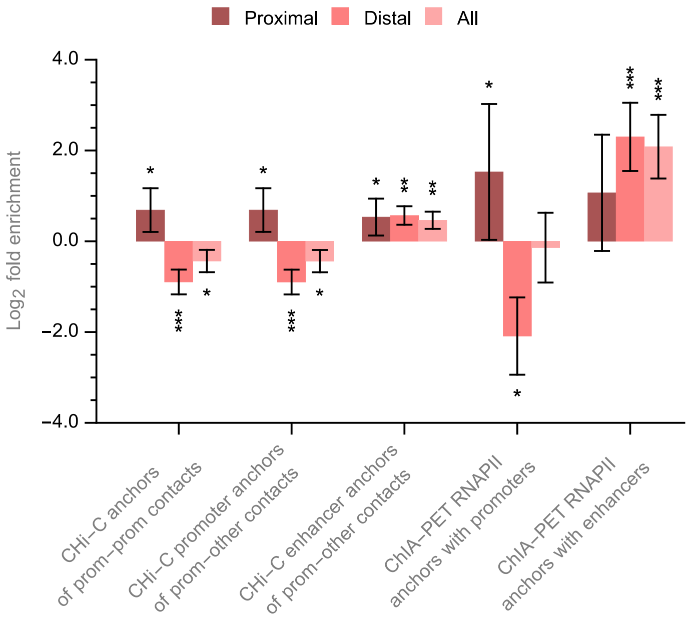

Fig. S24: eQTLs versus promoter interactions. Enrichment/depletion of ChIA-PET and CHi-C anchors containing promoters or enhancers with proximal and distal eQTLs. CHi-C anchors were divided into 3 groups: anchors of promoter-promoter interactions, promoter anchors of promoter-other region interactions and anchors intersected by enhancers of promoter-other region interactions.

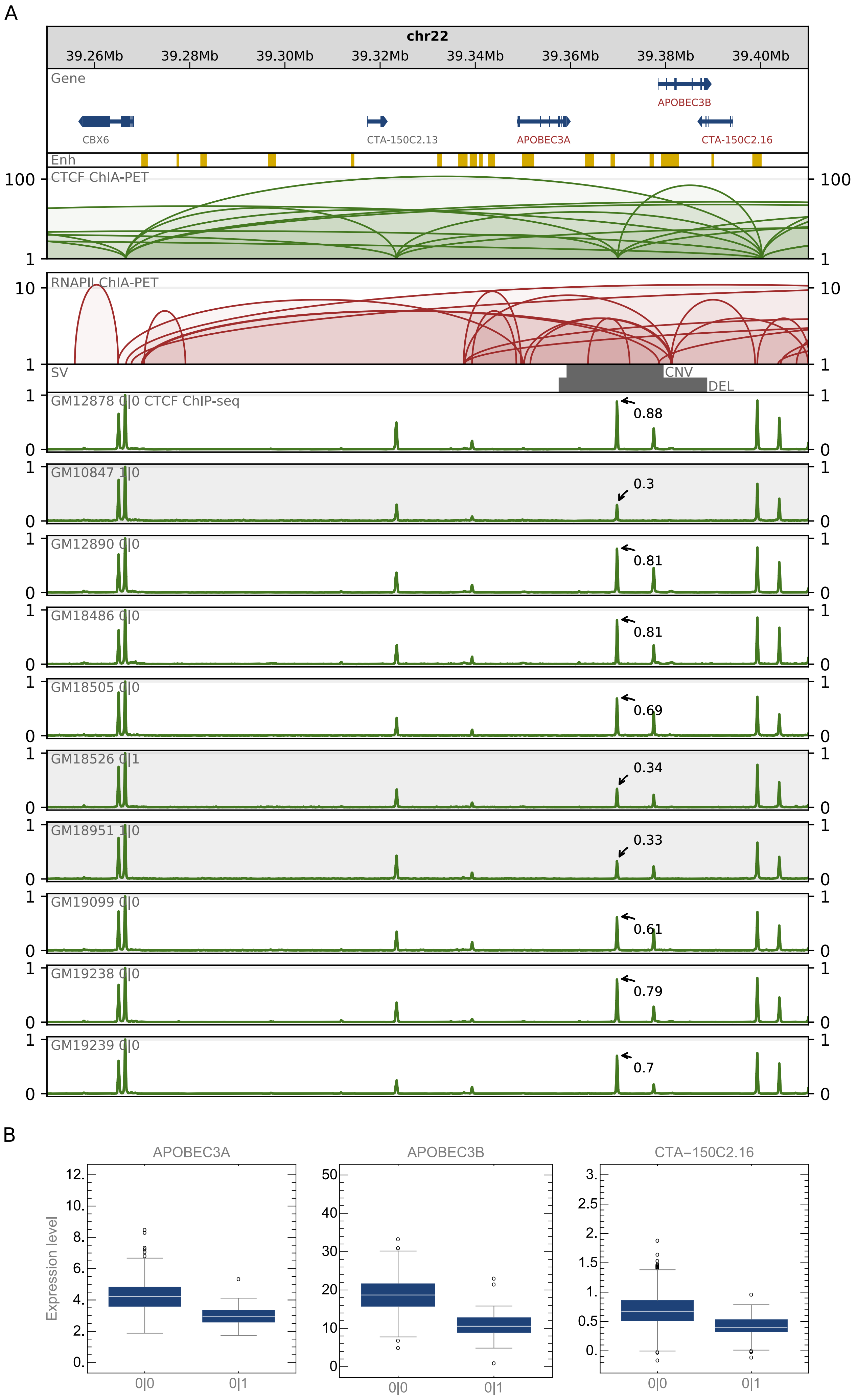
A
chr22
39.26Mb 39.28Mb 39.30Mb 39.32Mb 39.34Mb 39.36Mb 39.38Mb 39.40Mb
Gene
APOBEC3B
CBX6
CTA-150C2.13
APOBEC3A
CTA-150C2.16
Enh
CTCF ChIA-PET
RNAPII ChIA-PET
SV
CNV
DEL
GM12878 0|0 CTCF ChIP-seq
0.88
GM10847 1|0
0.3
GM12890 0|0
0.81
GM18486 0|0
0.81
GM18505 0|0
0.69
GM18526 0|1
0.34
GM18951 1|0
0.33
GM19099 0|0
0.61
GM19238 0|0
0.79
GM19239 0|0
0.7
B
APOBEC3A
APOBEC3B
CTA−150C2.16
Expression level
0|0
0|1

Fig. S25: Deletion of a CTCF interaction anchor in region chr22:39250000-39410000. **a** Browser view of a 0.2 Mb genomic segment with deletion chr22:39357694-39388574/CNV chr22:39359355-39379392 identified in a part of the human population, which removes/removes or multiplies CTCF anchor and is an eQTL for 3 neighboring genes (signed with the red font). APOBEC3A and APOBEC3B are immune-related genes. CTCF ChIP-seq signals from 10 lymphoblastoid cell lines are presented for comparison along with genes, enhancers and CTCF and RNAPII ChIA-PET interactions located in this genomic segment. All the presented cell lines with non-reference genotypes carry deletion chr22:39357694-39388574. ChIP-seq signal from each sample is measured in RPMs and divided by the maximal value of the signal in the visualized region. In each individual signal track, value of the highest signal peak in genomic region targeted by the SV is additionally marked. **b** Genes which transcription is correlated with deletion chr22:39357694-39388574 and CNV chr22:39359355-39379392 (p-value much smaller than 0.001).

A

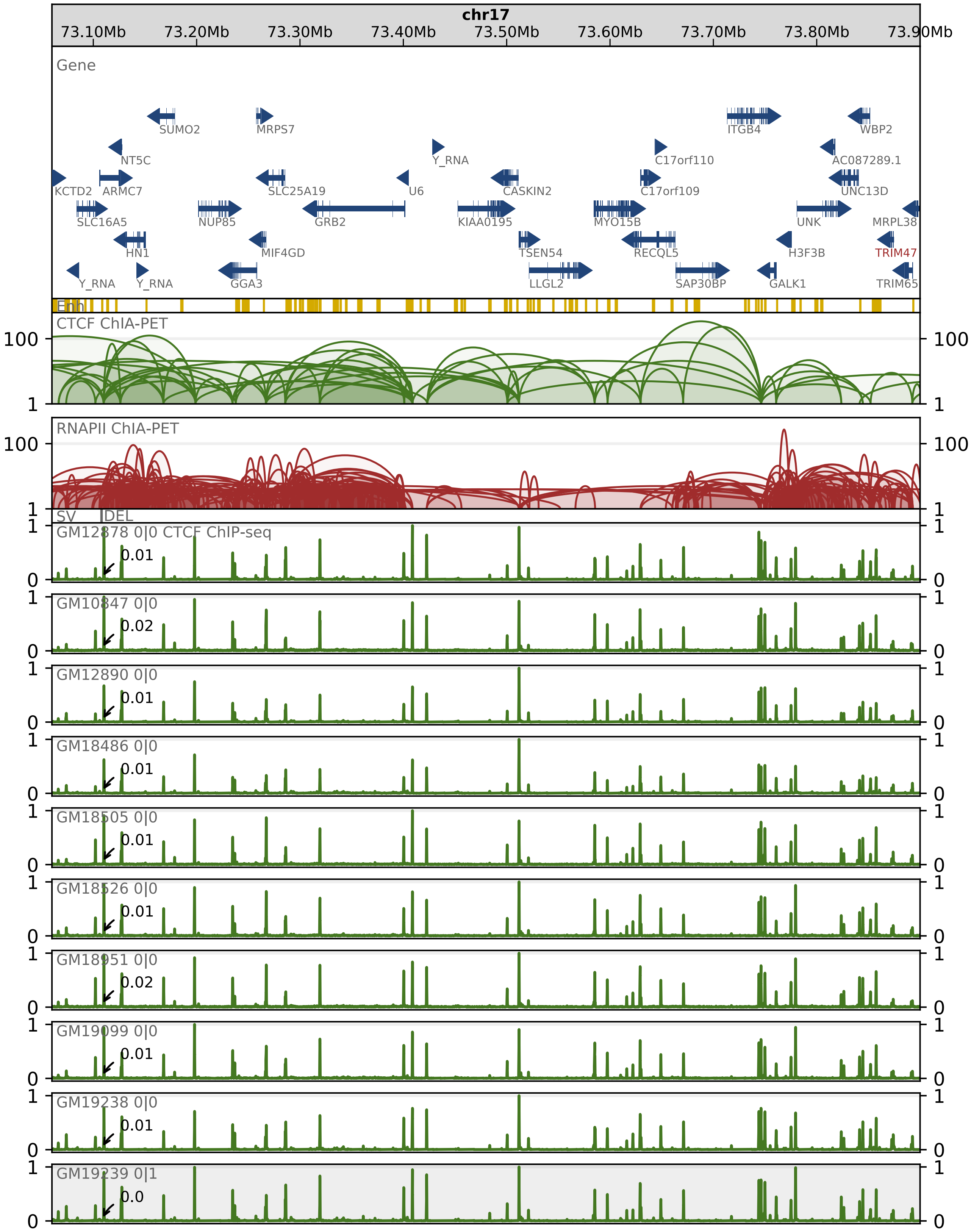
chr17
73.10Mb
73.20Mb
73.30Mb
73.40Mb
73.50Mb
73.60Mb
73.70Mb
73.80Mb
73.90Mb
Gene
SUMO2
MRPS7
ITGB4
WBP2
NT5C
Y_RNA
C17orf110
AC087289.1
KCTD2
ARMC7
SLC25A19
U6
CASKIN2
C17orf109
UNC13D
SLC16A5
NUP85
GRB2
KIAA0195
MYO15B
UNK
MRPL38
HN1
MIF4GD
TSEN54
RECQL5
H3F3B
TRIM47
Y_RNA
Y_RNA
GGA3
LLGL2
SAP30BP
GALK1
TRIM65
Enh
CTCF ChIA-PET
100
1
RNAPII ChIA-PET
100
1
SV
DEL
GM12878 0|0 CTCF ChIP-seq
0.01
GM10847 0|0
0.02
GM12890 0|0
0.01
GM18486 0|0
0.01
GM18505 0|0
0.01
GM18526 0|0
0.01
GM18951 0|0
0.02
GM19099 0|0
0.01
GM19238 0|0
0.01
GM19239 0|1
0.0
1
0

B

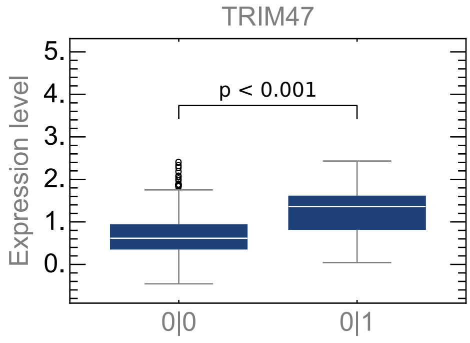
TRIM47
p < 0.001
Expression level
5.
4.
3.
2.
1.
0.
0|0
0|1

Fig. S26: Deletion in a CTCF interaction anchor in region chr17:73060000-73900000. a Browser view of a 0.8 Mb genomic segment with deletion chr17:73107713-73108273 identified in a part of the human population, which removes part of a CTCF anchor and is an eQTL for immune-related gene TRIM47 (signed with the red font). CTCF ChIP-seq signals from 10 lymphoblastoid cell lines genotyped for this deletion are presented for comparison along with genes, enhancers and CTCF and RNAPII ChIA-PET interactions located in this genomic segment. ChIP-seq signal from each sample is measured in RPMs and divided by the maximal value of the signal in the visualized region. In each individual signal track, value of the highest signal peak in genomic region targeted by the SV is additionally marked. **b** Immune-related gene TRIM47 which transcription is correlated with deletion chr17:73107713-73108273 (p-value much smaller than 0.001).

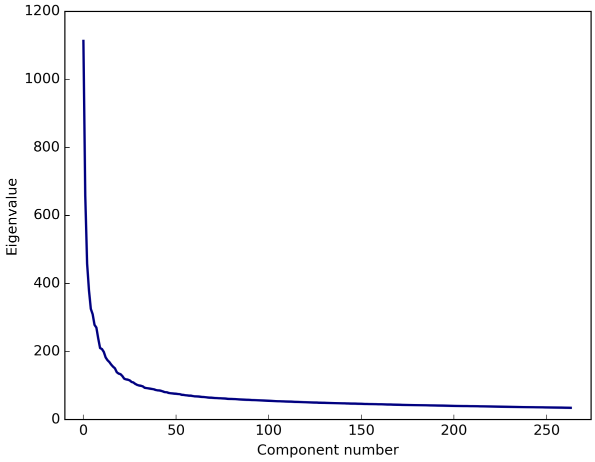

Fig. S27: Scree Plot. Showing sorted eigenvalues for PCA applied to the space of gene expression rates.

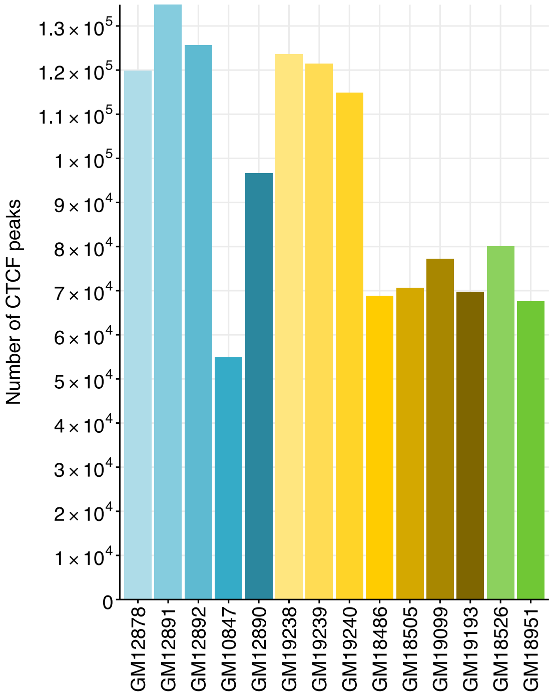

Fig. S28: Number of CTCF ChIP-seq peaks called in different human lymphoblastoid cell lines.

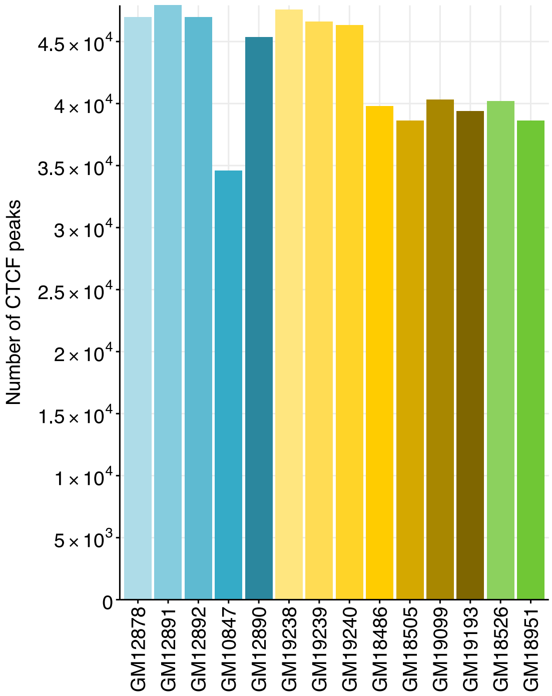

Fig. S29: Number of CTCF ChIP-seq peaks called in different human lymphoblastoid cell lines after filtering by conserved CTCF binding sites.

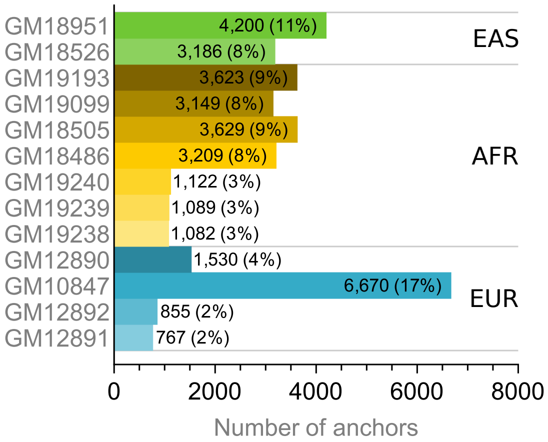

Fig. S30: CTCF anchors from GM12878 not intersected with CTCF ChIP-seq peaks identified in different lymphoblastoid cells.
